# Molecular Targets of Minor Cannabinoids in Breast Cancer: In Silico and In Vitro Studies

**DOI:** 10.3390/ph17091245

**Published:** 2024-09-21

**Authors:** Cristina Ferreira Almeida, Andreia Palmeira, Maria João Valente, Georgina Correia-da-Silva, Anne Marie Vinggaard, Maria Emília Sousa, Natércia Teixeira, Cristina Amaral

**Affiliations:** 1 UCIBIO—Applied Molecular Biosciences Unit, Laboratory of Biochemistry, Department of Biological Sciences, Faculty of Pharmacy, University of Porto, Rua Jorge Viterbo Ferreira, n° 228, 4050-313 Porto, Portugal; cristina-almeida96@hotmail.com (C.F.A.); george@ff.up.pt (G.C.-d.-S.); natercia@ff.up.pt (N.T.); 2 Associate Laboratory i4HB, Institute for Health and Bioeconomy, Faculty of Pharmacy, University of Porto, Rua Jorge Viterbo Ferreira, n° 228, 4050-313 Porto, Portugal; 3 Laboratory of Organic and Pharmaceutical Chemistry, Department of Chemical Sciences, Faculty of Pharmacy, University of Porto, Rua Jorge Viterbo Ferreira, n° 228, 4050-313 Porto, Portugal; apalmeira@ff.up.pt (A.P.); esousa@ff.up.pt (M.E.S.); 4 CIIMAR—Interdisciplinary Centre of Marine and Environmental Research, Av. General Norton de Matos S/N, 4450-208 Matosinhos, Portugal; 5 National Food Institute, Technical University of Denmark, 2800 Kongens Lyngby, Denmark; mjopo@food.dtu.dk (M.J.V.); annv@food.dtu.dk (A.M.V.)

**Keywords:** phytocannabinoids, medicinal cannabis, breast cancer, aromatase, estrogen receptor, androgen receptor

## Abstract

Background: Breast cancer therapy has been facing remarkable changes. Classic treatments are now combined with other therapies to improve efficacy and surpass resistance. Indeed, the emergence of resistance demands the development of novel therapeutic approaches. Due to key estrogen signaling, estrogen receptor-positive (ER^+^) breast cancer treatment has always been focused on aromatase inhibition and ER modulation. Lately, the effects of phytocannabinoids, mainly Δ^9^-tetrahydrocannabinol (THC) and cannabidiol (CBD), have been evaluated in different cancers, including breast. However, *Cannabis sativa* contains more than 120 phytocannabinoids less researched and understood. Methods: Here, we evaluated, both in silico and in vitro, the ability of 129 phytocannabinoids to modulate important molecular targets in ER^+^ breast cancer: aromatase, ER, and androgen receptor (AR). Results: In silico results suggested that some cannabinoids may inhibit aromatase and act as ERα antagonists. Nine selected cannabinoids showed, in vitro, potential to act either as ER antagonists with inverse agonist properties, or as ER agonists. Moreover, these cannabinoids were considered as weak aromatase inhibitors and AR antagonists with inverse agonist action. Conclusions: Overall, we present, for the first time, a comprehensive analysis of the actions of the phytocannabinoids in targets of ER^+^ breast tumors, pointing out their therapeutic potential in cancer and in other diseases.

## 1. Introduction

**Figure 1:**
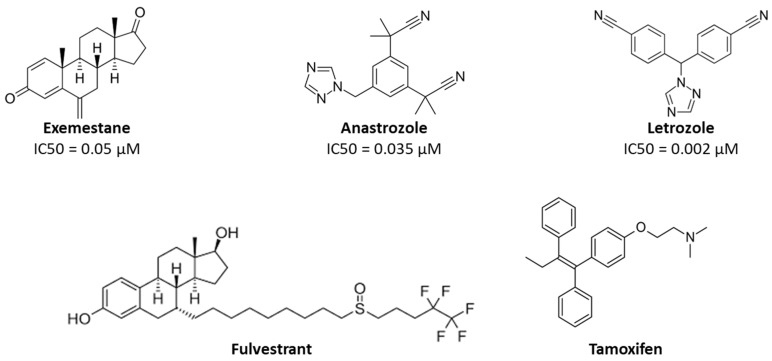
Chemical structures of the AIs, Ana, Let, and Exe, and the anti-estrogens Tamoxifen and Fulvestrant. The IC50 values described for AIs are relative to aromatase and were evaluated in human placental microsomes [25,26].

Despite the continuous advances in drug development, breast cancer incidence keeps rising, having the main responsibility for all cancer-related death among women worldwide [1,2]. Due to their great heterogeneity, breast tumors are grouped in different subtypes, with most of them being classified as estrogen receptor-positive (ER^+^) [3,4,5]. This subtype overexpresses estrogen receptor alpha (ERα) and progesterone receptor (PR). Moreover, in these tumors, the enzyme aromatase is overexpressed, resulting in high rates of local estrogen production [4,6,7]. The elucidation of these targets was pivotal for the development of endocrine therapies, which prevent estrogen production or signaling, such as aromatase inhibitors (AIs), including Anastrozole (Ana), Letrozole (Let), and Exemestane (Exe), as well as anti-estrogens, such as Tamoxifen and Fulvestrant (Figure 1) [8]. In fact, these drugs are part of the therapeutic strategy applied as first-line therapy, both in pre- and post-menopausal status [8,9,10,11,12]. Unfortunately, and despite their clinical success, these compounds are not only associated with adverse side effects, but also with the development of endocrine resistance, which compromises their effectiveness [13,14]. In view of that, alternative therapeutic approaches have been developed to improve treatment, including the introduction of new targeted therapies with novel drugs such as CDK4/6, mTOR, and PI3K inhibitors that are recommended in combination with endocrine therapy [11,12,13,14,15,16,17]. Indeed, the elucidation of the role of additional targets in the development of ER^+^ breast cancer represents a new hope for its treatment. One of the targets that has been considered is the androgen receptor (AR), which is expressed in 70–90% of ER^+^ breast tumors [13,18,19]. This receptor seems to cooperate with ERα and displays different functions depending on the hormonal and resistance status. In ER^+^ breast cancer cases, AR antagonizes ERα signaling, contributing to the decrease in cell proliferation and tumor growth [20]. On the other hand, in cases where resistance to endocrine therapy develops, and thus ERα activity is modified, AR switches its function from tumor suppressor to oncogenic in order to promote cell growth and survival. This emphasizes the existence of a hormone-dependent crosstalk between ERα and AR [21,22,23,24].

**Figure 2:**
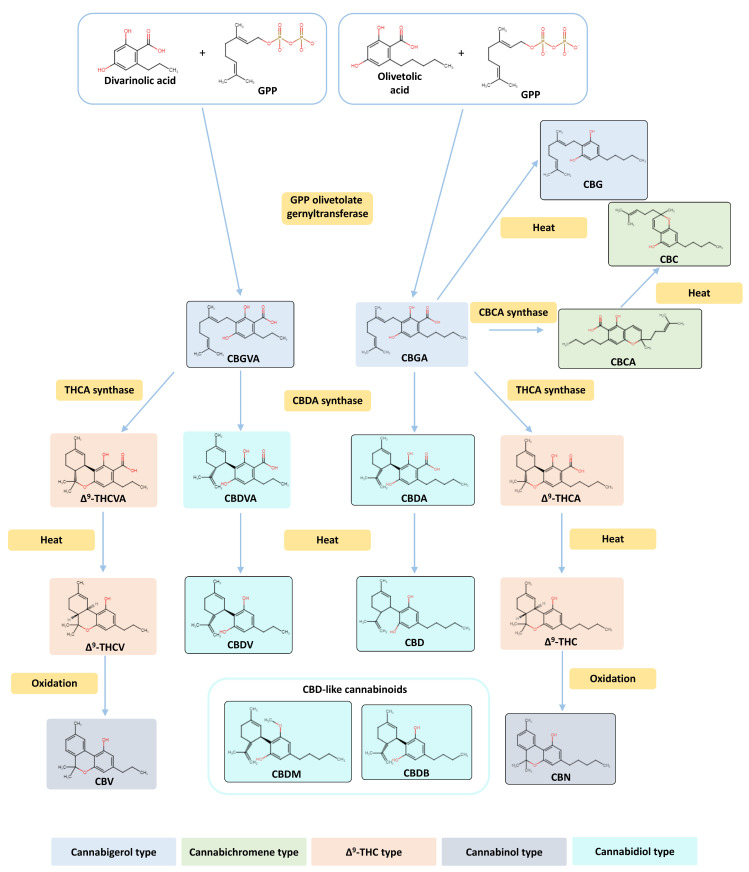
Biosynthetic pathway of phytocannabinoid and phytocannabinoid class representation. Structural and biosynthetic relationships of the nine minor cannabinoids studied in vitro: cannabigerol (**CBG**), cannabidivarin (**CBDV**), cannabinol (**CBN**), cannabidiol-C4 (**CBDB**), cannabidiolic acid (**CBDA**), cannabidiol monomethyl ether (**CBDM**), cannabichromenic acid (**CBCA**), cannabigerovarinic acid (**CBGVA**), and cannabichromene (**CBC**). The remaining phytocannabinoids involved in this pathway, namely cannabidiol (**CBD**), cannabigerolic acid (**CBGA**), Δ^9^-tetrahydrocannabivaric acid (**Δ^9^-THCVA**), Δ^9^-tetrahydrocannabivarin (**Δ^9^-THCV**), Δ^9^-tetrahydrocannabinolic acid (**Δ^9^-THCA**), Δ^9^-tetrahydrocannabinol (**THC**), cannabivarin (**CBV**), and cannabidivarinic acid (**CBDVA**), are also presented. GPP: geranyl diphosphate.

Cannabinoids have demonstrated promising therapeutic actions in different pathological conditions, including epilepsy, asthma, sleep disorders, depression, and inflammation, and in the relief of chemotherapy-associated side effects [27,28,29]. Notably, various studies have attributed anti-proliferative, anti-angiogenic, and anti-invasive actions to cannabinoids in various cancer types [28,30,31,32,33,34,35,36,37]. In fact, the in vitro and in vivo effects of the two best known cannabinoids, Δ^9^-tetrahydrocannabinol (**THC**) and cannabidiol (**CBD**), were already explored in different cancer types, including breast cancers. Usually, the cannabinoids exert anti-tumor effects through the inhibition of cell growth, neovascularization, migration, adhesion, invasion, and metastasis, and also by promoting cell cycle arrest, autophagy, and apoptosis [33,38,39,40,41,42,43,44,45,46,47,48,49,50,51,52,53,54,55,56,57]. To date, the studies conducted on cannabinoids in breast cancer were mainly performed in triple-negative and HER2^+^ tumors [33,58,59,60]. Nevertheless, there is evidence that molecular pathways between cannabinoid receptors and estrogens or androgens may overlap, which may impact ER^+^ breast cancer [60,61]. In 1997, Ruh et al. demonstrated that **THC** and **CBD** fail to act as ER agonists in MCF-7 cells [62]. Years later, it was demonstrated that **THC** impaired proliferation of MCF-7 cells by an ER- and AR-independent mechanism [63], and disrupted estrogen-signaling by up-regulating ERβ [55]. More recently, our group has clarified the anti-cancer role of **CBD** and **THC**, and of the endocannabinoid anandamide (**AEA**) in MCF-7aro cells, unveiling their mechanism of action and their ability to modulate key targets, such as aromatase, ERα, and ERβ [42,64,65]. Besides this, we have verified that **CBD** and **AEA** fit well at the aromatase binding site, inhibiting its activity by 78.6% and 61.0%, respectively, at 2 μM, in human placental microsomes [64,65], whereas **THC** inhibited 25.6% [65]. Moreover, we recently demonstrated that **CBD** acts as an ER and AR antagonist, with inverse agonist properties at both receptors, depending on hormonal influence. Furthermore, this cannabinoid is able to improve AIs’ anti-proliferative effects in ER^+^ breast cancer cells, with the combination of **CBD** with Exe having potential clinical value as an adjuvant therapy [44]. However, *Cannabis* contains several other phytocannabinoids. In fact, more than 500 different compounds were isolated from the plant and around 140 of them are phytocannabinoids. Regarding *Cannabis sativa*, 129 phytocannabinoids have been described whose therapeutic potential has not been fully understood. They are generally grouped in 11 different classes: the **Δ^9^-THC** type, **Δ^8^-THC** type, **CBD** type, cannabigerol (**CBG**) type, cannabinodiol (**CBND**) type, cannabielsoin (**CBE**) type, cannabicyclol (**CBL**) type, cannabichromene (**CBC**) type, cannabinol (**CBN**) type, cannabitriol (**CBT**) type, and miscellaneous type [27,66,67,68,69]. All the cannabinoids included in the different classes share a similar C21 terpenophenolic backbone and their biosynthesis is mostly derived from the geranyl diphosphate (GPP) prenylation of either divarinolic acid or olivetolic acid, a reaction catalyzed by GPP olivetolate geranyltransferase. As a result, cannabigerovarinic acid (**CBGVA**) and cannabigerolic acid (**CBGA**) are produced, respectively. Through enzymatic and non-enzymatic transformations, **CBGVA** and **CBGA** originate the phytocannabinoids that constitute the five main classes described so far (Figure 2) [70,71]. The pharmacological interest about the minor phytocannabinoids has been growing and the anti-tumor properties of **CBG**, **CBN**, cannabidiolic acid (**CBDA**), cannabidiol-C4 (**CBDB**), and cannabichromenic acid (**CBCA**) were already addressed in diverse types of cancers, including breast [28,45,72,73,74,75,76,77,78,79,80,81]. So far, only the minor phytocannabinoids **CBG** and **CBN** have been studied in breast cancer models [75,76]. Taken together, we aimed to evaluate, in silico and in vitro*,* the ability of minor phytocannabinoids to interact with and modulate important targets in developing ER^+^ breast cancer, namely aromatase, ERα, and AR, to expand our knowledge on these compounds and substantiate the development of new therapeutic solutions. Moreover, we intend to characterize the potential multi-target actions of cannabinoids in this disease.

## 2. Results

### 2.1. Molecular Docking Results

As previously stated, the therapeutic potential of almost all the 129 different phytocannabinoids from the *Cannabis sativa* plant is still unknown. In order to investigate their possible beneficial actions on ER^+^ breast cancer treatment, a molecular docking analysis on aromatase and ERα, two key targets responsible for the development of this disease [6], was performed. Our calculations revealed that 45 compounds are located in the aromatase binding site above the heme group and are stabilized by several residues, such as Arg115, Asp309, Phe221, Trp224, Met374, and Leu372 (Table 1, Supplementary Figure S1), previously reported as important for aromatase inhibition [82,83]. In fact, most of those cannabinoids belong to **Δ^9^-THC**, **CBD**, and miscellaneous types and showed the ability to establish hydrogen bonds with some residues, particularly with Asp309, a fundamental residue for the aromatase reaction [84,85]. According to our calculations, the compounds with the best energy score were cannabicyclol and (-)-Δ^7^-*trans*-(1*R*,3*R*,6*R*)-isotetrahydrocannabinol-C_5_, both presenting a binding affinity of −9.8 kcal/mol.

Regarding ERα, we analyzed the docking positions and the energy scores and selected 85 compounds of the 129 cannabinoids as the best candidates to act as ERα antagonists (Table 2, Supplementary Figure S2). The cannabinoids selected belong mainly to **Δ^9^-THC**, **CBG**, **CBC**, and miscellaneous classes and all of them bind in the vicinity of the residues Val534, Leu525, Glu353, Arg394, Phe404, Met421, His524, and Asp351, being mainly stabilized by hydrophobic interactions. The cannabinoid with the best binding affinity was cannabioxepane, which presented a value of −9.2 kcal/mol.

**Figure 3:**
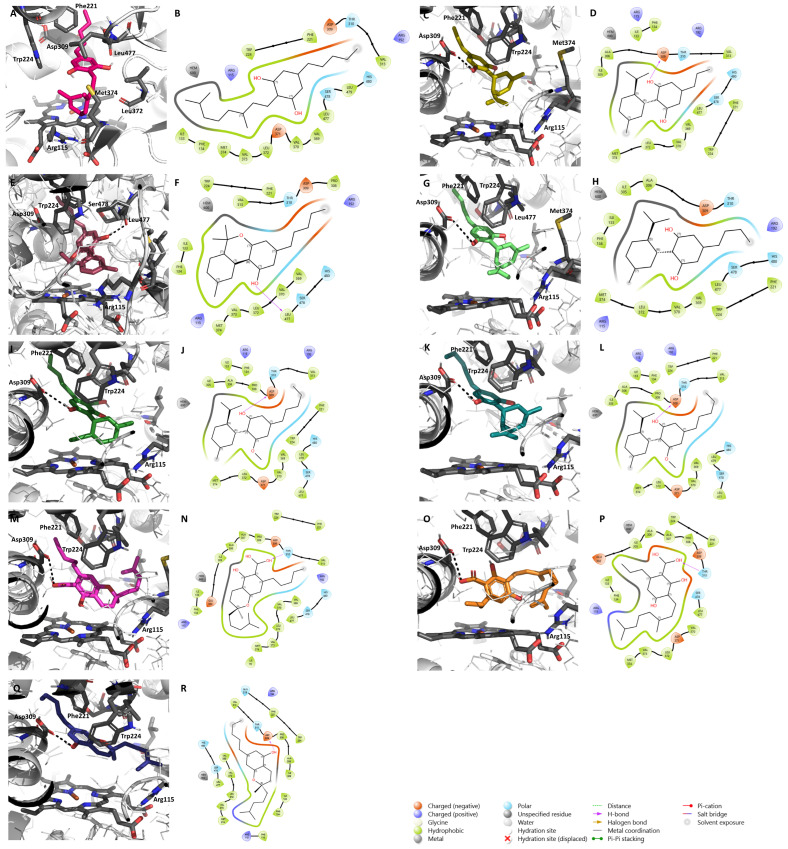
Molecular docking of the nine minor cannabinoids, **CBG** (**A**,**B**), **CBDV** (**C**,**D**), **CBN** (**E**,**F**), **CBDB** (**G**,**H**), **CBDA** (**I**,**J**), **CBDM** (**K**,**L**), **CBCA** (**M**,**N**), **CBGVA** (**O**,**P**), and **CBC** (**Q**,**R**), in aromatase. The images represent both 2D and 3D aromatase/cannabinoid interactions. Aromatase structure has the PDB code 3S79 and is represented in as cartoon. The main amino acid residues of the target, including Asp309, Phe221, Trp224, Arg115, Leu372, Leu477, Ser478, and Met374, as well as the heme group, are shown as gray sticks, while the cannabinoids are shown as different colorful sticks. Hydrogen bonds are represented as black dashed lines in the 3D representations and as purple arrows in the 2D maps.

Considering the overall results observed for these two targets, 36 out of the 129 phytocannabinoids were simultaneously considered as potential AIs and ERα antagonists (Table 3, Supplementary Figure S3). However, only 7 out of those 36 selected cannabinoids, namely **CBDV**, **CBDB**, **CBDA**, **CBDM**, **CBCA**, **CBGVA**, and **CBC** (Figure 2), are commercially available and, thus, were selected for in vitro studies. Furthermore, two other cannabinoids, **CBG** and **CBN** (Figure 2), that did not show potential to act as AIs or ERα antagonists, but have growing pharmacological interest and have been studied in other breast cancer subtypes [75,76], were also included in our study. Figure 2 highlights their structural and biosynthetic relationships. The docking positions of the nine selected minor cannabinoids in aromatase, as well as the most important residues for their binding, are represented in Figure 3.

**Figure 4:**
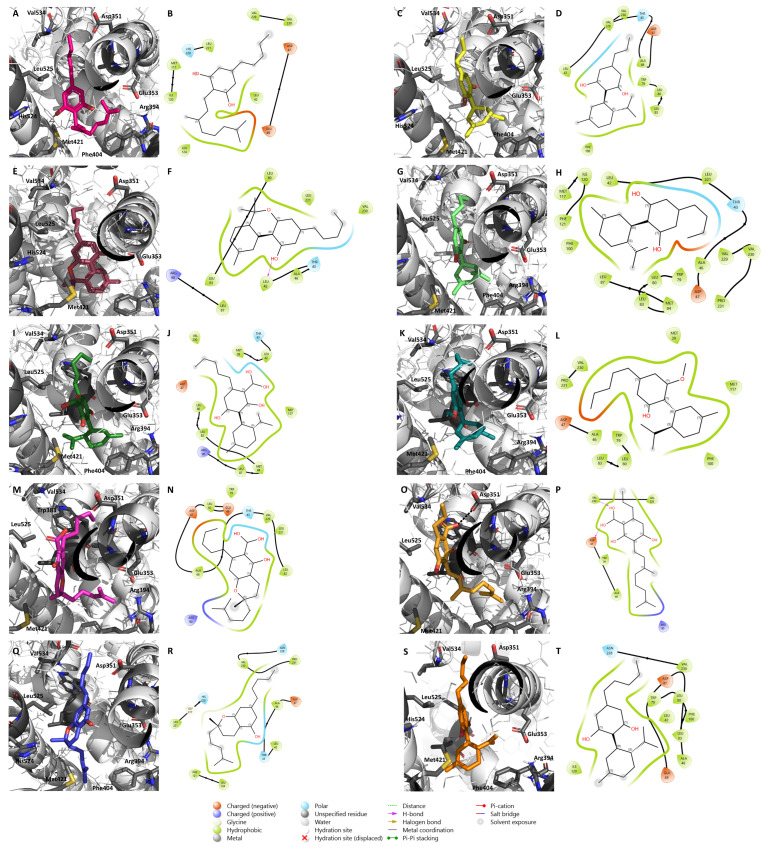
Molecular docking of the nine minor cannabinoids, **CBG** (**A**,**B**), **CBDV** (**C**,**D**), **CBN** (**E**,**F**), **CBDB** (**G**,**H**), **CBDA** (**I**,**J**), **CBDM** (**K**,**L**), **CBCA** (**M**,**N**), **CBGVA** (**O**,**P**), and **CBC** (**Q**,**R**), as well as **CBD** (**S**,**T**), in ERα. The images represent both 2D and 3D ERα/cannabinoid interactions. ERα structure was generated by the authors and is represented in as cartoon. The main amino acid residues of the target, including Asp351, Glu353, Arg394, Phe404, Met421, His 524, Leu525, Val534, and Trp383, are shown as gray sticks, while the cannabinoids are shown as different colorful sticks. Hydrogen bonds are represented as black dashed lines in the 3D representations and as purple arrows in the 2D maps.

The binding of these compounds to ERα is depicted in Figure 4. **CBD** (Figure 4J) was also included for molecular docking comparison, as it acts as an ER antagonist with inverse agonist properties [44]. As target flexibility is important for the ligand/ERα binding, and in order to better understand the energetic stability and conformational pattern of the ligand/target complexes, a 10 ns MD simulation was performed based on the most stable complex structures derived from the docking studies presented in Figure 4. After the MD run, all the ligands remained in the active site in locations similar to the ones presented at the beginning of the simulation (Supplementary Figure S4). In fact, the protein–ligand complexes were clearly overlapping, demonstrating the stability of the complexes and the lack of significant conformational changes related to the ligand binding. Furthermore, the MD simulations indicate that the complex structures are in a stable state as the potential energy at the end of the simulation is lower than the initial conformations, indicating more stability of the system (Supplementary Figure S4). As demonstrated by RMSD plots, all the ligands were stable after 2–6 ns with an RMSD value of around 2 Å, and the complex backbone acquired a stable trajectory beyond 2–6 ns with an RMSD value of 2.5–3 Å (Supplementary Figure S4).

**Figure 5:**
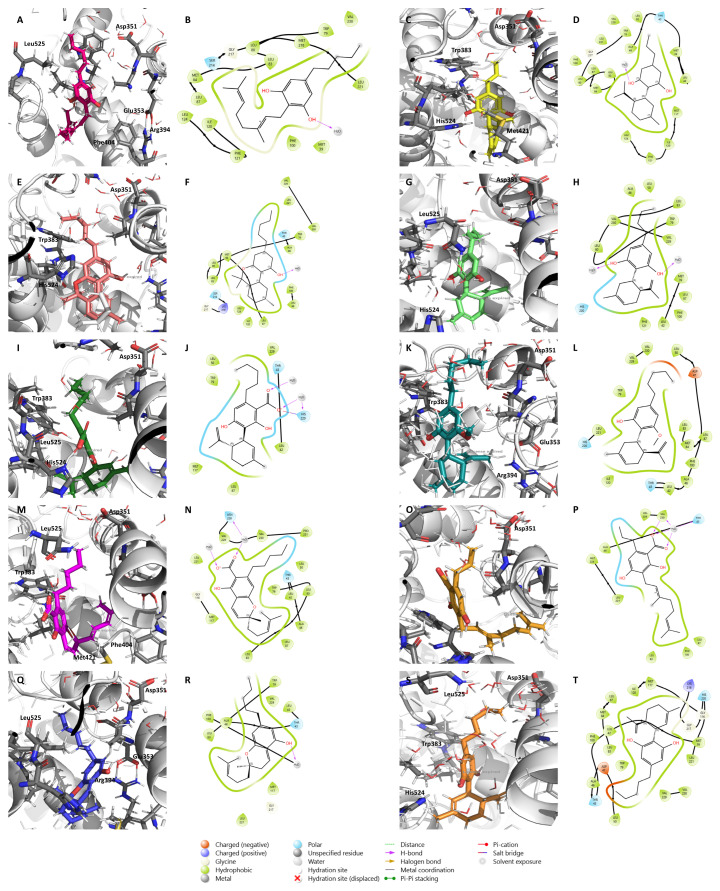
Molecular dynamics simulation on ERα for the nine minor cannabinoids, **CBG** (**A**,**B**), **CBDV** (**C**,**D**), **CBN** (**E**,**F**), **CBDB** (**G**,**H**), **CBDA** (**I**,**J**), **CBDM** (**K**,**L**), **CBCA** (**M**,**N**), **CBGVA** (**O**,**P**), and **CBC** (**Q**,**R**), as well as **CBD** (**S**,**T**). A molecular dynamics simulation was performed around the conformation of the small molecules obtained from the docking analysis on ERα. The main amino acid residues of the target are shown as gray sticks, while the cannabinoids are shown as colorful sticks.

The complete MD simulation study was carried out for 10 ns, during which the ligand lost some of the interactions established in the pre-MD docked pose (Figure 4), and established new ones, as evident from Figure 5.

The results showed that, with the exception of **CBDM** (Figure 5F), for all the other cannabinoids the complex ERα/cannabinoid acquires a position in which the cannabinoid slightly moves away from Asp351, a residue that is essential for the antagonistic activity, and gets buried in the bottom of the ERα binding site, suggesting that these compounds may not antagonize the receptor (Figure 5). However, considering the 2D maps, **CBDM** (Figure 5L) and **CBD** (Figure 5T) are complexed in the vicinity of Asp351 (referred to as Asp47 in the 2D maps), suggesting that these cannabinoids might influence the antagonistic conformation of the receptor. In fact, in vitro studies have already demonstrated that **CBD** displays antagonistic activity on ER [44]. Nevertheless, the cannabinoids were still able to establish some important interactions for their stability, namely with Phe404 and Trp383.

**Figure 6:**
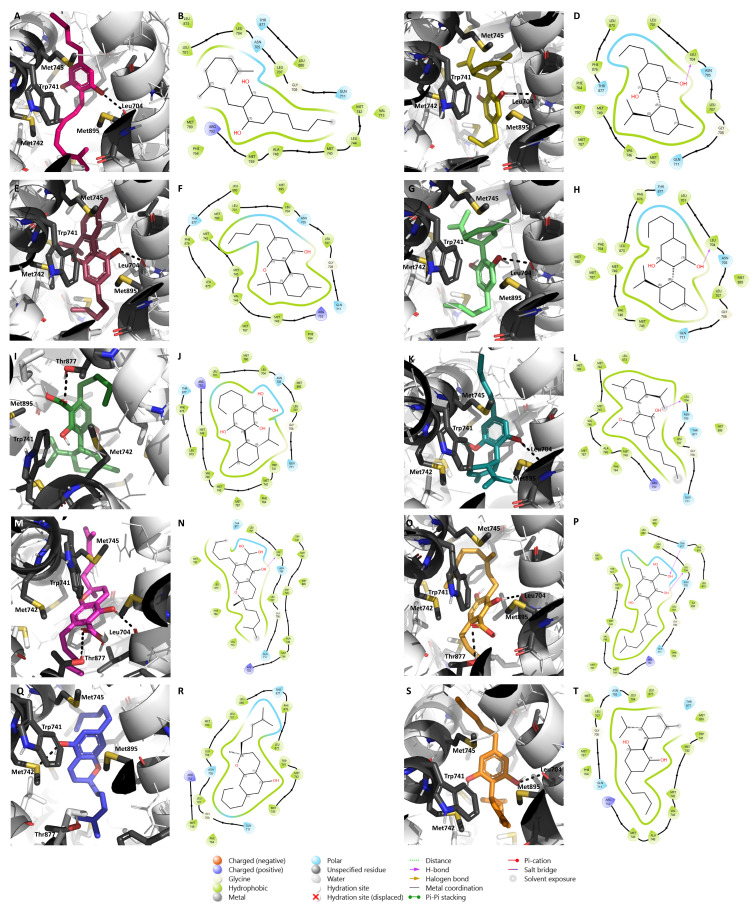
Molecular docking of the nine minor cannabinoids, **CBG** (**A**,**B**), **CBDV** (**C**,**D**), **CBN** (**E**,**F**), **CBDB** (**G**,**H**), **CBDA** (**I**,**J**), **CBDM** (**K**,**L**), **CBCA** (**M**,**N**), **CBGVA** (**O**,**P**), and **CBC** (**Q**,**R**), as well as **CBD** (**S**,**T**), in AR. The images represent both 2D and 3D AR/cannabinoid interactions. AR structure has the PDB code 2AMA and is represented in as cartoon. The main amino acid residues of the target, including Met745, Leu704, Trp741, Met895, Met742, and Thr877, are shown as gray sticks, while the cannabinoids are shown as different colorful sticks. Hydrogen bonds are represented as black dashed lines in the 3D representations and as purple arrows in the 2D maps.

AR has been receiving a lot of attention for its role in ER^+^ breast cancer development and, consequently, as a potential therapeutic target for this disease [24,86]. According to the function played by this steroid receptor in sensitive ER^+^ tumors, the compounds studied should, for therapeutic purposes, act as AR agonists [87]. Therefore, we docked the nine selected cannabinoids on an agonistic structure of this receptor and the results showed that the AR binding site appears to be small to properly accommodate these cannabinoids. Nevertheless, they established interactions, mainly hydrophobic, with several residues, including Met745, Trp741, Leu704, Met895, Met342, and Thr877 (Figure 6). Furthermore, they were stabilized by hydrogen bonds with Thr877, Leu704, and Met742. Thr877 has been known to be important for the agonist’s stabilization [88,89,90]. In fact, **CBD** docking in AR showed the lack of ability of this compound to interact with Thr877 (Figure 6J) and our previous in vitro studies have demonstrated that **CBD** acts as an AR antagonist with inverse agonist properties [44]. Following this, we postulate that **CBDA**, **CBCA**, and **CBGVA**, by establishing this bond with Thr877, may act as AR agonists, while the others may act as AR antagonists.

### 2.2. Effects of the Cannabinoids in Aromatase

Since our docking analysis suggested that the seven minor cannabinoids selected had the potential to inhibit aromatase, a radiometric assay was performed to confirm these assumptions, using human placental microsomes, an aromatase enriched matrix [26]. The anti-aromatase activity of the compounds **CBG** and **CBN**, as well as **CBD**, was also evaluated in this assay. The reference AIs used in a clinic, Ana, Let, and Exe, were used as positive controls. Our results showed that none of the minor cannabinoids significantly inhibited aromatase, 12% (**CBDV**) being the highest anti-aromatase activity value determined (Table 4). As expected, **CBD**, at 10 µM, presented an anti-aromatase activity of 83.23% (Table 4). In accordance with our previous studies [25,26,91,92,93], the reference AIs induced a high anti-aromatase activity (>98%) (Table 4).

Microsomes (20 µg) were incubated with NADPH (15 mM), each cannabinoid (10 μM), and [1β-3H]-androstenedione (40 nM), during 15 min at 37 °C. Results are presented as a percentage of tritiated water released, in relation to the control, and are represented as the mean ± SEM of three independent experiments carried out in triplicate. The reference AIs, Ana and Let, at 1 µM, were used as positive controls.

### 2.3. Effects of Cannabinoids in Estrogen Receptor (ER)

**Figure 7:**
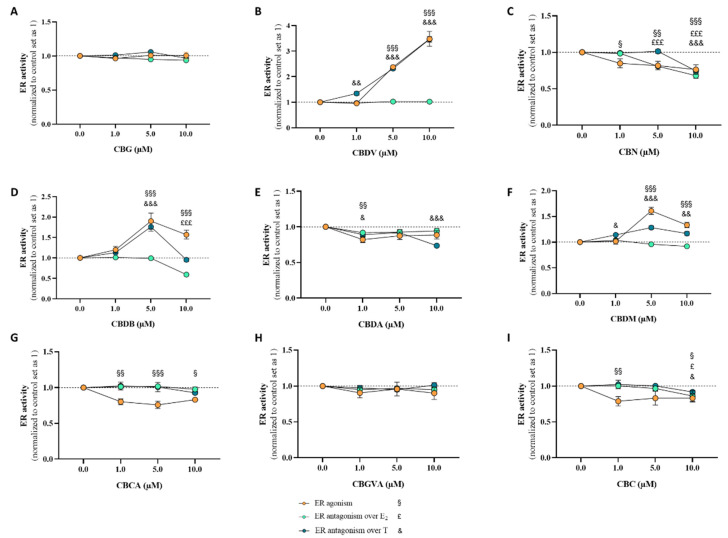
Transactivation assays for the determination of ERα agonist/antagonist activity induced by the nine minor cannabinoids, **CBG** (**A**), **CBDV** (**B**), **CBN** (**C**), **CBDB** (**D**), **CBDA** (**E**), **CBDM** (**F**), **CBCA** (**G**), **CBGVA** (**H**), and **CBC** (**I**). VM7Luc4E2 cells were treated with the cannabinoids (1, 5, and 10 μM) in the absence (ERα agonism) or presence (ERα antagonism) of testosterone (T; 1 nM) or estradiol (E_2_; 91.8 pM) for 24 h. Results are presented as the mean ± SEM of at least three independent experiments performed in triplicate. Statistically significant differences between the control (cells not exposed to cannabinoids), set as 1, and cells not treated with T or E_2_ are expressed as § (*p* < 0.05), §§ (*p* < 0.01), and §§§ (*p* < 0.001), while the differences between the control and cells treated with cannabinoids in the presence of E_2_ are expressed as £ (*p* < 0.05) and £££ (*p* < 0.001), and the differences between cells treated with cannabinoids in the presence of T are expressed as & (*p* < 0.05), && (*p* < 0.01), and &&& (*p* < 0.001).

For the evaluation of the effects of cannabinoids on ER activity, the VM7Luc4E2 cell line was treated for 24 h with each phytocannabinoid (1–10 μM) in the presence (ER antagonism) or absence (ER agonism) of E_2_ (91.8 pM) or T (1 nM). Our results (Figure 7) demonstrated that, in the absence of E_2_ or T, **CBN** (*p* < 0.05, *p* < 0.01, *p* < 0.001; Figure 7C), **CBDA** (*p* < 0.01; Figure 7E), **CBCA** (*p* < 0.05; *p* < 0.01; *p* < 0.001; Figure 7G), and **CBC** (*p* < 0.05; *p* < 0.01; Figure 7I) presented a negative effect for agonism, whereas **CBDV** (*p* < 0.001; Figure 7B), **CBDB** (*p* < 0.001; Figure 7D), and **CBDM** (*p* < 0.001; Figure 7F) presented a positive effect and thus exhibited ER agonist activity. Of note, **CBG** (Figure 7A) and **CBGVA** (Figure 7H) did not exert any significant effect on the activity of this receptor. A reduction in VM7Luc4E2 cell viability for these conditions was observed for **CBDV** (*p* < 0.01; Supplementary Figure S5B), **CBN** (*p* < 0.001; Supplementary Figure S5C), **CBDB** (*p* < 0.01; *p* < 0.001; Supplementary Figure S5D), and **CBDM** (*p* < 0.05; *p* < 0.01; Supplementary Figure S5F). In the presence of E_2_, for ER antagonism evaluation, a significant decrease in relation to the control value was only observed for 5 and 10 μM of **CBN** (*p* < 0.001; Figure 7C), and for **CBDB** (*p* < 0.001; Figure 7D), and **CBC** (*p* < 0.05; Figure 7I), both at 10 μM, suggesting that these compounds act as ER antagonists. For the remaining cannabinoids under study, no significant differences were observed (Figure 7). However, it is important to consider that at these conditions, contrary to **CBG** and **CBGVA**, a significant reduction (*p* < 0.05; *p* < 0.01; *p* < 0.001; Supplementary Figure S5) in cell viability was detected, which may influence the transactivation results. When the antagonism was addressed in the presence of T instead of E_2_, different behaviors were observed. Regarding **CBDV** (*p* < 0.01; *p* < 0.001; Figure 7B), **CBDB** (*p* < 0.001; Figure 7D), and **CBDM** (*p* < 0.05; *p* < 0.01; *p* < 0.001; Figure 7F), no effects were noted for the antagonism over T. On the other hand, **CBN** at 10 μM (*p* < 0.001; Figure 7C), **CBDA** at 1 and 10 μM (*p* < 0.05; *p* < 0.001; Figure 7E), and **CBC** at 10 μM (*p* < 0.05; Figure 7I) acted as ER antagonists under these conditions. No significant effects were detected for **CBG** (Figure 7A), **CBCA** (Figure 7G), and **CBGVA** (Figure 7H). Nevertheless, **CBDV** (*p* < 0.05; *p* < 0.001; Supplementary Figure S5B), **CBN** (*p* < 0.001; Supplementary Figure S5C), **CBDB** (*p* < 0.01; *p* < 0.001; Supplementary Figure S5D), **CBDM** (*p* < 0.05; *p* < 0.01; *p* < 0.001; Supplementary Figure S5F), and **CBC** (*p* < 0.05; *p* < 0.001; Supplementary Figure S5I) reduced cell viability under these conditions. As previously reported by our group, **CBD** acts as an ER antagonist, with inverse agonist properties [44].

### 2.4. Effects of Cannabinoids in Androgen Receptor (AR)

**Figure 8:**
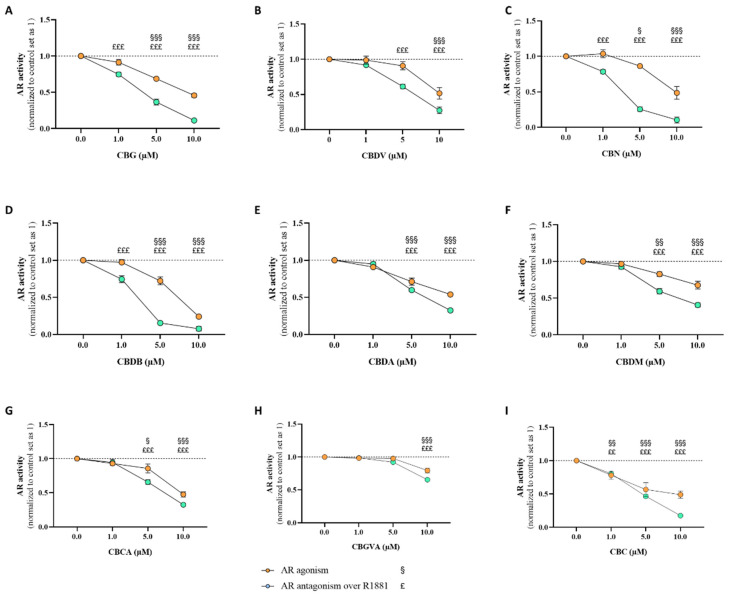
Transactivation assays for the determination of AR agonist/antagonist activity induced by the nine minor cannabinoids, **CBG** (**A**), **CBDV** (**B**), **CBN** (**C**), **CBDB** (**D**), **CBDA** (**E**), **CBDM** (**F**), **CBCA** (**G**), **CBGVA** (**H**), and **CBC** (**I**). CHO-K1 cells were treated with the cannabinoids (1, 5, and 10 μM) in the absence (AR agonism) or presence (AR antagonism) of R1881 (0.1 nM) for 24 h. Results are presented as the mean ± SEM of at least three independent experiments performed in triplicate. Statistically significant differences between the control (cells not exposed to cannabinoids), set as 1, and cells not treated with R1881 are expressed as § (*p* < 0.05), §§ (*p* < 0.01), and §§§ (*p* < 0.001), while the differences between the control and cells treated with cannabinoids in the presence of R1881 are expressed as ££ (*p* < 0.01) and £££ (*p* < 0.001).

The activity of the nine minor cannabinoids towards AR was assessed through the AR-EcoScreen^TM^ assay. The cells were treated with the cannabinoids (1–10 μM) for 24 h in the presence (antagonism) or absence (agonism) of R1881 (0.1 nM). The results demonstrated that all the studied cannabinoids caused no AR agonism (*p* < 0.05; *p* < 0.01; *p* < 0.001; Figure 8). On the contrary, all the nine cannabinoids exerted AR antagonistic activity, suggesting that they act as AR antagonists (*p* < 0.01; *p* < 0.001; Figure 8). **CBG** (*p* < 0.001; Figure 8A), **CBN** (*p* < 0.001; Figure 8C), **CBDB** (*p* < 0.001; Figure 8D), and **CBC** (*p* < 0.01; *p* < 0.001; Figure 8I) antagonistic activities were verified for all the concentrations studied, while for **CBDV** (*p* < 0.001; Figure 8B), **CBDA** (*p* < 0.001; Figure 8E), **CBDM** (*p* < 0.001; Figure 8F), and **CBCA** (*p* < 0.001; Figure 8G), the AR antagonistic activity was only observed at 5 and 10 μM and for **CBGVA** (*p* < 0.001; Figure 8H), only at 10 μM. In addition, contrary to **CBGVA** for which an increase in the luciferase activity signal was verified at 10 μM (*p* < 0.01; *p* < 0.001), which might be due to cell proliferation, all the other treatments slightly reduced cell viability (*p* < 0.05; *p* < 0.001), mainly at 10 μM (Supplementary Figure S6). As previously reported by our group, **CBD** acts as an AR antagonist, with inverse agonist properties [44].

### 2.5. Scores and Ranking of the Cannabinoids Using ToxPi

**Figure 9:**
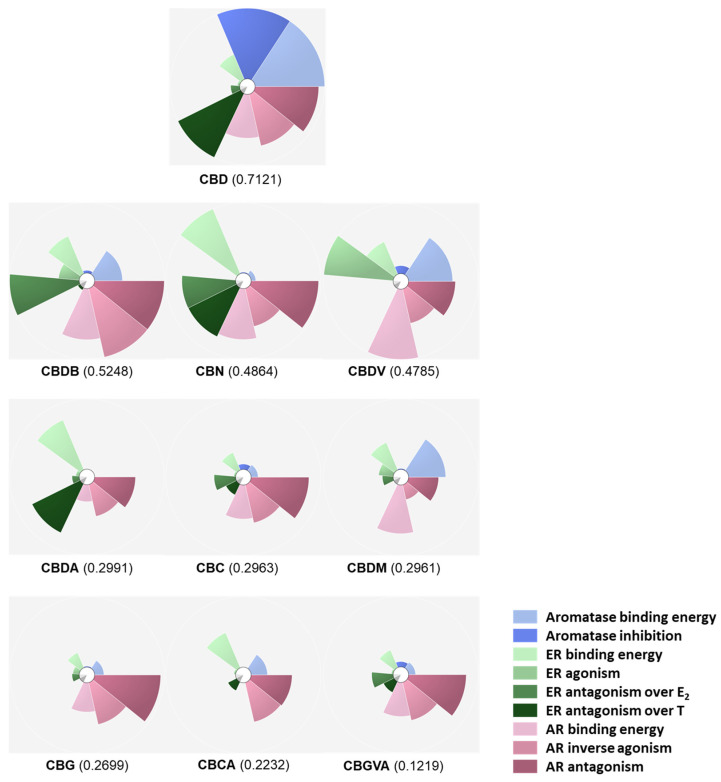
ToxPi scores and ranks. The information regarding the docking analysis in aromatase, ER, and AR, as well as the information about aromatase inhibition and ER and AR agonistic/antagonistic activities for the nine selected cannabinoids and **CBD**, was inserted in different slices in ToxPi and used to rank the compounds.

The data regarding the scores of the docking in aromatase, ER, and AR, as well as aromatase inhibition and ER and AR agonistic/antagonistic activities of the nine studied cannabinoids and **CBD** were added to ToxPi and used to score and rank the compounds overall. As expected, **CBD** obtained the best ToxPi score (0.7121) mainly due to its aromatase inhibition results, score of the docking in aromatase, ER antagonistic activity over T, and AR inverse agonistic and antagonistic activities. Regarding the minor phytocannabinoids, **CBDB** scored the best (0.5248) because of the high values determined for ER antagonistic activity over E_2_ and AR docking score, as well as AR inverse agonistic and antagonistic activities. **CBN** ranks in third place with a ToxPi score of 0.4864 for which ER and AR docking scores, ER antagonism over E_2_ and T, and AR antagonistic activity contributed the most. The scores for the remaining minor cannabinoids were mainly determined by AR modulation, except for **CBDA,** in which ER modulation contributed significantly, and for **CBDM**, where the aromatase binding score was also relevant. The cannabinoid with the worst ToxPi score was **CBGVA** (0.1219). The overall rank and ToxPi scores are presented in Figure 9.

## 3. Discussion

The use of endocrine therapies, such as AIs and anti-estrogens, for ER^+^ breast cancer treatment has been facing concerns and limitations mainly due to endocrine resistance development, which has led to a search for their combination with other innovative therapies. However, these therapies also cause resistance [13,94,95,96], which is the reason why the discovery and development of novel therapeutic approaches are necessary. Various studies on the anti-tumor effects of cannabinoids in different cancer forms have been performed, showing promising results. Indeed, our group has demonstrated that **THC** and **CBD** decrease aromatase and ERα protein levels in ER^+^ breast cancer cells and that **CBD** can also inhibit aromatase [42] and presents ER and AR antagonistic, as well as inverse agonist, properties, in both receptors [44]. Other studies reported that **THC** presents anti-proliferative effects in ER^+^ breast cancer cells independent of ER and AR [63], although having the ability to impair ERα signaling by up-regulating ERβ [55]. Moreover, it was already described that **THC** and **CBD** exert no ER agonistic activity [62]. Considering all this, the ability of the known 129 phytocannabinoids to modulate key targets associated with ER^+^ breast cancer progression was investigated in this study.

Due to its fundamental role in local estrogen production and tumor development, aromatase was the first target studied. Our in silico studies predicted that 45 out of the 129 phytocannabinoids may act as AIs. Besides the binding affinity score, one of the reasons for their selection was the interactions that they, in theory, manage to establish with this enzyme, such as a hydrogen bond with the Asp309 residue, considered fundamental for the aromatization reaction [84,85]. In fact, the AIs used in the clinic do bind to this key residue, thereby presenting high anti-aromatase activity [83,97,98]. ERα, a key target that through estrogen binding promotes the signal transduction necessary for tumor growth and survival, was also explored. In this case, 85 cannabinoids out of the 129 showed important binding features that led us to consider them as potential ERα antagonists. One of the most important features taken into consideration was their ability to be localized in the vicinity of helix 12, a flexible helix of ERα that, upon antagonist binding, prevents the binding of coactivators, compromising receptor activation [99]. On the other hand, their possible proximity and establishment of hydrogen bonds with Asp351, a residue close to helix 12 and to which antagonists typically bind, were also taken into account [6]. Considering these results, it is possible to suggest that, theoretically, cannabinoids have a higher potential to bind and act as ERα antagonists than as AIs. Furthermore, 36 compounds out of the 45 potential AIs and of the 85 potential ER antagonists were simultaneously considered as both promising AIs as well as ERα antagonists, suggesting that they may act as multi-target compounds in ER^+^ breast cancer. In fact, the importance of multi-target compounds has been pointed out by several authors [6,13,91,100,101,102,103,104], since by having the ability to simultaneously modulate different targets, they may exhibit fewer side effects. Additionally, our group has previously verified that aromatase, ERα, and ERβ binding sites present some similarities, reinforcing the possible application of multi-target compounds in this disease [6].

However, for in vitro studies, we focused our attention on only 7 out of the 36 cannabinoids, **CBDV**, **CBDB**, **CBDM**, **CBDA**, **CBCA**, **CBGVA**, and **CBC**, as the remaining were not commercially available. Additionally, despite the absence of in silico evidence of a relevant modulation of aromatase and ERα, **CBG** and **CBN** were included in our study, as some studies in different tumors, including triple-negative breast cancer, have pointed out their therapeutic potential as anti-cancer compounds [75,76]. Regarding aromatase activity, we confirmed that **CBG** and **CBN** do not inhibit this enzyme. However, none of the other seven cannabinoids were able to greatly inhibit aromatase (only up to 12% inhibition). **CBD**, as expected and corroborating our previous work [65], presented high anti-aromatase activity. In fact, our previous molecular docking studies suggested the binding of **CBD** to key aromatase residues [65] and herein, **CBD** displayed the best docking score (−8.7 kcal/mol), followed by **CBDV** (−7.6 kcal/mol), which was found to inhibit aromatase by 12% at 10 μM. For aromatase inhibition, among the investigated cannabinoids, the presence of a carboxylic acid seems detrimental for activity (comparing, for example, **CBG** with **CBGVA**) and slight modifications in the hydrophobic tail of the cannabidiol type of metabolites influence the affinity for the enzyme, as evidenced when comparing **CBDV** (C_3_) with its homologues **CBDB** (C_4_) and **CBDM** (C_5_).

In relation to ERα, different results were obtained for the nine studied phytocannabinoids. According to our transactivation results, cannabidiol derivatives **CBDV**, **CBDB**, and **CBDM** are ERα agonists, with **CBDV** and **CBDB** presenting full agonist activity properties (i.e., 75-fold increase over basal response [105]). The ER agonist activity verified for these cannabinoids is not in line with previous studies that, based on **THC** and **CBD** activities, indicated that cannabinoids do not exhibit ER agonist activity [62]. Although this agonist activity is not attractive for ER^+^ breast cancer cases, it may be clinically relevant in other pathological conditions, such as neurodegenerative disorders, liver injuries, or cardiovascular diseases, where a beneficial action should not be ruled out [106,107]. On the other hand, **CBN**, **CBDA**, **CBCA**, and **CBC** may potentially act as ER inverse agonists, which may be beneficial for ER^+^ breast cancer cases since, by presenting opposite actions to ER agonists, they prevent the tumorigenic effects of ERα. These results correlate with in silico studies in which **CBDA** (−8.3 kcal/mol) presented the highest affinity for the ER. For ER inverse agonism, cyclization, particularly chromene formation, was important for activity, as in the case of cannabichromene derivatives **CBC** and **CBCA**, and for chromane **CBN**. Moreover, except for the linear **CBGVA**, the carboxylic acid derivatives investigated (**CBCA, CBDA**) were active against this receptor.

Nevertheless, the behavior exhibited by the phytocannabinoids changes under hormone influence. Contrary to **CBDV** that loses ER agonistic activity, **CBDB** and **CBDM** become ER antagonists in the presence of E_2_. In the presence of T, **CBDA** maintains ERα antagonist activity, while in the presence of E_2_, this activity is abolished. These different behaviors can be explained by the binding affinities to ERα in the presence of the different hormones. Moreover, the fixed E_2_ concentration (91.8 pM) used for the assessment of antagonistic activity induces the maximum activation of the receptor, which might be too high to detect activity of weaker antagonistic compounds. On the other hand, antagonistic activities in the presence of T (1 nM) might be more notable due to the concentration of T used, which is not associated with maximum effects. Curiously, **CBDB** fails as an ER antagonist in the presence of T. In both conditions, **CBN** and **CBC** act as ERα antagonists with inverse agonist properties, though this effect was only observed at the highest concentration for the latter compound. As previously reported, this behavior was similar to that of **CBD** [44]. This represents a therapeutic advantage for ER^+^ breast cancer and other clinical conditions that may be treated with ERα antagonists [108]. Of all the studied cannabinoids, only the two representatives of the cannabigerol type, the biosynthetic precursor **CBGVA** and **CBG**, showed no effect on ER activity, highlighting the importance of cyclization features for ER activity.

As previously mentioned, AR is gaining significant therapeutic attention in breast cancer, with some AR-target therapies being studied in different models. In fact, AR activation can modulate other receptors, such as ER and HER2 [109]. Regarding ER^+^ breast cancer and considering the beneficial effect of AR in those cases, the use of AR agonists would benefit the treatment of this disease, in a non-resistant scenario [20,87]. Unfortunately, our results, both in silico and in vitro, revealed that the nine cannabinoids studied acted as AR antagonists with inverse agonist properties, a behavior similar to the one observed for **CBD** [44]. This result is in accordance with some other studies that have pointed out that drugs of abuse, such as marijuana, by preventing the binding of dihydrotestosterone to AR, present AR antagonist activity [110,111]. Furthermore, due to the nature of this steroid receptor, almost only steroid hormones can act as AR agonists. Nevertheless, and despite the apparently disadvantageous effects of these compounds on AR, when ER is not overexpressed or activated in tumors, AR displays pro-survival and tumorigenic roles, contributing to tumor development [24,112]. This scenario is frequently found in resistant stages, which makes the use of AR antagonists an attractive approach. Thus, our data suggest that cannabinoids, by acting as AR antagonists with inverse agonist properties, might be a beneficial therapy in resistant scenarios, or might even impair the AR oncogenic role known for ER^+^ breast cancer cells treated with Exe [24]. In fact, there are some ongoing clinical trials with AR antagonists and AIs or anti-estrogens for breast cancer treatment [113,114]. Furthermore, by acting as AR antagonists, the studied cannabinoids may also be attractive for other pathological conditions where AR antagonists could be clinically applied, such as prostate cancer and benign prostatic hyperplasia [115,116]. Moreover, some triple-negative breast tumors show dependence on AR signaling for growth and survival [117]. In fact, two AR antagonists, Bicalutamide and Enzalutamide, were already evaluated in clinical trials (NCT00468715, NCT01889238) [118,119,120]. Therefore, the use of the minor phytocannabinoids evaluated in this study may also be beneficial for the management of this breast cancer subtype.

By using ToxPi metrics, we confirmed the previous assumption that **CBD** is the best cannabinoid when the simultaneous action towards aromatase, ER, and AR is considered, which highlights its therapeutic potential for ER^+^ breast cancer. The best minor phytocannabinoid is **CBDB**, a **CBD** derivative, mainly due to its great ER antagonistic activity over E_2_ and its AR inverse agonistic and antagonistic activities. Similarly, the second best minor phytocannabinoid is **CBN**, whose score is also a result of its good scores on ER and AR molecular docking, but also on ER and AR antagonistic activities. Thus, considering that the effects exhibited by **CBD** on aromatase and ER surpass the ones induced on AR, which was not verified for the other cannabinoids, we can conclude that the scores attributed to the minor cannabinoids are mainly determined by the modulation of AR. Moreover, as formerly mentioned, ER antagonism is a crucial therapeutic action to be considered for ER^+^ breast cancer treatment, reason why we postulate that **CBDB** and **CBN** might be the most promising minor phytocannabinoids for hormone-dependent breast cancer treatment, along with the major phytocannabinoid **CBD**. Carboxylic acid derivatives, such as **CBGVA** and **CBCA**, occupy the last positions, indicating that this structural function is not beneficial overall. In the future, the study of these compounds in different breast cancer and non-cancer models will be of paramount importance to fully address the therapeutic potential of cannabinoids in breast cancer and guide their eventual application in the clinic.

## 4. Materials and Methods

### 4.1. Molecular Docking

A molecular docking analysis was performed to investigate the binding of 129 phytocannabinoids to aromatase, ERα, and AR. Considering mainly their resolution value, length, and absence of mutations, and the complexed ligand, the PDB structures 3S79 and 2AMA were chosen for aromatase [121] and AR [122] studies, respectively. In addition, a molecular docking was performed for each target using the ligands already reported for them, as inhibitors, antagonists, and natural substrates, as well as the respective decoys in order to validate the docking program (data not presented in the manuscript). In relation to aromatase structure and considering different studies suggesting that the Asp309 residue, crucial for the aromatization reaction, should be protonated [84,123], this residue, as well as Asp371, were considered neutral, as previously described [91]. Regarding ERα structure, the model used was generated using different and complementary structures, as previously described [91]. The structures of the compounds were either collected from ChEMBL [124] or designed using Marvin (ChemAxon, Budapest, Hungary). Their charges and configurations were further corrected using AutodockTools [125] and the molecular docking was performed using PyRx 0.8 software [126]. For each molecular target, a grid box with no more than 30 × 30 × 30 (x, y, z) dimensions was placed in the ligand binding site, covering all the important residues and considering the position of the original ligand. A maximum of nine poses for each compound was generated and the chosen ones were selected taking into account the score value and the interaction between the compounds and the targets. Additionally, 2D and 3D maps representing the interactions between the targets and the cannabinoids were generated in Maestro 5.9 (Schrödinger Release 2024-3: Maestro, Schrödinger, LLC, New York, NY, USA, 2024) and PyMol 2.5.7 (Schrödinger, LLC, New York, NY, USA, 2020), respectively.

### 4.2. Molecular Dynamics Simulation

For molecular dynamics (MD) simulation, a 5Å spherical droplet containing 100 water molecules was placed surrounding the conformation of the small molecules obtained from the docking study on ERα; the complexes were energy-minimized using the MMFF94x force field until an RMS gradient < 0.1 kcal/Å/mol. The ligand/macromolecule complexes were then subjected to MD simulation using MOE-dynamic implemented in MOE 2014.09 (Chemical Computing Groups, Montreal, QC, Canada). MD simulation was performed by choosing the MMFF94x force field and NVT (N, total atom; V, volume; T, temperature) ensemble and Nosé–Poincaré–Andersen (NPA) algorithm, with a 0.002 ps time step and sampling every 0.5 ps. The system was heated from 0 K to 300 K in 100 ps (heat stage), followed by 10,000 ps of a production stage at 300 K; the system was then cooled back to 0 K in 100 ps (cooling stage). The potential energy of the system and the root mean square deviation (RMSD) of the ligand were used to keep track of the system’s behavior during the simulation. Simulation observation was performed by examining the ligand/macromolecule complex interaction between ligand atoms and target atoms at the end of the simulation. This analysis was carried out for the nine cannabinoids selected, as well as for **CBD**.

### 4.3. Anti-Aromatase Activity

Considering the molecular docking results, the anti-aromatase activity of the nine selected minor cannabinoids, cannabigerol (**CBG**), cannabidivarin (**CBDV**), cannabinol (**CBN**), cannabidiol-C4 (**CBDB**), cannabidiolic acid (**CBDA**), cannabidiol monomethyl ether (**CBDM**), cannabichromenic acid (**CBCA**), cannabigerovarinic acid (**CBGVA**), and cannabichromene (**CBC**; Figure 2), was assessed through a radiometric assay by measuring the tritiated water released from [1*β*-^3^H]-androstenedione (PerkinElmer Life Sciences, Boston, MA, USA), using human placental microsomes, according to Thompson and Siiteri [127] and Heidrich et al. [128] modified methods [26]. This radiometric assay is the one recommended by OCSPP Guideline 890.1200 for aromatase activity assessment [129]. Despite the previously reported anti-aromatase activity of **CBD** [65], it was also assessed here at the same concentration used for the minor cannabinoids for comparison purposes. Each compound was diluted in a 67 mM potassium phosphate buffer (pH 7.4) and the aromatization reaction was performed using 20 μg of microsomal protein, 15 μM of NADPH, 40 nM of [1*β*-^3^H]-androstenedione, and 10 μM of each phytocannabinoid in a final reaction volume of 500 μL at 37 °C, during 15 min, as previously described [26]. Then, the samples were transferred to scintillation tubes containing a liquid scintillation cocktail (ICN Radiochemicals, Irvine, CA, USA), and scintillations were then counted in a liquid scintillation counter (LKB Wallac 1209 Rackbeta, LKB Wallac, Turku, Finland). The tritiated water released from [1*β*-^3^H]-androstenedione was used as an index of estrogen formation [26].

Stock solutions of each cannabinoid were prepared in 100% DMSO and stored at −20 °C. All the experiments were performed in triplicate in at least three independent experiments. Ana, Let, and Exe at 1 µM were used as reference AIs.

### 4.4. ER and AR Transactivation Assays

Considering the molecular docking results, the activity of the nine selected cannabinoids towards human ER and AR was assessed as previously described [44,100], and according to the guidelines from The Organization for Economic Cooperation and Development (OECD) for Testing of Chemicals, Tests No. 455 and 458, respectively. These bioassays are based on stably transfected mammalian cell lines and validated for a reliable detection of human ER and AR agonists and antagonists. Regarding the ER assay, we used the VM7Luc4E2 cells, which were kindly provided by Michael Denison (University of California, USA). These cells express both human ERα and ERβ isoforms and were cultured in DMEM without phenol red, supplemented with 4.5% CFBS, 1% penicillin/streptomycin, 2% L-glutamine, and 110 mg/mL sodium pyruvate for 3 days before the beginning of the experiments, and then plated at a 4 × 10^5^ cells/mL cell density in white 96-well plates. After adhesion, cells were treated with the cannabinoids (1–10 μM) for 24 h, in the absence (ER agonism) or presence of 91.8 pM 17β-estradiol (E_2_; ER antagonism) or 1 nM testosterone (T; ER antagonism). The agonistic and antagonistic activities were measured using the Steady-Glo^®^ Luciferase Assay System (Promega Corporation, Madison, WI, USA) in a multimode plate reader (EnSpire^®^, Perkin Elmer, Inc., Waltham, MA, USA). Cell viability was assessed with the CellTiter-Glo^®^ Luminescent Cell Viability Assay (Promega Corporation, Madison, WI, USA).

For AR activity assessment, the AR-EcoScreen^TM^ cell line (#JCRB1328, from Japanese Collection of Research Bioresources Cell Bank, Tokyo, Japan) applied was based on Chinese hamster ovary (CHO-K1) cells expressing the human AR with a firefly luciferase reporter construct inserted and a renilla luciferase gene for the simultaneous evaluation of viability. Cells were seeded at a density of 9 × 10^4^ cells/mL in a DMEM/F12 medium without phenol red, containing 5% CFBS and 1% penicillin/streptomycin, in white 96-well plates. After adhesion, cells were exposed to the cannabinoids (1–10 μM) for 24 h, in the absence (AR agonism) or presence of 0.1 nM methyltrienolone (R1881; AbMole BioScience, Houston, TX, USA) for investigating AR antagonism. Both AR activity and cell viability were assessed using the Dual-Glo^®^ Luciferase Assay System (Promega Corporation, Madison, WI, USA) in a multimode plate reader (EnSpire^®^, Perkin Elmer, Inc., Waltham, MA, USA).

At least three independent experiments were conducted in triplicate and the results are presented as fold change compared to a control (cells not exposed to cannabinoids), which was set as 1.

As experimental controls, T (781.2 pM–25.6 μM) and E_2_ (180 fM–367 nM) were tested as positive controls for ER agonism, while Raloxifene (12.0 pM–24.5 nM; Biosynth Ltd., Berkshire, UK) was used as a positive control for ER antagonism. Regarding AR assays, R1881 (7.8 pM–1 nM) and hydroxyflutamide (OHF; 4.1 nM–9 μM; Sigma Aldrich Co., St. Louis, MO, USA) were used as positive controls for AR agonism and antagonism, respectively. Stock solutions of all the cannabinoids, as well as T, E_2_, Raloxifen, R1881, and OHF, were prepared in 100% DMSO and stored at −20 °C. Before each experiment, fresh dilutions were prepared in a medium and the final concentration of DMSO in an exposure medium was 0.06%.

### 4.5. ToxPi Construction

Data from the nine selected cannabinoids, as well as **CBD**, on aromatase, ER, and AR were used in ToxPi packages to predict the most promising molecules. ToxPi scores and rankings were achieved through the analysis previously published [130]. Here, each slice of the ToxPi is composed of one assay with the respective results, namely, molecular docking in aromatase, aromatase inhibition, molecular docking in ER, ER agonism, ER antagonism over E_2_, ER antagonism over T, molecular docking in AR, AR agonism, and AR antagonism. Scores and rankings were generated considering the score values obtained through a molecular docking analysis and through the in vitro studies performed for the three targets.

### 4.6. Statistical Analysis

The statistical analysis was performed with GraphPad Prism 8.0.1^®^ software (GraphPad Software, Inc., San Diego, CA, USA) and by an analysis of variance (ANOVA), followed by Bonferroni and Tukey post hoc tests for multiple comparisons (two-way ANOVA and one-way ANOVA, respectively). Values of *p* < 0.05 were considered statistically significant. All the data were expressed as the mean ± standard error of the mean (SEM).

## 5. Conclusions

Through in silico and in vitro assays, this study presents a comprehensive analysis of the potential therapeutic effects of the cannabinoids, described so far, for the treatment of hormone-dependent breast tumors. These compounds showed ability to modulate key targets responsible for tumor development, such as aromatase, ER, and AR, and the integrated activities implemented on them may together represent an advantage for treatment at different stages of the disease. In fact, the nine minor cannabinoids studied showed, in vitro, potential to act either as ER antagonists with inverse agonist properties, or as ER agonists. Moreover, despite the lack of significant aromatase inhibition, all cannabinoids may act as AR antagonists with inverse agonist action. Regarding their chemical characters, key features for ER antagonism like the chromene ring or detrimental substituents for aromatase inhibition, such as the carboxylic acid, were identified and can guide the selection of further investigations on minor cannabinoids. Moreover, by elucidating their molecular targets and mechanisms of action, this study opens up a prospective application of these compounds in other cancers and clinical conditions. From the best of our knowledge, this is the first study exploring the molecular targets of minor cannabinoids and, together with previous studies, it reinforces the importance and therapeutic potential of cannabinoids in breast cancer, paving the way for novel and alternative therapeutic approaches and highlighting the medicinal potential of *Cannabis*.

## Figures and Tables

**Table 1:** Phytocannabinoids presenting AI profiles. The information obtained from molecular docking of the 45 cannabinoids considered as promising AIs, such as the bind affinity score and the description of the most important interactions, is presented. The information regarding **CBG** and **CBN** is also presented.

Cannabinoid Name	Cannabinoid Class	Binding Affinity (kcal/mol)	Relevant Interactions
8-Oxo-(-)-Δ^9^-*trans*-tetrahydrocannabinol	Δ^9^-THC	−9.2	Hydrogen bonds with Arg115 and Leu477; hydrophobic interactions with Trp224 and Phe221
11-Acetoxy-(-)-Δ^9^-*trans*-tetrahydrocannabinolic acid A	Δ^9^-THC	−8.3	Hydrogen bonds with Arg115, Met374, Asp309, and Leu477; hydrophobic interactions with Phe221
(-)-Δ^9^-*trans*-Tetrahydrocannabivarinic acid	Δ^9^-THC	−7.1	Hydrogen bonds with the heme group, Leu372, and Leu477; hydrophobic interactions with Trp224 and Phe221
(-)-Δ^9^-*trans*-Tetrahydrocannabinolic acid A-C_4_	Δ^9^-THC	−6.7	Hydrogen bonds with the heme group, Leu372, Met374, and Leu477; hydrophobic interactions with Trp224 and Phe221
(-)-Δ^9^-*trans*-Tetrahydrocannabinolic acid B	Δ^9^-THC	−6.0	Hydrogen bonds with Asp309 and Leu477; hydrophobic interactions with Trp224 and Phe221
Δ^8^-*trans*-Tetrahydrocannabinolic acid A	Δ^9^-THC	−4.4	Hydrogen bond with Asp309; hydrophobic interactions with Phe221
(-)-Δ^9^-*trans*-Tetrahydrocannabinal	Δ^9^-THC	−3.8	Hydrogen bond with Asp309; hydrophobic interactions with Trp224 and Phe221
Cannabidiol (CBD)	Cannabidiol	−8.7	Hydrogen bond with Asp309; hydrophobic interactions with Phe221
Cannabidiorcol	Cannabidiol	−7.8	Hydrogen bonds with Asp309 and Leu477; hydrophobic interactions with Phe221
(-)-Cannabidivarin (CBDV)	Cannabidiol	−7.6	Hydrogen bonds with Asp309 and Leu477; hydrophobic interactions with Phe221
Cannabidiol monomethylether (CBDM)	Cannabidiol	−7.3	Hydrogen bonds with Asp309 and Ser478; hydrophobic interactions with Phe221
Cannabidiol-C_4_ (CBDB)	Cannabidiol	−6.9	Hydrogen bonds with Asp309 and Leu477; hydrophobic interactions with Phe221
Cannabidivarinic acid	Cannabidiol	−6.6	Hydrogen bonds with Asp309 and Leu477; hydrophobic interactions with Phe221
Cannabidiolic acid (CBDA)	Cannabidiol	−5.7	Hydrogen bonds with Asp309 and Leu477; hydrophobic interactions with Phe221
Cannabigerol (CBG)	Cannabigerol	−6.1	Hydrophobic interactions with Phe221 and Trp224
Cannabinerolic acid	Cannabigerol	−6.7	Hydrogen bond with Asp309; hydrophobic interactions with Trp224 and Phe221
Camagerol	Cannabigerol	−6.6	Hydrogen bonds with Asp309, Arg115, and Met374
(-)-6,7-*cis*-Epoxycannabigerolic acid	Cannabigerol	−6.3	Hydrogen bonds with Asp309, Leu477, and Arg115; hydrophobic interactions with Trp224
Cannabigerovarinic acid (CBGVA)	Cannabigerol	−5.8	Hydrogen bond with Asp309
5-Acetyl-4-hydroxy cannabigerol	Cannabigerol	−5.1	Hydrogen bond with Asp309; hydrophobic interactions with Phe221
(-)-6,7-*trans*-Epoxycannabigerolic acid	Cannabigerol	−5.4	Hydrogen bonds with Asp309 and Leu477; hydrophobic interactions with Trp224
Cannabielsoic acid B-C_3_	Cannabielsoin	−6.0	Hydrogen bonds with the heme group and Asp309
Cannabielsoin acid A	Cannabielsoin	−5.9	Hydrogen bonds with Asp309 and Arg115
Cannabielsoin acid B	Cannabielsoin	−5.1	Hydrogen bond with Asp309; hydrophobic interactions with Trp224 and Phe221
Cannabicyclol	Cannabicyclol	−9.8	Hydrogen bond with Asp309; hydrophobic interactions with Phe221
Cannabicyclovarin	Cannabicyclol	−9.3	Hydrogen bond with Asp309; hydrophobic interactions with Phe221
Cannabicyclolic acid	Cannabicyclol	−5.5	Hydrogen bond with Leu477
Cannabichromevarin	Cannabichromene	−7.1	Hydrogen bond with Asp309
Cannabichromenic acid (CBCA)	Cannabichromene	−6.4	Hydrogen bond with Asp309; hydrophobic interactions with Trp224
Cannabichromene (CBC)	Cannabichromene	−6.0	Hydrogen bond with Asp309; hydrophobic interactions with Phe221
(-)-7-Hydroxycannabichromene	Cannabichromene	−5.5	Hydrogen bonds with the heme group and Asp309; hydrophobic interactions with Phe221
Cannabichromevarinic acid	Cannabichromene	−5.2	Hydrogen bond with Arg115
Cannabinol (CBN)	Cannabinol	−5.9	Hydrogen bond with Leu477; hydrophobic interactions with Phe221 and Trp224
8-Hydroxycannabinol	Cannabinol	−7.9	Hydrogen bonds with Asp309 and Arg115
Cannabiorcol-C_1_	Cannabinol	−6.6	Hydrogen bond with Asp309; hydrophobic interactions with Trp224 and Phe221
8-Hydroxycannabinolic acid A	Cannabinol	−4.8	Hydrogen bonds with Asp309 and Arg115; hydrophobic interactions with Trp224 and Phe221
(-)-*trans*-Cannabitriol-OEt-C_5_ *	Cannabitriol	−3.9	Hydrogen bonds with Asp309, Arg115, and Leu477; hydrophobic interactions with Phe221
(-)-*trans*-Cannabitriol-OEt-C_3_ *	Cannabitriol	−3.5	Hydrogen bonds with Asp309 and Ser478; hydrophobic interactions with Phe221
(-)-Δ^7^-*trans*-(1R,3R,6R)-isotetrahydrocannabinol-C_5_	Miscellaneous	−9.8	Hydrogen bonds with the heme group and Asp309; hydrophobic interactions with Trp224 and Phe221
Cannabimovone	Miscellaneous	−8.4	Hydrogen bond with Asp309; hydrophobic interactions with Phe221
Cannabichromanone C	Miscellaneous	−7.8	Hydrogen bonds with Asp309 and Arg115; hydrophobic interactions with Phe221
Dehydrocannabifuran	Miscellaneous	−7.3	Hydrogen bond with Asp309; hydrophobic interactions with Phe221
Cannabichromanone B	Miscellaneous	−7.0	Hydrogen bonds with the heme group and Asp309; hydrophobic interactions with Phe221
(-)-(7R)-Cannabicoumarononic acid	Miscellaneous	−6.5	Hydrogen bonds with Asp309 and Met374; hydrophobic interactions with Trp224 and Phe221
8-Hydroxy-isohexahydrocannabivarin	Miscellaneous	−6.0	Hydrogen bonds with the heme group and Asp309; hydrophobic interactions with Trp224 and Phe221
Cannabicoumaronome-C_5_	Miscellaneous	−6.0	Hydrogen bonds with the heme group and Asp309; hydrophobic interactions with Phe221
Cannabichromanone-C_3_	Miscellaneous	−5.4	Hydrogen bonds with the heme group and Asp309; hydrophobic interactions with Phe221

* Et = ethyl.

**Table 2:** Phytocannabinoids presenting ERα antagonist profiles. The information obtained from molecular docking for the 85 cannabinoids considered as promising ERα antagonists, such as the bind affinity score and the description of the most important interactions, is presented. The information regarding **CBG** and **CBN** is also presented.

Cannabinoid Name	Cannabinoid Class	Binding Affinity (kcal/mol)	Relevant Interactions
(-)-Δ^9^-*trans*-Tetrahydrocannabihexol	Δ^9^-THC	−8.5	Close to Asp351; hydrophobic interactions with Trp383
α-Terpenyl (-)-Δ^9^-*trans*- tetrahydrocannabinolate	Δ^9^-THC	−8.0	Close to Asp351; hydrophobic interactions with Phe404
11-Acetoxy-(-)-Δ^9^-*trans*-tetrahydrocannabinolic acid A	Δ^9^-THC	−7.8	Hydrogen bonds with Glu353 and Arg394; close to Asp351; hydrophobic interactions with Phe404
8-oxo-(-)-Δ^9^-*trans*-Tetrahydrocannabinol	Δ^9^-THC	−7.8	Close to Asp351; hydrophobic interactions with Trp383
(-)-Δ^9^-*trans*-Tetrahydrocannabiphorol	Δ^9^-THC	−7.8	Close to Asp351; hydrophobic interactions with Trp383
4-Terpenyl (-)-Δ^9^-*trans*-tetrahydrocannabinolate	Δ^9^-THC	−7.3	Hydrogen bond with Asp351; hydrophobic interactions with Trp383
Δ^9^-Tetrahydrocannabinol (THC)	Δ^9^-THC	−7.1	Close to Asp351; hydrophobic interactions with Trp383
(-)-Δ^9^-*trans*-Tetrahydrocannabinol-C_4_	Δ^9^-THC	−7.0	Close to Asp351; hydrophobic interactions with Trp383
(-)-Δ^9^-*trans*-Tetrahydrocannabinal	Δ^9^-THC	−6.5	Hydrogen bond with Asp351; hydrophobic interactions with Trp383
(-)-Δ^9^-*trans*-Tetrahydrocannabinolic acid A-C_4_	Δ^9^-THC	−5.4	Hydrogen bonds with Val534 and Asp351; hydrophobic interactions with Trp383
(-)-Δ^8^-*trans*-Tetrahydrocannabinol	Δ^8^-THC	−8.4	Close to Asp351; hydrophobic interactions with Trp383
10β-Hydroxy-Δ^8^-tetrahydrocannabinol	Δ^8^-THC	−8.1	Hydrogen bond with Glu353; close to Asp351; hydrophobic interactions with Trp383
10α-Hydroxy-Δ^8^-tetrahydrocannabinol	Δ^8^-THC	−7.4	Close to Asp351; hydrophobic interactions with Trp383
10a-α-Hydroxy-*o*-oxo-Δ^8^-tetrahydrocannabinol	Δ^8^-THC	−6.4	Close to Asp351; hydrophobic interactions with Trp383
Cannabidiolic acid	Cannabidiol	−8.3	Close to Asp351; hydrophobic interactions with Trp383
Cannabidiol-C_4_	Cannabidiol	−7.8	Close to Asp351; hydrophobic interactions with Trp383
(-)-Cannabidivarin	Cannabidiol	−7.6	Close to Asp351; hydrophobic interactions with Trp383
Cannabidiol monomethyl ether	Cannabidiol	−7.4	Close to Asp351; hydrophobic interactions with Trp383
Cannabidiol (CBD)	Cannabidiol	−7.3	Close to Asp351; hydrophobic interactions with Trp383
Cannabidihexol	Cannabidiol	−6.8	Close to Asp351; hydrophobic interactions with Trp383
Cannabigerol	Cannabigerol	−6.9	Possible hydrogen bond with His 524; close to Asp351
ᵞ-Eudesmyl-cannabigerolate	Cannabigerol	−7.7	Close to Asp351; hydrophobic interactions with Trp383
Cannabinerolic acid	Cannabigerol	−7.7	Hydrogen bonds with His524 and Glu353; close to Asp351; hydrophobic interactions with Trp383
5-Acetyl-4-hydroxy-cannabigerol	Cannabigerol	−7.0	Hydrogen bonds with His524 and Glu353; close to Asp351; hydrophobic interactions with Trp383 and Phe404
(-)-6,7-*trans*-Epoxycannabigerolic acid	Cannabigerol	−6.9	Hydrogen bonds with His524 and Ala350; close to Asp351; hydrophobic interactions with Trp383 and Phe404
(-)-6,7-*cis*-Epoxycannabigerolic acid	Cannabigerol	−6.9	Hydrogen bonds with His524 and Ala350; close to Asp351; hydrophobic interactions with Trp383 and Phe404
Monomethyl ether of (E)-cannabigerol	Cannabigerol	−6.8	Close to Asp351; hydrophobic interactions with Trp383
Sesquicannabigerol	Cannabigerol	−6.5	Hydrogen bonds with Asp351, Asn532, and Val534; hydrophobic interactions with Trp383
(-)-6,7-*trans*-Epoxycannabigerol	Cannabigerol	−6.4	Close to Asp351; hydrophobic interactions with Trp383
Cannabigerolic acid	Cannabigerol	−6.3	Close to Asp351; hydrophobic interactions with Trp383
Cannabigerovarinic acid	Cannabigerol	−6.3	Hydrogen bonds with Asp351 and Val534; hydrophobic interactions with Trp383
Camagerol	Cannabigerol	−6.0	Hydrogen bonds with Asp351 and Asn532
Monomethyl ether of cannabigerolic acid	Cannabigerol	−5.9	Close to Asp351; hydrophobic interactions with Trp383
(-)-6,7-*cis*-Epoxycannabigerol	Cannabigerol	−5.1	Close to Asp351; hydrophobic interactions with Trp383
Cannabinodiol	Cannabinodiol	−8.1	Close to Asp351; hydrophobic interactions with Trp383 and Phe404
Cannabinodivirin	Cannabinodiol	−7.8	Close to Asp351; hydrophobic interactions with Trp383 and Phe404
Cannabielsoin	Cannabielsoin	−8.1	Close to Asp351; hydrophobic interactions with Trp383
Cannabielsoin acid B	Cannabielsoin	−6.0	Hydrogen bond with His524; close to Asp351; hydrophobic interactions with Phe404
Cannabielsoin acid A	Cannabielsoin	−5.2	Hydrogen bonds with His524 and Ala350; close to Asp351; hydrophobic interactions with Trp383
Cannabicyclol	Cannabicyclol	−8.9	Close to Asp351; hydrophobic interactions with Trp383
Cannabicyclolic acid	Cannabicyclol	−8.7	Hydrogen bond with His524; hydrophobic interactions with Trp383
Cannabicyclovarin	Cannabicyclol	−7.8	Close to Asp351; hydrophobic interactions with Trp383
(-)-7-Hydroxycannabichromene	Cannabichromene	−8.5	Close to Asp351; hydrophobic interactions with Phe404
( ± )-4-Acetoxycannabichromene	Cannabichromene	−7.8	Hydrogen bond with Met421; close to Asp351; hydrophobic interactions with Trp383 and Phe404
Cannabichromenic acid	Cannabichromene	−7.7	Close to Asp351; hydrophobic interactions with Trp383
2-Methyl-2-(4-methyl-2-pentyl)-7-propyl-2H-1-benzopyran-5-ol	Cannabichromene	−7.6	Close to Asp351; hydrophobic interactions with Phe404
Cannabichromevarin	Cannabichromene	−7.6	Close to Asp351; hydrophobic interactions with Phe404
Cannabichromevarinic acid	Cannabichromene	−7.6	Hydrogen bond with Asp351; hydrophobic interactions with Trp383
(-)-3′′-Hydroxy-Δ^4′′^-cannabichromene	Cannabichromene	−7.2	Close to Asp351; hydrophobic interactions with Trp383
Cannabichromene	Cannabichromene	−7.0	Close to Asp351; hydrophobic interactions with Trp383
Cannabinol	Cannabinol	−8.9	Hydrophobic interactions with Trp383 and Phe404
Cannabinol methyl ether	Cannabinol	−8.4	Close to Asp351; hydrophobic interactions with Trp383 and Phe404
Cannabinol-C_4_	Cannabinol	−6.6	Close to Asp351; hydrophobic interactions with Trp383 and Phe404
(10S)-Hydroxycannabinol	Cannabinol	−6.4	Hydrogen bond with Val534; close to Asp351; hydrophobic interactions with Trp383 and Phe404
8-Hydroxycannabinol	Cannabinol	−6.0	Close to Asp351; hydrophobic interactions with Trp383 and Phe404
8-Hydroxycannabinolic acid A	Cannabinol	−5.4	Hydrogen bond with Val524; hydrophobic interaction with Phe404
(+)-*trans*-Cannabitriol-C3	Cannabitriol	−7.7	Close to Asp351; hydrophobic interactions with Trp383
(+)-*cis*-Cannabitriol-C_5_	Cannabitriol	−7.6	Close to Asp351; hydrophobic interactions with Trp383
(-)-*cis*-Cannabitriol-C_5_	Cannabitriol	−7.6	Close to Asp351; hydrophobic interactions with Trp383
(+)-*trans*-Cannabitriol-C_5_	Cannabitriol	−7.5	Close to Asp351; hydrophobic interactions with Trp383
(-)-*trans*-Cannabitriol-OEt-C_3_ *	Cannabitriol	−7.5	Hydrogen bond with His524; close to Asp351; hydrophobic interactions with Trp383
(-)-*trans*-Cannabitriol-C_5_	Cannabitriol	−6.8	Hydrogen bond with Met421; close to Asp351; hydrophobic interactions with Trp383
Cannabitriol	Cannabitriol	−6.5	Close to Asp351; hydrophobic interactions with Trp383
Cannabioxepane	Miscellaneous	−9.2	Close to Asp351; hydrophobic interactions with Trp383 and Phe404
(-)-Δ^9^-*cis*-(6aS,10aR)-Tetrahydrocannabinol	Miscellaneous	−8.7	Close to Asp351; hydrophobic interactions with Trp383
Dehydrocannabifuran	Miscellaneous	−8.5	Close to Asp351; hydrophobic interactions with Trp383 and Phe404
Cannabifuran	Miscellaneous	−8.1	Close to Asp351; hydrophobic interactions with Trp383 and Phe404
10α-Hydroxyhexahydrocannabinol	Miscellaneous	−8.0	Close to Asp351; hydrophobic interactions with Trp383
2-Geranyl-5-hydroxy-3-n-pentyl-1,4-benzoquinone	Miscellaneous	−7.9	Close to Asp351; hydrophobic interactions with Trp383
9α-Hydroxyhexahydrocannabinol	Miscellaneous	−7.9	Close to Asp351; hydrophobic interactions with Trp383
10-Oxo-Δ^6a(10a)^-tetrahydrocannabinol	Miscellaneous	−7.7	Close to Asp351; hydrophobic interactions with Trp383
Cannabichromanone D	Miscellaneous	−7.7	Close to Asp351; hydrophobic interactions with Trp383
Cannabichromanone-C_3_	Miscellaneous	−7.5	Hydrogen bond with His524; close to Asp351; hydrophobic interactions with Trp383
Cannabicoumaronome-C_5_	Miscellaneous	−7.2	Close to Asp351; hydrophobic interactions with Trp383
Cannabimovone	Miscellaneous	−7.2	Close to Asp351; hydrophobic interactions with Trp383
9β, 10β-Epoxyhexahydrocannabinol	Miscellaneous	−7.0	Close to Asp351; hydrophobic interactions with Trp383
10α-Hydroxy-Δ^9,11^-hexahydrocannabinol	Miscellaneous	−7.0	Close to Asp351; hydrophobic interactions with Trp383
10αR-Hydroxyhexahydrocannabinol	Miscellaneous	−6.7	Close to Asp351; hydrophobic interactions with Trp383
Cannabichromanone C	Miscellaneous	−6.7	Hydrogen bond with Val534; close to Asp351; hydrophobic interactions with Phe404
(-)-Δ^7^-*trans*-(1R,3R,6R)-Isotetrahydrocannabinol-C_5_	Miscellaneous	−6.6	Hydrogen bond with His524; close to Asp351
9α-Hydroxy-10-oxo-Δ^6a,10a^-tetrahydrocannabinol	Miscellaneous	−6.6	Close to Asp351; hydrophobic interactions with Trp383
4-Acetoxy-2-geranyl-5-hydroxy-3-n-pentylphenol	Miscellaneous	−6.4	Close to Asp351; hydrophobic interactions with Phe404
7-Oxo-9α-hydroxyhexahydrocannabinol	Miscellaneous	−6.4	Close to Asp351; hydrophobic interactions with Trp383
(-)-Cannabitetrol	Miscellaneous	−6.4	Close to Asp351; hydrophobic interactions with Trp383
(-)-(7R)-Cannabicoumaronic acid	Miscellaneous	−6.4	Close to Asp351; hydrophobic interactions with Trp383
Cannabichromanone B	Miscellaneous	−6.4	Close to Asp351; hydrophobic interactions with Phe404
(-)-Cannabiripsol-C_5_	Miscellaneous	−6.2	Close to Asp351; hydrophobic interactions with Trp383

* Et = ethyl.

**Table 3:** Phytocannabinoids presenting dual functions: AI and ERα antagonist profiles. Information about the binding affinity score of the 36 cannabinoids on aromatase and ERα is presented. The information regarding **CBG** and **CBN** is also presented.

Cannabinoid Name	Cannabinoid Class	Binding Affinity—Aromatase (kcal/mol)	Binding Affinity—ERα (kcak/mol)
8-Oxo-(-)-Δ^9^-*trans*-tetrahydrocannabinol	Δ^9^-THC	−9.2	−7.8
11-Acetoxy-(-)-Δ^9^-*trans*-tetrahydrocannabinolic acid A	Δ^9^-THC	−8.3	−7.8
(-)-Δ^9^-*trans*-Tetrahydrocannabinolic acid A-C_4_	Δ^9^-THC	−6.7	−5.4
(-)-Δ^9^-*trans*-Tetrahydrocannabinal	Δ^9^-THC	−3.8	−6.5
Cannabidiol (CBD)	Cannabidiol	−8.7	−7.3
(-)-Cannabidivarin (CBDV)	Cannabidiol	−7.6	−7.6
Cannabidiol monomethylether (CBDM)	Cannabidiol	−7.3	−7.4
Cannabidiol-C_4_ (CBDB)	Cannabidiol	−6.9	−7.8
Cannabidiolic acid (CBDA)	Cannabidiol	−5.7	−8.3
Cannabinerolic acid	Cannabigerol	−6.7	−7.7
Camagerol	Cannabigerol	−6.6	−6.0
(-)-6,7-*cis*-Epoxycannabigerolic acid	Cannabigerol	−6.3	−6.9
Cannabigerol (CBG)	Cannabigerol	−6.1	−6.9
Cannabigerovarinic acid (CBGVA)	Cannabigerol	−5.8	−6.3
(-)-6,7-*trans*-Epoxycannabigerolic acid	Cannabigerol	−5.4	−6.9
5-Acetyl-4-hydroxy-cannabigerol	Cannabigerol	−5.1	−7.0
Cannabielsoin acid A	Cannabielsoin	−5.9	−5.2
Cannabielsoin acid B	Cannabielsoin	−5.1	−6.0
Cannabicyclol	Cannabicyclol	−9.8	−8.9
Cannabicyclovarin	Cannabicyclol	−9.3	−7.8
Cannabicyclolic acid	Cannabicyclol	−5.5	−8.7
Cannabichromevarin	Cannabichromene	−7.1	−7.6
Cannabichromenic acid (CBCA)	Cannabichromene	−6.4	−7.7
Cannabichromene (CBC)	Cannabichromene	−6.0	−7.0
(-)-7-Hydroxycannabichromene	Cannabichromene	−5.5	−8.5
Cannabichromevarinic acid	Cannabichromene	−5.2	−7.6
8-Hydroxycannabinol	Cannabinol	−7.9	−6.0
Cannabinol (CBN)	Cannabinol	−5.9	−8.9
8-Hydroxycannabinolic acid A	Cannabinol	−4.8	−5.4
(-)-*trans*-Cannabitriol-OEt-C_3_ *	Cannabitriol	−3.5	−7.5
(-)-Δ^7^-*trans*-(1R,3R,6R)-isotetrahydrocannabinol-C_5_	Miscellaneous	−9.8	−6.6
Cannabimovone	Miscellaneous	−8.4	−7.2
Cannabichromanone C	Miscellaneous	−7.8	−6.7
Dehydrocannabifuran	Miscellaneous	−7.3	−8.5
Cannabichromanone B	Miscellaneous	−7.0	−6.4
(-)-(7R)-Cannabicoumarononic acid	Miscellaneous	−6.5	−6.4
Cannabicoumaronome-C_5_	Miscellaneous	−6.0	−7.2
Cannabichromanone-C_3_	Miscellaneous	−5.4	−7.5

* Et = ethyl.

**Table 4:** Percentages of anti-aromatase activity obtained for the cannabinoids under study.

Cannabinoid	Aromatase Inhibition (%) ± SEM	Binding Affinity—Aromatase (kcal/mol)
**CBC**	8.9 ± 1.7	−6.0
**CBCA**	3.0 ± 0.9	−6.4
**CBD**	83.2 ± 0.6	−8.7
**CBDA**	3.4 ± 1.3	−5.7
**CBDB**	6.4 ± 3.6	−6.9
**CBDM**	4.5 ± 2.8	−7.3
**CBDV**	12.0 ± 2.5	−7.6
**CBG**	3.6 ± 2.3	−6.1
**CBGVA**	7.8 ± 0.9	−5.8
**CBN**	3.8 ± 0.5	−5.9
**Ana**	98.8 ± 0.2	−8.2
**Let**	99.4 ± 0.2	−7.3
**Exe**	98.4 ± 0.3	−8.2

## Data Availability

The original contributions presented in the study are included in the article/Supplementary Material, further inquiries can be directed to the corresponding author.

## Supplementary Materials

The following supporting information can be downloaded at: https://www.mdpi.com/article/10.3390/ph17091245/s1, Figure S1. Chemical structures of the cannabinoids described in Table 1. Figure S2. Chemical structures of the cannabinoids described in Table 2. Figure S3. Chemical structures of the cannabinoids described in Table 3. Figure S4. Molecular dynamics (MD) of ERα complexed with CBC (A), CBCA (B), CBD (C), CBDA (D), CBDB (E), CBDM (F), CBDV (G), CBG (H), CBGVA (I), and CBN (J). Results at the beginning (t = 0 ps, green) and at the end (t = 10,200 ps, blue) of the simulation (left), RMSD plot (center) and Potential Energy plot (right) during the time of simulation. Figure S5. Effects of the nine minor cannabinoids, CBG (A), CBDV (B), CBN (C), CBDB (D), CBDA (E), CBDM (F), CBCA (G), CBGVA (H) and CBC (I), on VM7LucE2 cell viability. Cells were treated with the cannabinoids (1, 5 and 10 μM) in the absence (ERα agonism) or presence (ERα antagonism) of testosterone (T; 1 nM) or estradiol (E2; 91.8 pM) for 24h. Results are presented as mean ± SEM of at least three independent experiments performed in triplicate. Statistically sig-nificant differences between control (cells not exposed to cannabinoids), set as 1, and cells not treated with T or E2 are expressed as * (*p* < 0.05), ** (*p* < 0.01) and *** (*p* < 0.001), while the differences between control and cells treated with cannabinoids in the presence of E2 are expressed as $ (*p* <0.05), $$ (*p* < 0.01) and $$$ (*p* < 0.001), and the differences between control cells and cells treated with cannabinoids in the presence of T are expressed as # (*p* < 0.05), ## (*p* < 0.01) and ### (*p* < 0.001). Figure S6. Effects of the nine minor cannabinoids, CBG (A), CBDV (B), CBN (C), CBDB (D), CBDA (E), CBDM (F), CBCA (G), CBGVA (H) and CBC (I), on CHO-K1 cells viability. Cells were treated with the cannabinoids (1, 5 and 10 μM) in the absence (AR agonism) or presence (AR an-tagonism) of R1881 (0.1 nM) for 24h. Results are presented as mean ± SEM of at least three inde-pendent experiments performed in triplicate. Statistically significant differences between control (cells not exposed to cannabinoids), set as 1, and cells not treated with R1881 are expressed as * (*p* < 0.05), ** (*p* < 0.01) and *** (*p* < 0.001), while the differences between control and cells treated with cannabinoids in the presence of R1881 are expressed as $$$ (*p* < 0.001).

## Abbreviations

Δ^9^-THCA: Δ^9^-tetrahydrocannabinolic acid; Δ^9^-THCV: Δ^9^-tetrahydrocannabivarin; Δ^9^-THCVA: Δ^9^-tetrahydrocannabivaric acid; 17β-estradiol: E_2_; AIs: aromatase inhibitors; Ana: anastrozole; AR: androgen receptor; CBC: cannabichromene; CBCA: cannabichromenic acid; CBD: cannabidiol; CBDA: cannabidiolic acid; CBDB: cannabidiol-C4; CBDM: cannabidiol monomethyl ether; CBDV: cannabidivarin; CBDVA: cannabidivarinic acid; CBG: cannabigerol; CBGA: cannabigerolic acid; CBGVA: cannabigerovarinic acid; CBN: cannabinol; CBV: cannabivarin; ER: estrogen receptor; ER^+^: estrogen receptor-positive; Exe: exemestane; GPP: geranyl diphosphate; HER2^+^: human epidermal growth factor receptor 2-positive; Let: letrozole; MD: molecular dynamics; T: testosterone; THC: Δ^9^-tetrahydrocannabinol.

## References

[1] Sung H., Ferlay J., Siegel R.L., Laversanne M., Soerjomataram I., Jemal A., Bray F. (2021). Global cancer statistics 2020: GLOBOCAN estimates of incidence and mortality worldwide for 36 cancers in 185 countries. CA Cancer J. Clin..

[2] Siegel R.L., Miller K.D., Wagle N.S., Jemal A. (2023). Cancer statistics, 2023. CA Cancer J. Clin..

[3] Society A.C. (2019). Breast Cancer Facts & Figures 2019–2020.

[4] Muller K., Jorns J.M., Tozbikian G. (2022). What’s new in breast pathology 2022: WHO 5th edition and biomarker updates. J. Pathol. Transl. Med..

[5] Johnson K.S., Conant E.F., Soo M.S. (2021). Molecular Subtypes of Breast Cancer: A Review for Breast Radiologists. J. Breast Imaging.

[6] Ferreira Almeida C., Oliveira A., João Ramos M., Fernandes P.A., Teixeira N., Amaral C. (2020). Estrogen receptor-positive (ER^+^) breast cancer treatment: Are multi-target compounds the next promising approach?. Biochem. Pharmacol..

[7] Ghosh D. (2023). Aromatase and steroid sulfatase from human placenta. Methods Enzymol..

[8] Awan A., Esfahani K. (2018). Endocrine therapy for breast cancer in the primary care setting. Curr. Oncol..

[9] Cardoso F., Kyriakides S., Ohno S., Penault-Llorca F., Poortmans P., Rubio I.T., Zackrisson S., Senkus E. (2019). Early breast cancer: ESMO Clinical Practice Guidelines for diagnosis, treatment and follow-up†. Ann. Oncol..

[10] Paluch-Shimon S., Cardoso F., Partridge A.H., Abulkhair O., Azim H.A., Bianchi-Micheli G., Cardoso M.J., Curigliano G., Gelmon K.A., Gentilini O. (2022). ESO-ESMO fifth international consensus guidelines for breast cancer in young women (BCY5). Ann. Oncol..

[11] Gennari A., André F., Barrios C.H., Cortés J., de Azambuja E., DeMichele A., Dent R., Fenlon D., Gligorov J., Hurvitz S.A. (2021). ESMO Clinical Practice Guideline for the diagnosis, staging and treatment of patients with metastatic breast cancer. Ann. Oncol..

[12] Cardoso F., Paluch-Shimon S., Schumacher-Wulf E., Matos L., Gelmon K., Aapro M.S., Bajpai J., Barrios C.H., Bergh J., Bergsten-Nordström E. (2024). 6th and 7th International consensus guidelines for the management of advanced breast cancer (ABC guidelines 6 and 7). Breast.

[13] Augusto T.V., Correia-da-Silva G., Rodrigues C.M.P., Teixeira N., Amaral C. (2018). Acquired resistance to aromatase inhibitors: Where we stand!. Endocr. Relat. Cancer.

[14] Saatci O., Huynh-Dam K.T., Sahin O. (2021). Endocrine resistance in breast cancer: From molecular mechanisms to therapeutic strategies. J. Mol. Med..

[15] Olson E. (2018). Combination Therapies in Advanced, Hormone Receptor-Positive Breast Cancer. J. Adv. Pract. Oncol..

[16] Roberto M., Astone A., Botticelli A., Carbognin L., Cassano A., D’Auria G., Fabbri A., Fabi A., Gamucci T., Krasniqi E. (2021). CDK4/6 Inhibitor Treatments in Patients with Hormone Receptor Positive, Her2 Negative Advanced Breast Cancer: Potential Molecular Mechanisms, Clinical Implications and Future Perspectives. Cancers.

[17] André F., Ciruelos E., Rubovszky G., Campone M., Loibl S., Rugo H.S., Iwata H., Conte P., Mayer I.A., Kaufman B. (2019). Alpelisib for PIK3CA-Mutated, Hormone Receptor-Positive Advanced Breast Cancer. N. Engl. J. Med..

[18] Kolyvas E.A., Caldas C., Kelly K., Ahmad S.S. (2022). Androgen receptor function and targeted therapeutics across breast cancer subtypes. Breast Cancer Res..

[19] García X., Elía A., Galizzi L., May M., Spengler E., Martínez Vázquez P., Burruchaga J., Gass H., Lanari C., Lamb C.A. (2020). Increased androgen receptor expression in estrogen receptor-positive/progesterone receptor-negative breast cancer. Breast Cancer Res. Treat..

[20] Michmerhuizen A.R., Spratt D.E., Pierce L.J., Speers C.W. (2020). ARe we there yet? Understanding androgen receptor signaling in breast cancer. NPJ Breast Cancer.

[21] Hanamura T., Hayashi S.I. (2018). Overcoming aromatase inhibitor resistance in breast cancer: Possible mechanisms and clinical applications. Breast Cancer.

[22] Rechoum Y., Rovito D., Iacopetta D., Barone I., Andò S., Weigel N.L., O’Malley B.W., Brown P.H., Fuqua S.A. (2014). AR collaborates with ERα in aromatase inhibitor-resistant breast cancer. Breast Cancer Res. Treat..

[23] Basile D., Cinausero M., Iacono D., Pelizzari G., Bonotto M., Vitale M.G., Gerratana L., Puglisi F. (2017). Androgen receptor in estrogen receptor positive breast cancer: Beyond expression. Cancer Treat. Rev..

[24] Amaral C., Augusto T.V., Almada M., Cunha S.C., Correia-da-Silva G., Teixeira N. (2020). The potential clinical benefit of targeting androgen receptor (AR) in estrogen-receptor positive breast cancer cells treated with Exemestane. Biochim. Biophys. Acta Mol. Basis Dis..

[25] Augusto T.V., Cunha S.C., Amaral C., Fernandes J.O., da Silva E.T., Roleira F.F.M., Teixeira N., Correia-da-Silva G. (2019). A novel GC-MS methodology to evaluate aromatase activity in human placental microsomes: A comparative study with the standard radiometric assay. Anal. Bioanal. Chem..

[26] Roleira F.M.F., Varela C., Amaral C., Costa S.C., Correia-da-Silva G., Moraca F., Costa G., Alcaro S., Teixeira N.A.A., Tavares da Silva E.J. (2019). C-6α- vs. C-7α-Substituted Steroidal Aromatase Inhibitors: Which Is Better? Synthesis, Biochemical Evaluation, Docking Studies, and Structure-Activity Relationships. J. Med. Chem..

[27] Klumpers L.E., Thacker D.L. (2019). A Brief Background on Cannabis: From Plant to Medical Indications. J. AOAC Int..

[28] Alves P., Amaral C., Teixeira N., Correia-da-Silva G. (2020). Cannabis sativa: Much more beyond Δ9-tetrahydrocannabinol. Pharmacol. Res..

[29] Pertwee R.G. (2006). Cannabinoid pharmacology: The first 66 years. Br. J. Pharmacol..

[30] Grimaldi C., Capasso A. (2011). The endocannabinoid system in the cancer therapy: An overview. Curr. Med. Chem..

[31] Velasco G., Sánchez C., Guzmán M. (2012). Towards the use of cannabinoids as antitumour agents. Nat. Rev. Cancer.

[32] Caffarel M.M., Andradas C., Pérez-Gómez E., Guzmán M., Sánchez C. (2012). Cannabinoids: A new hope for breast cancer therapy?. Cancer Treat. Rev..

[33] Almeida C.F., Teixeira N., Correia-da-Silva G., Amaral C. (2021). Cannabinoids in Breast Cancer: Differential Susceptibility According to Subtype. Molecules.

[34] Velasco G., Sánchez C., Guzmán M. (2016). Anticancer mechanisms of cannabinoids. Curr. Oncol..

[35] Hinz B., Ramer R. (2019). Anti-tumour actions of cannabinoids. Br. J. Pharmacol..

[36] Sledzinski P., Zeyland J., Slomski R., Nowak A. (2018). The current state and future perspectives of cannabinoids in cancer biology. Cancer Med..

[37] Fraguas-Sánchez A.I., Martín-Sabroso C., Torres-Suárez A.I. (2018). Insights into the effects of the endocannabinoid system in cancer: A review. Br. J. Pharmacol..

[38] Seltzer E.S., Watters A.K., MacKenzie D., Granat L.M., Zhang D. (2020). Cannabidiol (CBD) as a Promising Anti-Cancer Drug. Cancers.

[39] Munson A.E., Harris L.S., Friedman M.A., Dewey W.L., Carchman R.A. (1975). Antineoplastic activity of cannabinoids. J. Natl. Cancer Inst..

[40] Bifulco M., Laezza C., Pisanti S., Gazzerro P. (2006). Cannabinoids and cancer: Pros and cons of an antitumour strategy. Br. J. Pharmacol..

[41] Ramer R., Hinz B. (2017). Cannabinoids as Anticancer Drugs. Adv. Pharmacol..

[42] Amaral C., Trouille F.M., Almeida C.F., Correia-da-Silva G., Teixeira N. (2021). Unveiling the mechanism of action behind the anti-cancer properties of cannabinoids in ER^+^ breast cancer cells: Impact on aromatase and steroid receptors. J. Steroid Biochem. Mol. Biol..

[43] Mokoena D., George B.P., Abrahamse H. (2024). Cannabidiol Combination Enhances Photodynamic Therapy Effects on MCF-7 Breast Cancer Cells. Cells.

[44] Almeida C.F., Teixeira N., Valente M.J., Vinggaard A.M., Correia-da-Silva G., Amaral C. (2023). Cannabidiol as a Promising Adjuvant Therapy for Estrogen Receptor-Positive Breast Tumors: Unveiling Its Benefits with Aromatase Inhibitors. Cancers.

[45] Schoeman R., Beukes N., Frost C. (2020). Cannabinoid Combination Induces Cytoplasmic Vacuolation in MCF-7 Breast Cancer Cells. Molecules.

[46] Shrivastava A., Kuzontkoski P.M., Groopman J.E., Prasad A. (2011). Cannabidiol induces programmed cell death in breast cancer cells by coordinating the cross-talk between apoptosis and autophagy. Mol. Cancer Ther..

[47] Sultan A.S., Marie M.A., Sheweita S.A. (2018). Novel mechanism of cannabidiol-induced apoptosis in breast cancer cell lines. Breast.

[48] Suttithumsatid W., Sukketsiri W., Panichayupakaranant P. (2023). Cannabinoids and standardized cannabis extracts inhibit migration, invasion, and induce apoptosis in MCF-7 cells through FAK/MAPK/Akt/NF-κB signaling. Toxicol. In Vitro.

[49] Pino S., Espinoza L., Jara-Gutiérrez C., Villena J., Olea A.F., Díaz K. (2023). Study of Cannabis Oils Obtained from Three Varieties of C. sativa and by Two Different Extraction Methods: Phytochemical Characterization and Biological Activities. Plants.

[50] Schoeman R., de la Harpe A., Beukes N., Frost C.L. (2022). Cannabis with breast cancer treatment: Propitious or pernicious?. 3 Biotech..

[51] García-Morales L., Mendoza-Rodríguez M.G., Tapia Ramírez J., Meza I. (2023). CBD Inhibits In Vivo Development of Human Breast Cancer Tumors. Int. J. Mol. Sci..

[52] Oliveira H.A., Somvanshi R.K., Kumar U. (2023). Comparative changes in breast cancer cell proliferation and signalling following somatostatin and cannabidiol treatment. Biochem. Biophys. Res. Commun..

[53] Alsherbiny M.A., Bhuyan D.J., Low M.N., Chang D., Li C.G. (2021). Synergistic Interactions of Cannabidiol with Chemotherapeutic Drugs in MCF7 Cells: Mode of Interaction and Proteomics Analysis of Mechanisms. Int. J. Mol. Sci..

[54] García-Morales L., Castillo A.M., Tapia Ramírez J., Zamudio-Meza H., Domínguez-Robles M.D.C., Meza I. (2020). CBD Reverts the Mesenchymal Invasive Phenotype of Breast Cancer Cells Induced by the Inflammatory Cytokine IL-1β. Int. J. Mol. Sci..

[55] Takeda S., Yoshida K., Nishimura H., Harada M., Okajima S., Miyoshi H., Okamoto Y., Amamoto T., Watanabe K., Omiecinski C.J. (2013). Δ^9^-Tetrahydrocannabinol disrupts estrogen-signaling through up-regulation of estrogen receptor β (ERβ). Chem. Res. Toxicol..

[56] Takeda S., Yamaori S., Motoya E., Matsunaga T., Kimura T., Yamamoto I., Watanabe K. (2008). Δ^9^-Tetrahydrocannabinol enhances MCF-7 cell proliferation via cannabinoid receptor-independent signaling. Toxicology.

[57] Takeda S., Yamamoto I., Watanabe K. (2009). Modulation of Δ^9^-tetrahydrocannabinol-induced MCF-7 breast cancer cell growth by cyclooxygenase and aromatase. Toxicology.

[58] Morin-Buote J., Ennour-Idrissi K., Poirier É., Lemieux J., Furrer D., Burguin A., Durocher F., Diorio C. (2021). Association of Breast Tumour Expression of Cannabinoid Receptors CBR1 and CBR2 with Prognostic Factors and Survival in Breast Cancer Patients. J. Pers. Med..

[59] Kisková T., Mungenast F., Suváková M., Jäger W., Thalhammer T. (2019). Future Aspects for Cannabinoids in Breast Cancer Therapy. Int. J. Mol. Sci..

[60] Dobovišek L., Krstanović F., Borštnar S., Debeljak N. (2020). Cannabinoids and Hormone Receptor-Positive Breast Cancer Treatment. Cancers.

[61] Dobovišek L., Hojnik M., Ferk P. (2016). Overlapping molecular pathways between cannabinoid receptors type 1 and 2 and estrogens/androgens on the periphery and their involvement in the pathogenesis of common diseases (Review). Int. J. Mol. Med..

[62] Ruh M.F., Taylor J.A., Howlett A.C., Welshons W.V. (1997). Failure of cannabinoid compounds to stimulate estrogen receptors. Biochem. Pharmacol..

[63] von Bueren A.O., Schlumpf M., Lichtensteiger W. (2008). Δ^9^-Tetrahydrocannabinol inhibits 17beta-estradiol-induced proliferation and fails to activate androgen and estrogen receptors in MCF7 human breast cancer cells. Anticancer. Res..

[64] Almada M., Oliveira A., Amaral C., Fernandes P.A., Ramos M.J., Fonseca B., Correia-da-Silva G., Teixeira N. (2019). Anandamide targets aromatase: A breakthrough on human decidualization. Biochim. Biophys. Acta Mol. Cell Biol. Lipids.

[65] Almada M., Amaral C., Oliveira A., Fernandes P.A., Ramos M.J., Fonseca B.M., Correia-da-Silva G., Teixeira N. (2020). Cannabidiol (CBD) but not tetrahydrocannabinol (THC) dysregulate in vitro decidualization of human endometrial stromal cells by disruption of estrogen signaling. Reprod. Toxicol..

[66] Pertwee R.G. (2016). Handbook of Cannabis.

[67] Appendino G., Chianese G., Taglialatela-Scafati O. (2011). Cannabinoids: Occurrence and medicinal chemistry. Curr. Med. Chem..

[68] Gould J. (2015). The cannabis crop. Nature.

[69] Procaccia S., Lewitus G.M., Lipson Feder C., Shapira A., Berman P., Meiri D. (2022). Cannabis for Medical Use: Versatile Plant Rather Than a Single Drug. Front. Pharmacol..

[70] Sampson P.B. (2021). Phytocannabinoid Pharmacology: Medicinal Properties of Cannabis sativa Constituents Aside from the “Big Two”. J. Nat. Prod..

[71] Welling M.T., Deseo M.A., Bacic A., Doblin M.S. (2022). Biosynthetic origins of unusual cannabimimetic phytocannabinoids in Cannabis sativa L: A review. Phytochemistry.

[72] Nachnani R., Raup-Konsavage W.M., Vrana K.E. (2021). The Pharmacological Case for Cannabigerol. J. Pharmacol. Exp. Ther..

[73] Lah T.T., Novak M., Pena Almidon M.A., Marinelli O., Žvar Baškovič B., Majc B., Mlinar M., Bošnjak R., Breznik B., Zomer R. (2021). Cannabigerol Is a Potential Therapeutic Agent in a Novel Combined Therapy for Glioblastoma. Cells.

[74] Lah T.T., Majc B., Novak M., Sušnik A., Breznik B., Porčnik A., Bošnjak R., Sadikov A., Malavolta M., Halilčević S. (2022). The Cytotoxic Effects of Cannabidiol and Cannabigerol on Glioblastoma Stem Cells May Mostly Involve GPR55 and TRPV1 Signalling. Cancers.

[75] Ligresti A., Moriello A.S., Starowicz K., Matias I., Pisanti S., De Petrocellis L., Laezza C., Portella G., Bifulco M., Di Marzo V. (2006). Antitumor activity of plant cannabinoids with emphasis on the effect of cannabidiol on human breast carcinoma. J. Pharmacol. Exp. Ther..

[76] McAllister S.D., Christian R.T., Horowitz M.P., Garcia A., Desprez P.Y. (2007). Cannabidiol as a novel inhibitor of Id-1 gene expression in aggressive breast cancer cells. Mol. Cancer Ther..

[77] Silva-Reis R., Silva A.M.S., Oliveira P.A., Cardoso S.M. (2023). Antitumor Effects of Cannabis sativa Bioactive Compounds on Colorectal Carcinogenesis. Biomolecules.

[78] Anis O., Vinayaka A.C., Shalev N., Namdar D., Nadarajan S., Anil S.M., Cohen O., Belausov E., Ramon J., Mayzlish Gati E. (2021). Cannabis-Derived Compounds Cannabichromene and Δ9-Tetrahydrocannabinol Interact and Exhibit Cytotoxic Activity against Urothelial Cell Carcinoma Correlated with Inhibition of Cell Migration and Cytoskeleton Organization. Molecules.

[79] Nahler G. (2023). Treatment of malignant diseases with phytocannabinoids: Promising observations in animal models and patients. Explor. Med..

[80] Pagano C., Navarra G., Coppola L., Bifulco M., Laezza C. (2021). Molecular Mechanism of Cannabinoids in Cancer Progression. Int. J. Mol. Sci..

[81] Salbini M., Quarta A., Russo F., Giudetti A.M., Citti C., Cannazza G., Gigli G., Vergara D., Gaballo A. (2021). Oxidative Stress and Multi-Organel Damage Induced by Two Novel Phytocannabinoids, CBDB and CBDP, in Breast Cancer Cells. Molecules.

[82] Chan H.J., Petrossian K., Chen S. (2016). Structural and functional characterization of aromatase, estrogen receptor, and their genes in endocrine-responsive and -resistant breast cancer cells. J. Steroid Biochem. Mol. Biol..

[83] Hong Y., Rashid R., Chen S. (2011). Binding features of steroidal and nonsteroidal inhibitors. Steroids.

[84] Di Nardo G., Breitner M., Bandino A., Ghosh D., Jennings G.K., Hackett J.C., Gilardi G. (2015). Evidence for an elevated aspartate p*K_a_* in the active site of human aromatase. J. Biol. Chem..

[85] Park J., Czapla L., Amaro R.E. (2013). Molecular simulations of aromatase reveal new insights into the mechanism of ligand binding. J. Chem. Inf. Model..

[86] Cochrane D.R., Bernales S., Jacobsen B.M., Cittelly D.M., Howe E.N., D’Amato N.C., Spoelstra N.S., Edgerton S.M., Jean A., Guerrero J. (2014). Role of the androgen receptor in breast cancer and preclinical analysis of enzalutamide. Breast Cancer Res..

[87] Caswell-Jin J.L., Curtis C. (2021). Androgen receptor agonists as breast cancer therapeutics. Nat. Med..

[88] Li D., Zhou W., Pang J., Tang Q., Zhong B., Shen C., Xiao L., Hou T. (2019). A magic drug target: Androgen receptor. Med. Res. Rev..

[89] Liu N., Zhou W., Guo Y., Wang J., Fu W., Sun H., Li D., Duan M., Hou T. (2018). Molecular Dynamics Simulations Revealed the Regulation of Ligands to the Interactions between Androgen Receptor and Its Coactivator. J. Chem. Inf. Model..

[90] Sakkiah S., Kusko R., Pan B., Guo W., Ge W., Tong W., Hong H. (2018). Structural Changes Due to Antagonist Binding in Ligand Binding Pocket of Androgen Receptor Elucidated Through Molecular Dynamics Simulations. Front. Pharmacol..

[91] Almeida C.F., Teixeira N., Oliveira A., Augusto T.V., Correia-da-Silva G., Ramos M.J., Fernandes P.A., Amaral C. (2021). Discovery of a multi-target compound for estrogen receptor-positive (ER^+^) breast cancer: Involvement of aromatase and ERs. Biochimie.

[92] Varela C.L., Amaral C., Correia-da-Silva G., Costa S.C., Carvalho R.A., Costa G., Alcaro S., Teixeira N.A., Tavares-da-Silva E.J., Roleira F.M. (2016). Exploring new chemical functionalities to improve aromatase inhibition of steroids. Bioorg. Med. Chem..

[93] Roleira F.M.F., Costa S.C., Gomes A.R., Varela C.L., Amaral C., Augusto T.V., Correia-da-Silva G., Romeo I., Costa G., Alcaro S. (2023). Design, synthesis, biological activity evaluation and structure-activity relationships of new steroidal aromatase inhibitors. The case of C-ring and 7β substituted steroids. Bioorg. Chem..

[94] Rasha F., Sharma M., Pruitt K. (2021). Mechanisms of endocrine therapy resistance in breast cancer. Mol. Cell. Endocrinol..

[95] Portman N., Alexandrou S., Carson E., Wang S., Lim E., Caldon C.E. (2019). Overcoming CDK4/6 inhibitor resistance in ER-positive breast cancer. Endocr. Relat. Cancer.

[96] Papadimitriou M.C., Pazaiti A., Iliakopoulos K., Markouli M., Michalaki V., Papadimitriou C.A. (2022). Resistance to CDK4/6 inhibition: Mechanisms and strategies to overcome a therapeutic problem in the treatment of hormone receptor-positive metastatic breast cancer. Biochim. Biophys. Acta Mol. Cell Res..

[97] Hong Y., Yu B., Sherman M., Yuan Y.C., Zhou D., Chen S. (2007). Molecular basis for the aromatization reaction and exemestane-mediated irreversible inhibition of human aromatase. Mol. Endocrinol..

[98] Suvannang N., Nantasenamat C., Isarankura-Na-Ayudhya C., Prachayasittikul V. (2011). Molecular Docking of Aromatase Inhibitors. Molecules.

[99] Pavlin M., Spinello A., Pennati M., Zaffaroni N., Gobbi S., Bisi A., Colombo G., Magistrato A. (2018). A Computational Assay of Estrogen Receptor alpha Antagonists Reveals the Key Common Structural Traits of Drugs Effectively Fighting Refractory Breast Cancers. Sci. Rep..

[100] Amaral C., Correia-da-Silva G., Almeida C.F., Valente M.J., Varela C., Tavares-da-Silva E., Vinggaard A.M., Teixeira N., Roleira F.M.F. (2023). An Exemestane Derivative, Oxymestane-D1, as a New Multi-Target Steroidal Aromatase Inhibitor for Estrogen Receptor-Positive (ER^+^) Breast Cancer: Effects on Sensitive and Resistant Cell Lines. Molecules.

[101] Kucuksayan E., Ozben T. (2017). Hybrid Compounds as Multitarget Directed Anticancer Agents. Curr. Top. Med. Chem..

[102] Li X., Wu C., Lin X., Cai X., Liu L., Luo G., You Q., Xiang H. (2019). Synthesis and biological evaluation of 3-aryl-quinolin derivatives as anti-breast cancer agents targeting ERα and VEGFR-2. Eur. J. Med. Chem..

[103] Petrelli A., Giordano S. (2008). From single- to multi-target drugs in cancer therapy: When aspecificity becomes an advantage. Curr. Med. Chem..

[104] Amaral C., Varela C.L., Mauricio J., Sobral A.F., Costa S.C., Roleira F.M.F., Tavares-da-Silva E.J., Correia-da-Silva G., Teixeira N. (2017). Anti-tumor efficacy of new 7α-substituted androstanes as aromatase inhibitors in hormone-sensitive and resistant breast cancer cells. J. Steroid Biochem. Mol. Biol..

[105] Berg K.A., Clarke W.P. (2018). Making Sense of Pharmacology: Inverse Agonism and Functional Selectivity. Int. J. Neuropsychopharmacol..

[106] Paterni I., Granchi C., Katzenellenbogen J.A., Minutolo F. (2014). Estrogen receptors alpha (ERα) and beta (ERβ): Subtype-selective ligands and clinical potential. Steroids.

[107] Li J., Yu J., Zou H., Zhang J., Ren L. (2023). Estrogen receptor-mediated health benefits of phytochemicals: A review. Food Funct..

[108] Martinkovich S., Shah D., Planey S.L., Arnott J.A. (2014). Selective estrogen receptor modulators: Tissue specificity and clinical utility. Clin. Interv. Aging.

[109] Kono M., Fujii T., Lim B., Karuturi M.S., Tripathy D., Ueno N.T. (2017). Androgen Receptor Function and Androgen Receptor-Targeted Therapies in Breast Cancer: A Review. JAMA Oncol..

[110] Purohit V., Ahluwahlia B.S., Vigersky R.A. (1980). Marihuana inhibits dihydrotestosterone binding to the androgen receptor. Endocrinology.

[111] Mobisson S.K., Ikpi D.E., Wopara I., Obembe A.O., Omotuyi O. (2022). Inhibition of human androgen receptor by Δ^9^-tetrahydro-cannabinol and cannabidiol related to reproductive dysfunction: A computational study. Andrologia.

[112] Bleach R., McIlroy M. (2018). The Divergent Function of Androgen Receptor in Breast Cancer; Analysis of Steroid Mediators and Tumor Intracrinology. Front. Endocrinol..

[113] Krop I., Abramson V., Colleoni M., Traina T., Holmes F., Garcia-Estevez L., Hart L., Awada A., Zamagni C., Morris P.G. (2020). A Randomized Placebo Controlled Phase II Trial Evaluating Exemestane with or without Enzalutamide in Patients with Hormone Receptor-Positive Breast Cancer. Clin. Cancer Res..

[114] Schwartzberg L.S., Yardley D.A., Elias A.D., Patel M., LoRusso P., Burris H.A., Gucalp A., Peterson A.C., Blaney M.E., Steinberg J.L. (2017). A Phase I/Ib Study of Enzalutamide Alone and in Combination with Endocrine Therapies in Women with Advanced Breast Cancer. Clin. Cancer Res..

[115] Madersbacher S., Sampson N., Culig Z. (2019). Pathophysiology of Benign Prostatic Hyperplasia and Benign Prostatic Enlargement: A Mini-Review. Gerontology.

[116] Vickman R.E., Franco O.E., Moline D.C., Vander Griend D.J., Thumbikat P., Hayward S.W. (2020). The role of the androgen receptor in prostate development and benign prostatic hyperplasia: A review. Asian J. Urol..

[117] Gerratana L., Basile D., Buono G., De Placido S., Giuliano M., Minichillo S., Coinu A., Martorana F., De Santo I., Del Mastro L. (2018). Androgen receptor in triple negative breast cancer: A potential target for the targetless subtype. Cancer Treat. Rev..

[118] Traina T.A., Miller K., Yardley D.A., Eakle J., Schwartzberg L.S., O’Shaughnessy J., Gradishar W., Schmid P., Winer E., Kelly C. (2018). Enzalutamide for the Treatment of Androgen Receptor-Expressing Triple-Negative Breast Cancer. J. Clin. Oncol..

[119] Gucalp A., Tolaney S., Isakoff S.J., Ingle J.N., Liu M.C., Carey L.A., Blackwell K., Rugo H., Nabell L., Forero A. (2013). Phase II trial of bicalutamide in patients with androgen receptor-positive, estrogen receptor-negative metastatic Breast Cancer. Clin. Cancer Res..

[120] Traina T.A., Miller K., Yardley D.A., O’Shaughnessy J., Cortes J., Awada A., Kelly C.M., Trudeau M.E., Schmid P., Gianni L. (2015). Results from a phase 2 study of enzalutamide (ENZA), an androgen receptor (AR) inhibitor, in advanced AR+ triple-negative breast cancer (TNBC). J. Clin. Oncol..

[121] Ghosh D., Lo J., Morton D., Valette D., Xi J., Griswold J., Hubbell S., Egbuta C., Jiang W., An J. (2012). Novel aromatase inhibitors by structure-guided design. J. Med. Chem..

[122] Pereira de Jésus-Tran K., Côté P.L., Cantin L., Blanchet J., Labrie F., Breton R. (2006). Comparison of crystal structures of human androgen receptor ligand-binding domain complexed with various agonists reveals molecular determinants responsible for binding affinity. Protein Sci..

[123] Spinello A., Pavlin M., Casalino L., Magistrato A. (2018). A Dehydrogenase Dual Hydrogen Abstraction Mechanism Promotes Estrogen Biosynthesis: Can We Expand the Functional Annotation of the Aromatase Enzyme?. Chemistry.

[124] Gaulton A., Bellis L.J., Bento A.P., Chambers J., Davies M., Hersey A., Light Y., McGlinchey S., Michalovich D., Al-Lazikani B. (2012). ChEMBL: A large-scale bioactivity database for drug discovery. Nucleic Acids Res..

[125] Morris G.M., Huey R., Lindstrom W., Sanner M.F., Belew R.K., Goodsell D.S., Olson A.J. (2009). AutoDock4 and AutoDockTools4: Automated docking with selective receptor flexibility. J. Comput. Chem..

[126] Dallakyan S., Olson A.J. (2015). Small-molecule library screening by docking with PyRx. Methods Mol. Biol..

[127] Thompson E.A., Siiteri P.K. (1974). The involvement of human placental microsomal cytochrome P-450 in aromatization. J. Biol. Chem..

[128] Heldring N., Pawson T., McDonnell D., Treuter E., Gustafsson J.A., Pike A.C. (2007). Structural insights into corepressor recognition by antagonist-bound estrogen receptors. J. Biol. Chem..

[129] EDSP-USEP Agency (2011). Aromatase Assay (Human Recombinant) OCSPP Guideline 890.1200.

[130] Reif D.M., Sypa M., Lock E.F., Wright F.A., Wilson A., Cathey T., Judson R.R., Rusyn I. (2013). ToxPi GUI: An interactive visualization tool for transparent integration of data from diverse sources of evidence. Bioinformatics.

