# Supplementary material for: Molecular Targets of Minor Cannabinoids in Breast Cancer: In Silico and In Vitro Studies
Source: Pharmaceuticals (Basel). 2024 Sep 21;17(9):1245. doi: 10.3390/ph17091245 (PMC11434916; doi:10.3390/ph17091245)

Supplementary Figure S1

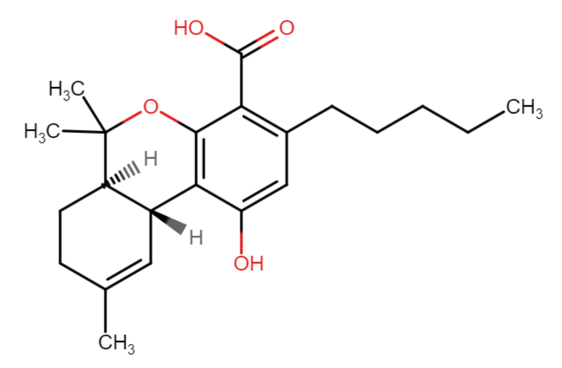

(-)-Δ^9-trans-tetrahydrocannabinolic acid B

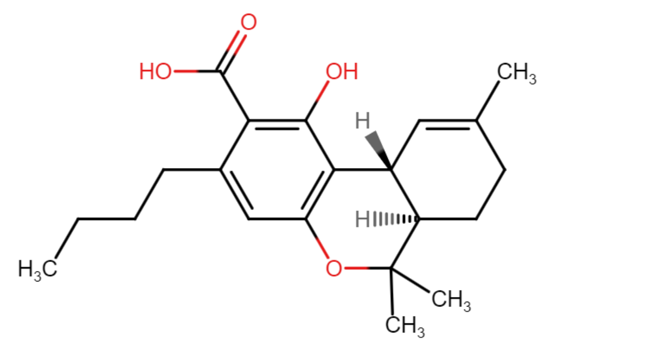

(-)-Δ^9-trans-tetrahydrocannabinolic acid A-C_4

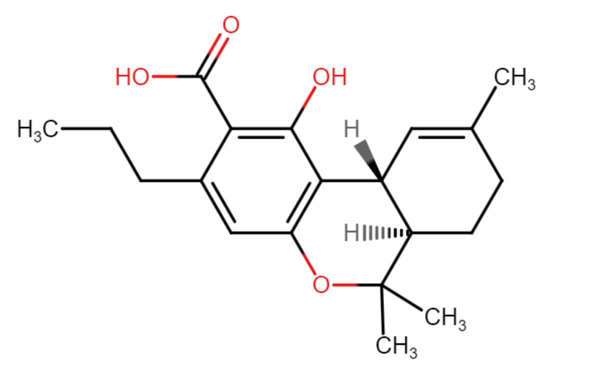

(-)-Δ^9-trans-tetrahydrocannabivarinic acid

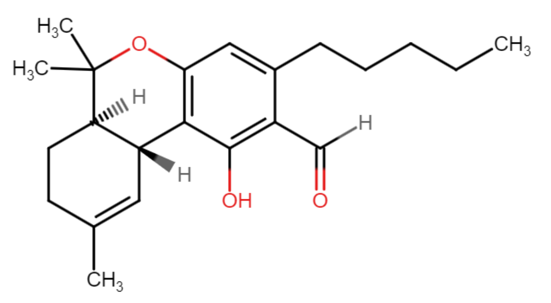

(-)-Δ^9-trans-tetrahydrocannabinal

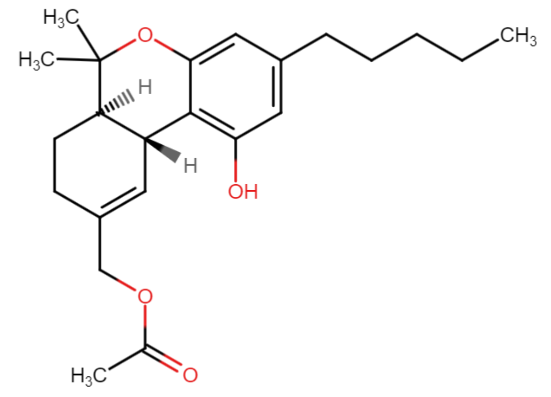

11-acetoxy-(-)- Δ^9-trans-tetrahydrocannabinolic acid A

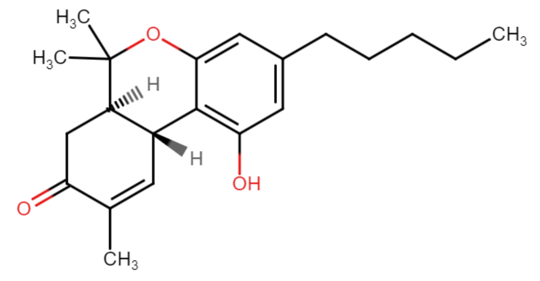

8-oxo-(-)- Δ^9-trans-tetrahydrocannabinol

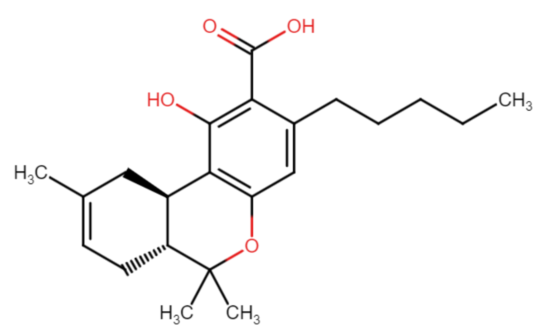

Δ^8-trans-tetrahydrocannabinolic acid A

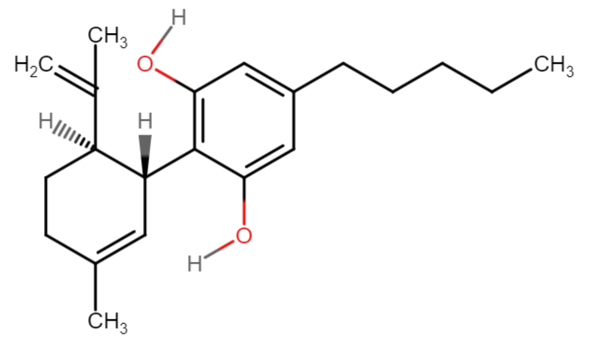

Cannabidiol

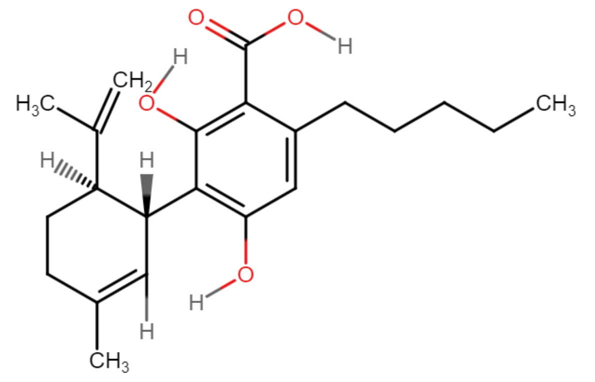

Cannabidiolic acid

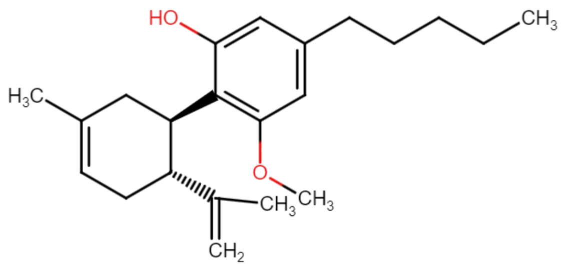

Cannabidiol monomethylether

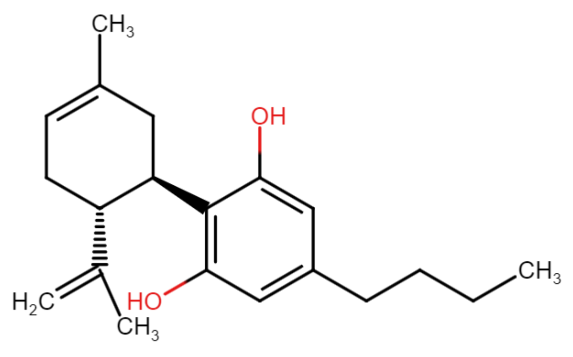

Cannabidiol-C_4

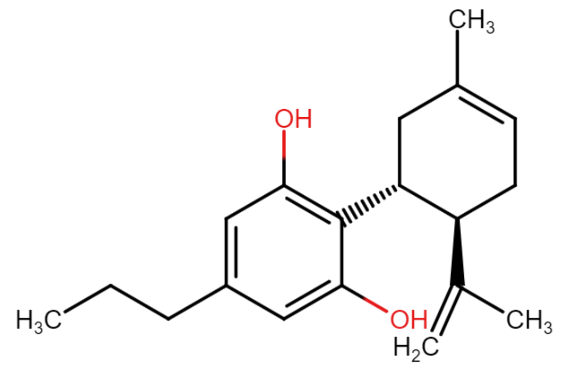

(-)-cannabidivarin

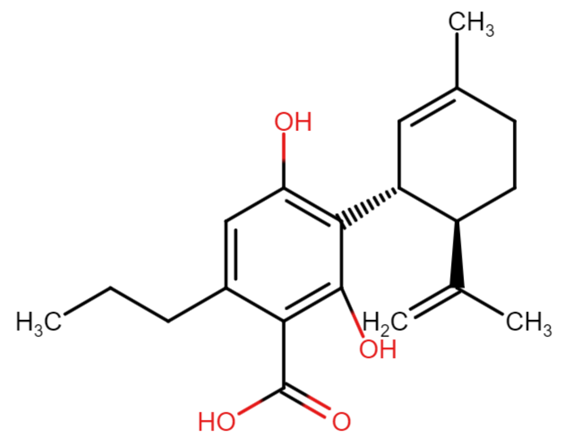

Cannabidivarinic acid

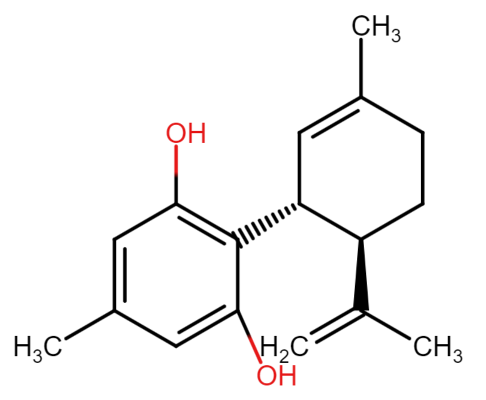

Cannabidiorcol

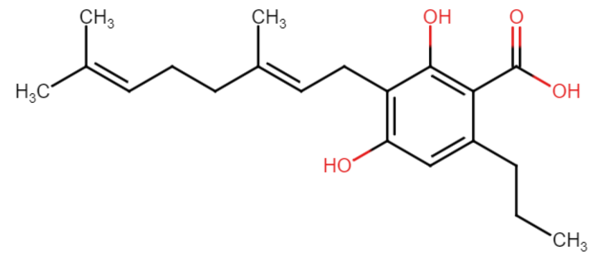

Cannabigerovarinic acid

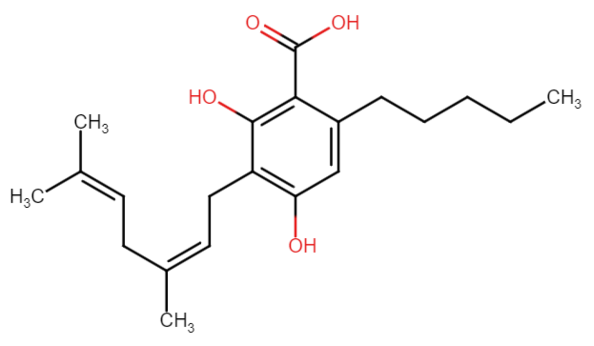

Cannabinerolic acid

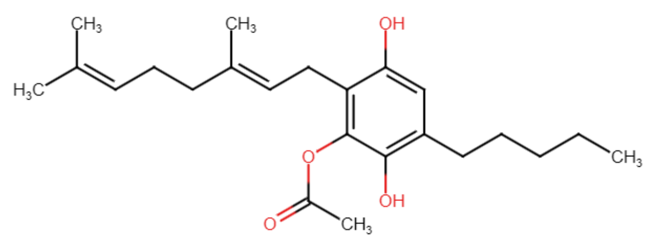

5-acetyl-4-hydroxy-cannabigerol

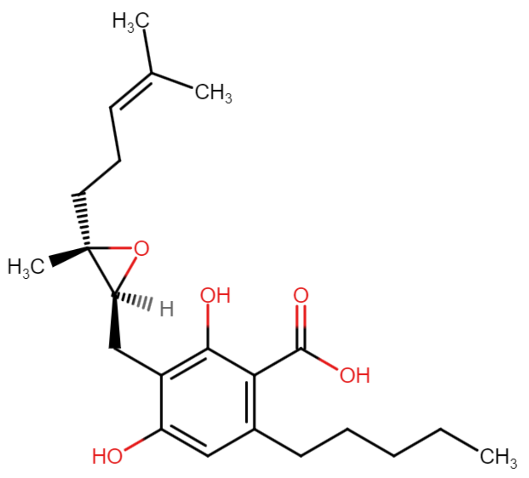

(±)-6,7-trans-epoxycannabigerolic acid

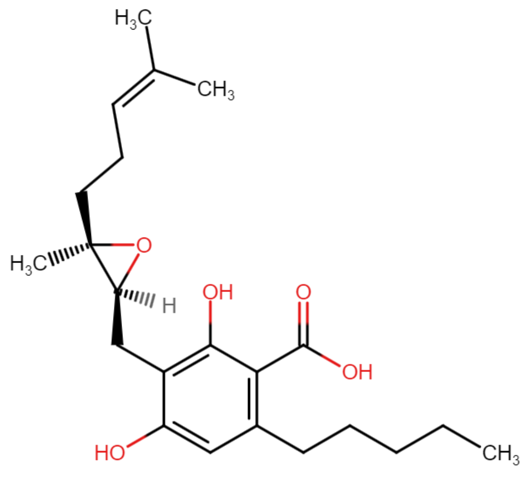

(±)-6,7-cis-epoxycannabigerolic acid

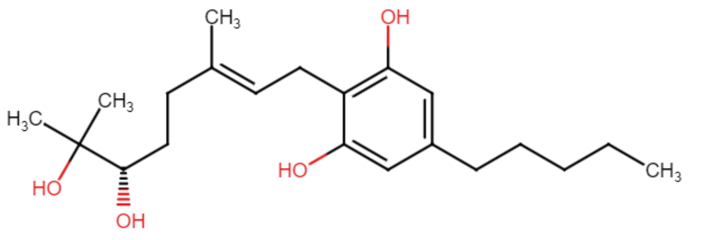

Camagerol

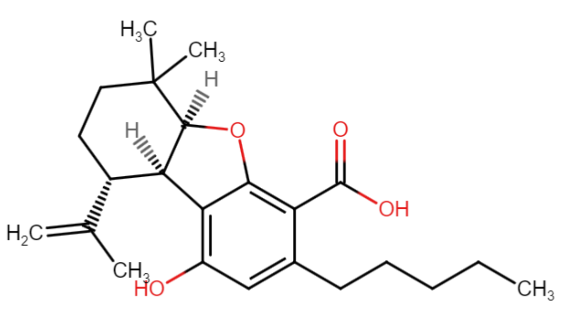

Cannabielsoin acid A

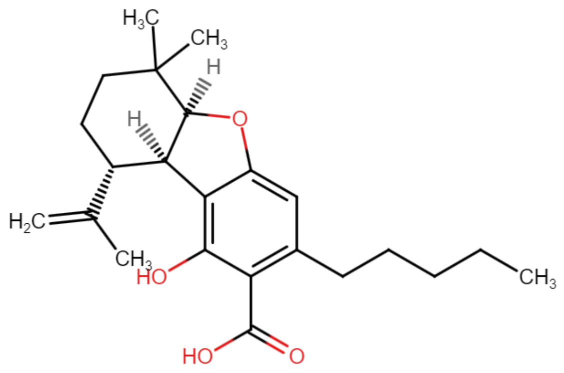

Cannabielsoin acid B

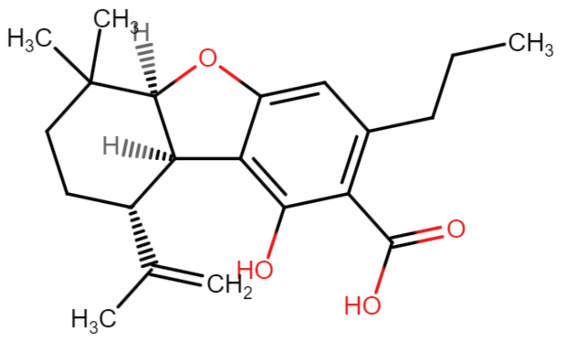

Cannabielsoic acid B-C_3

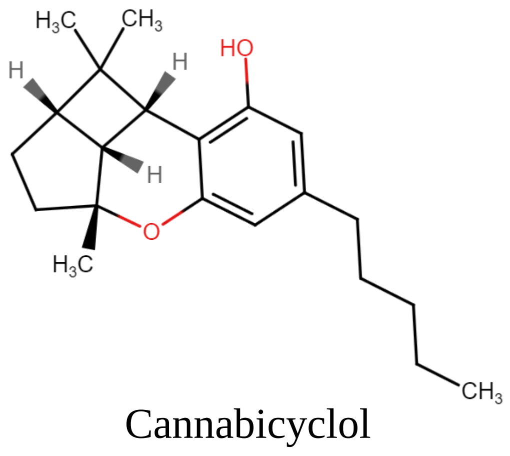

Cannabicyclol

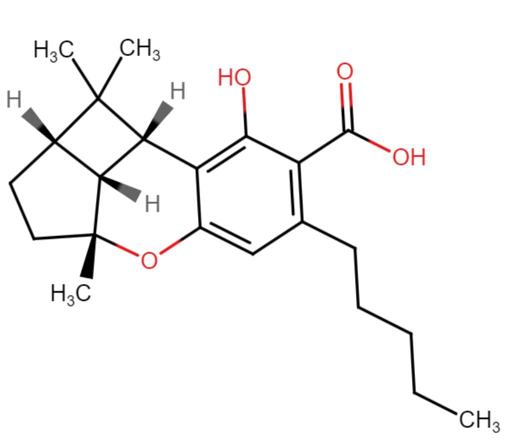

Cannabicyclolic acid

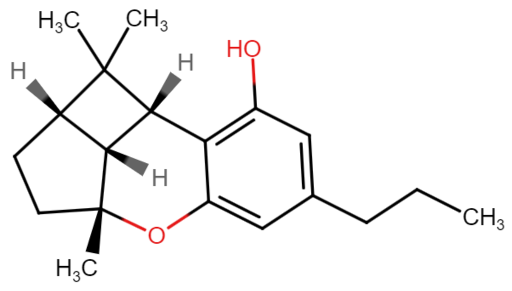

Cannabicyclovarin

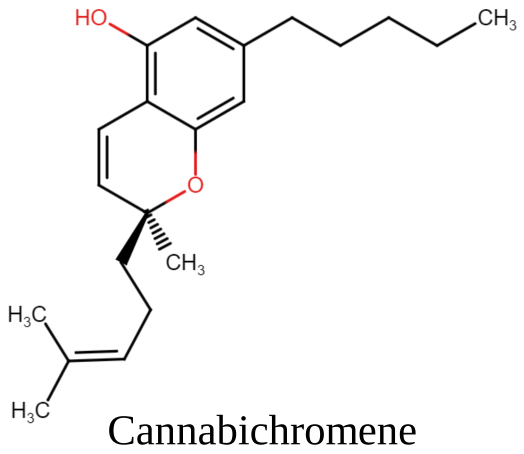

Cannabichromene

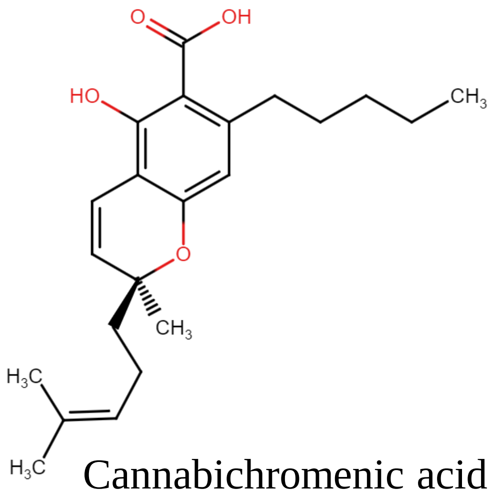

Cannabichromenic acid

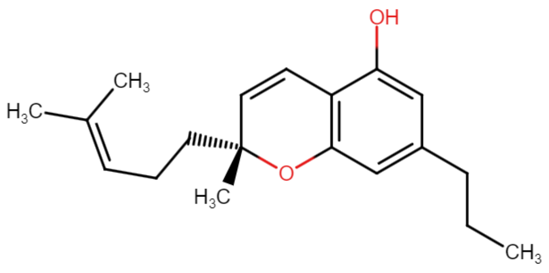

Cannabichromevarin

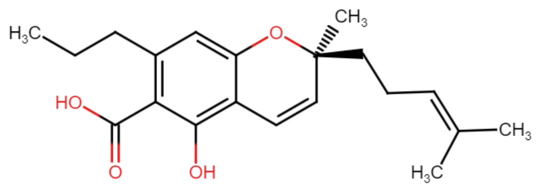

Cannabichromevarinic acid

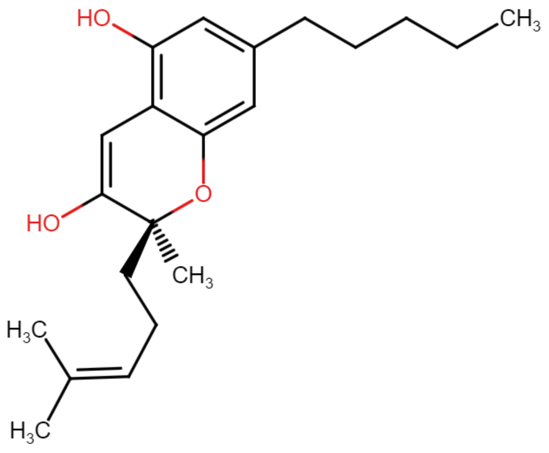

(-)-7-hydroxycannabichromene

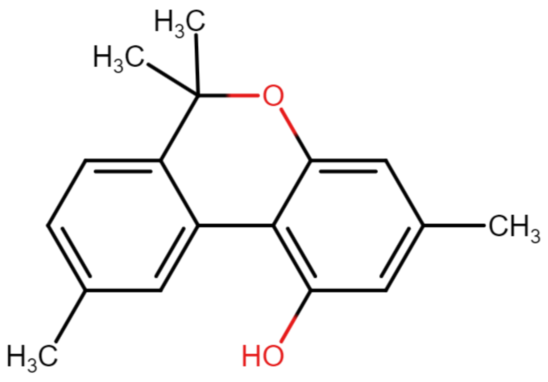

Cannabiorcol-C_1

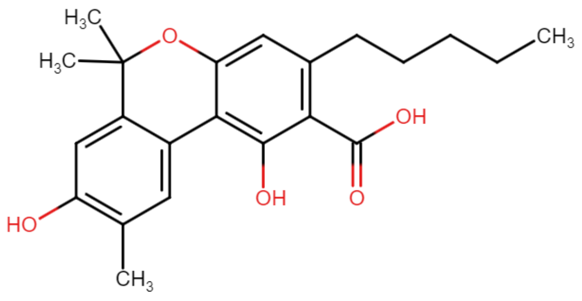

8-hydroxycannabinolic acid A

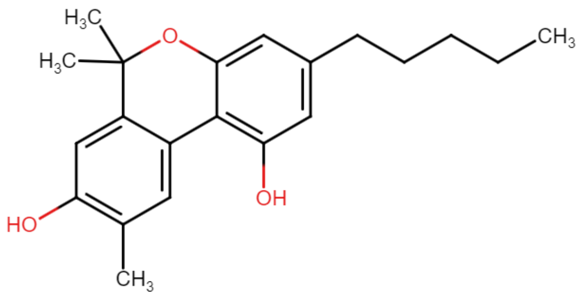

8-hydroxycannabinol

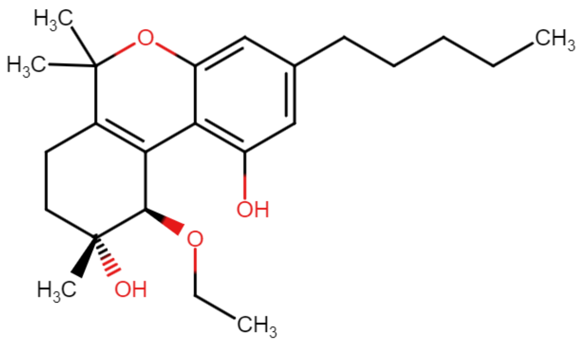

(-)-trans-cannabitriol-OEt-C_5

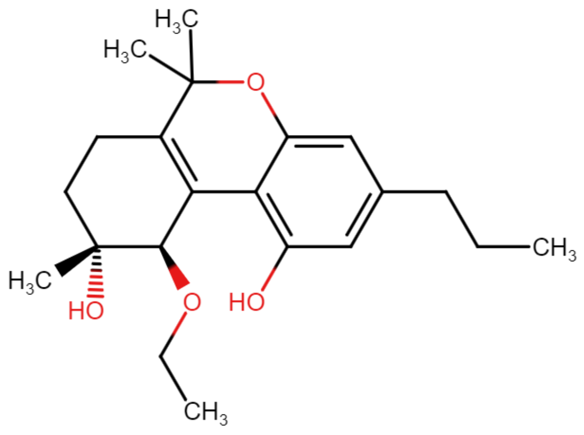

(-)-trans-cannabitriol-OEt-C_3

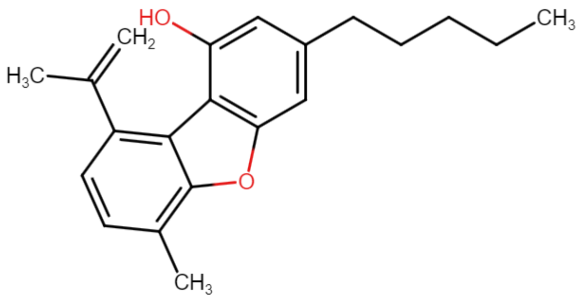

Dehydrocannabifuran

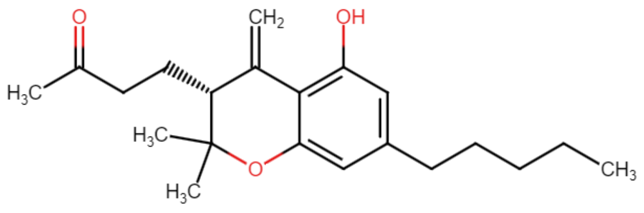

Cannabicoumaronome-C_5

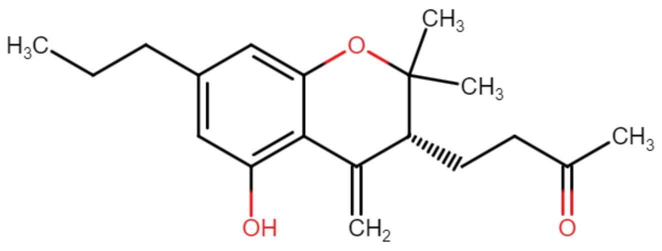

Cannabichromanone-C_3

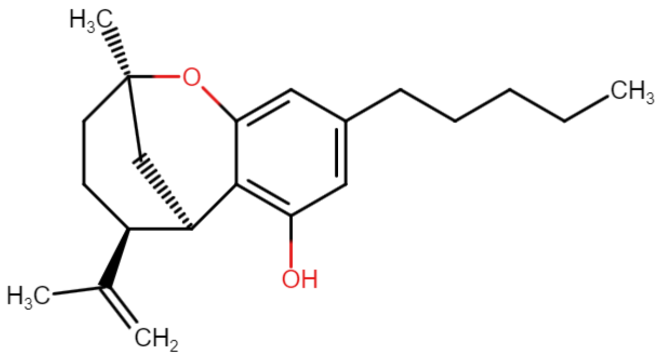

(-)-Δ7-trans-(1R, 3R, 6R)-isotetrahydrocannabinol-C_5

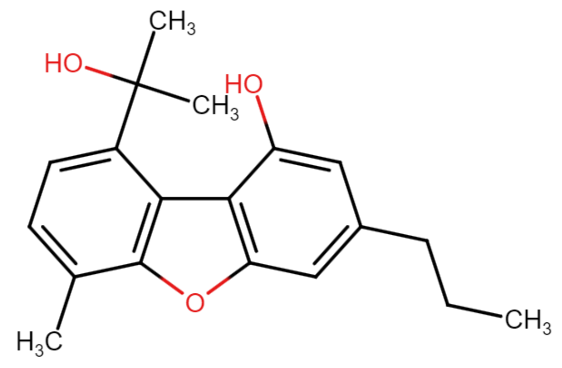

8-hydroxy-isohexahydrocannabivarin

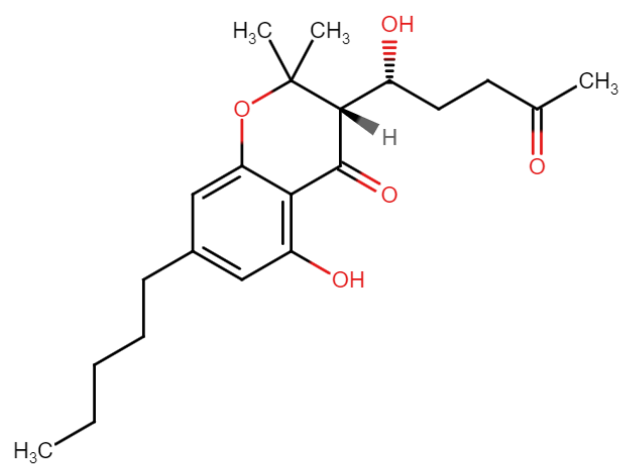

Cannabichromanone B

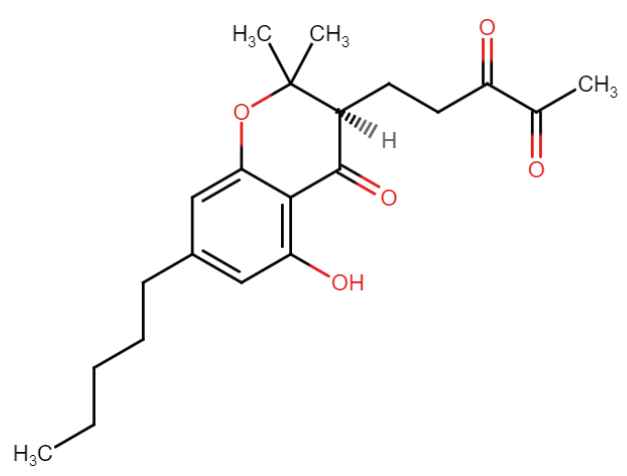

Cannabichromanone C

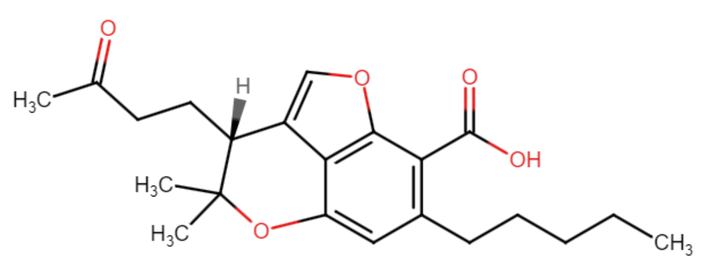

(-)-(7R)-cannabicoumarononic acid

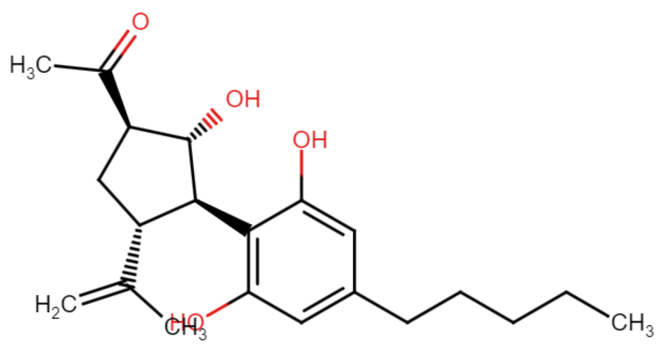

Cannabimovone

Supplementary Figure S2

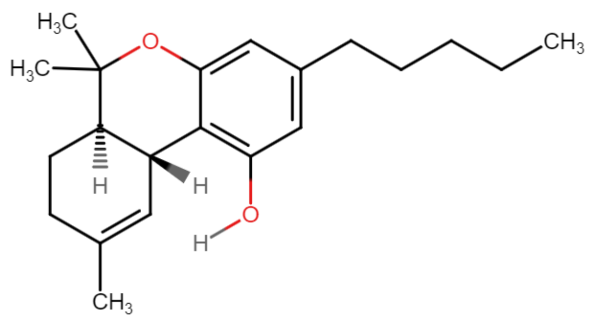

Δ^{9}-tetrahydrocannabinol

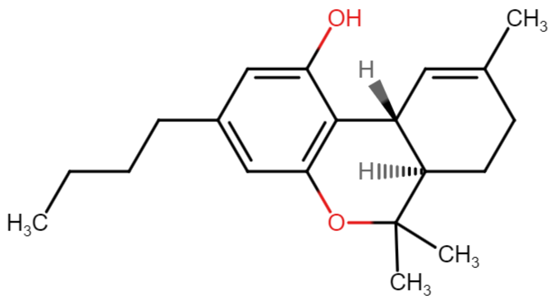

(-)-Δ^{9}-trans-tetrahydrocannabinol-C_4

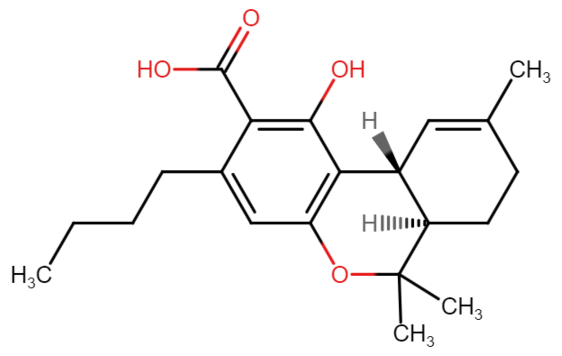

(-)-Δ^{9}-trans- tetrahydrocannabinolic acid A-C_4

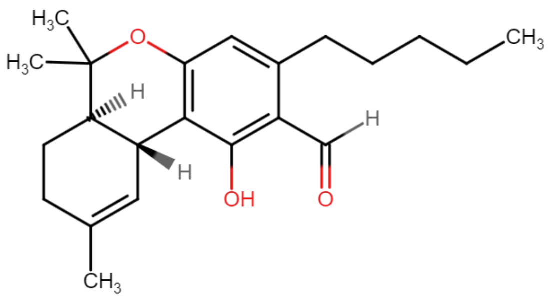

(-)-Δ^{9}-trans- tetrahydrocannabinal

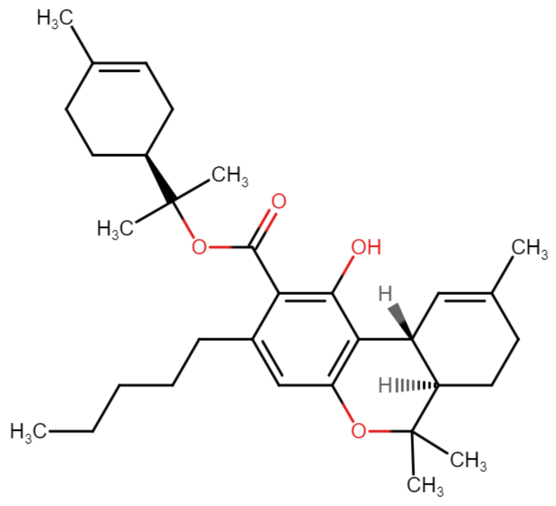

α-terpenyl (-)-Δ^{9}-trans-tetrahydrocannabinolate

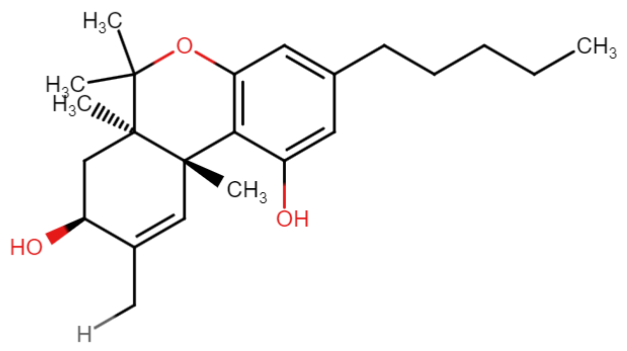

4-terpenyl (-)-Δ^{9}-trans-tetrahydrocannabinolate

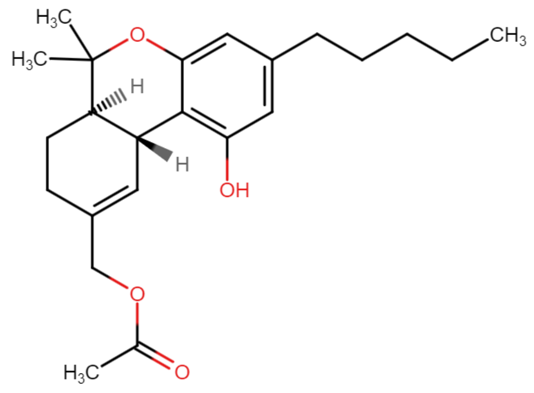

11-acetoxy-(-)-Δ^{9}-trans-tetrahydrocannabinolic acid A

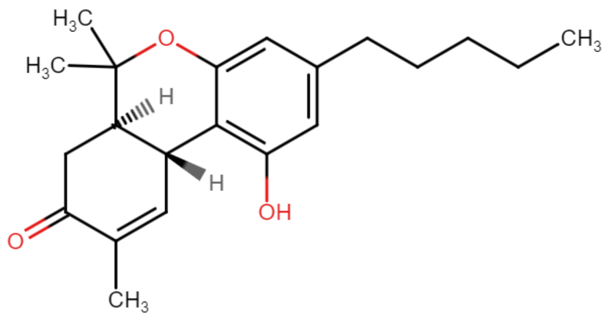

8-oxo-(-)-Δ^{9}-trans-tetrahydrocannabinol

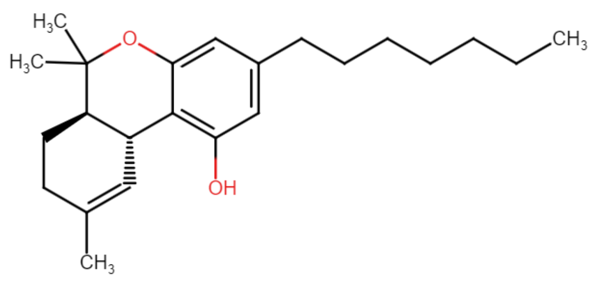

(-)-Δ^{9}-trans-tetrahydrocannabiphorol

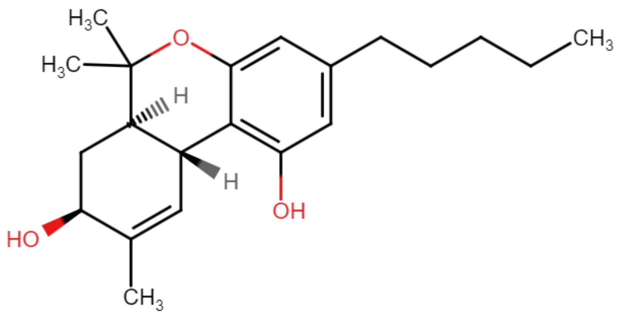

(-)-Δ^{9}-trans-tetrahydrocannabihexol

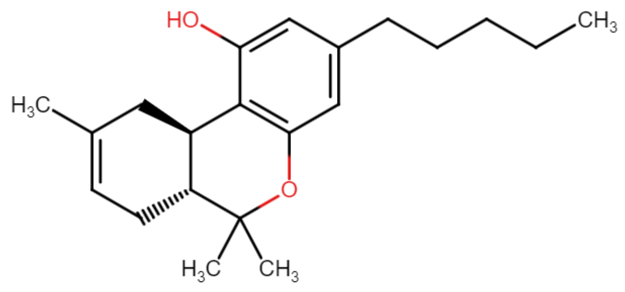

(-)-Δ^{8}-trans-tetrahydrocannabinol

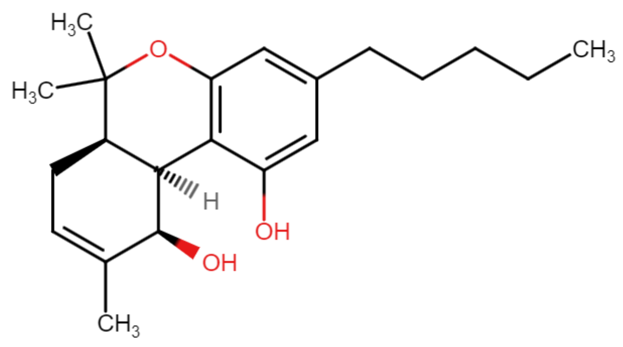

10α-hydroxy-Δ^{8}-tetrahydrocannabinol

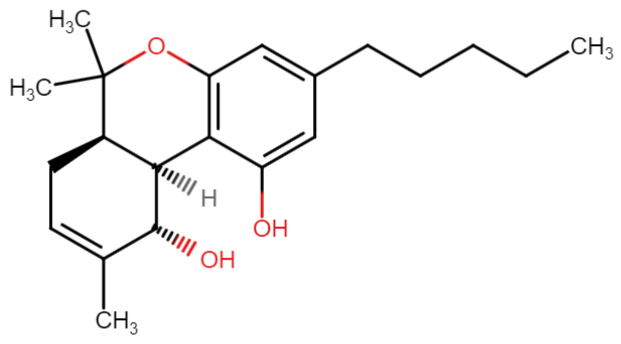

10β-hydroxy-Δ^{8}-tetrahydrocannabinol

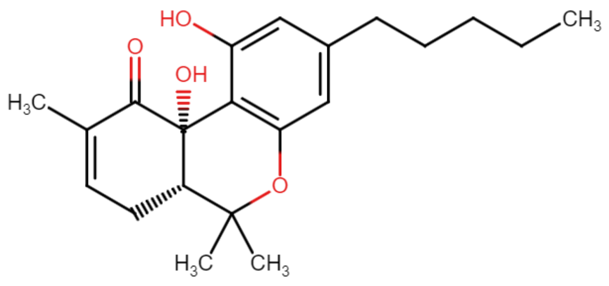

10a-α-hydroxy-o-oxo- Δ^{8}-tetrahydrocannabinol

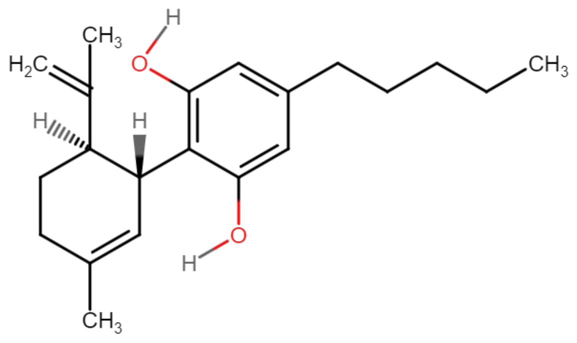

Cannabidiol

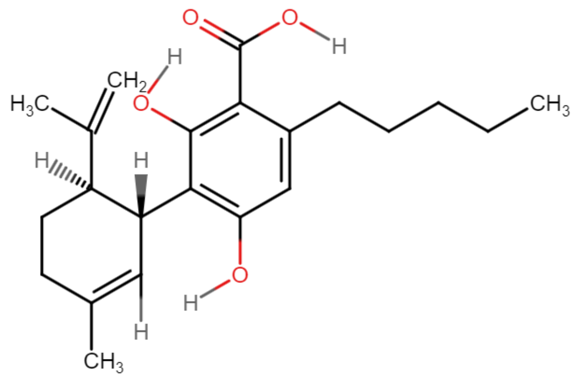

Cannabidiolic acid

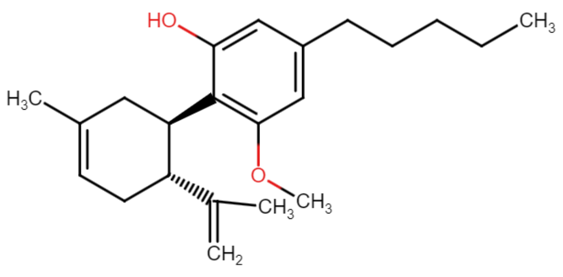

Cannabidiol monomethylether

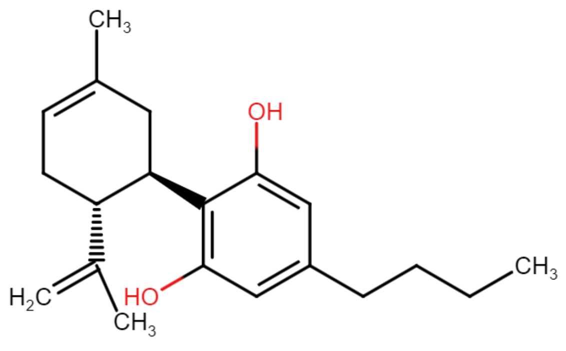

Cannabidiol-C_4

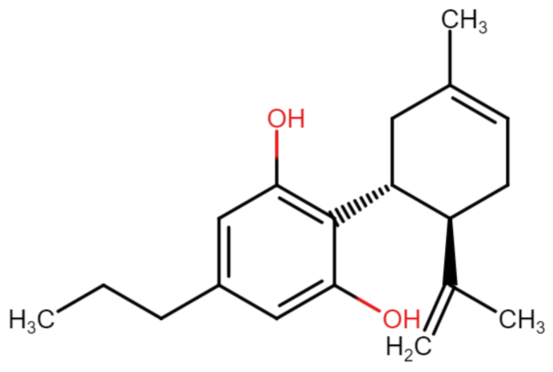

(-)-cannabidivarin

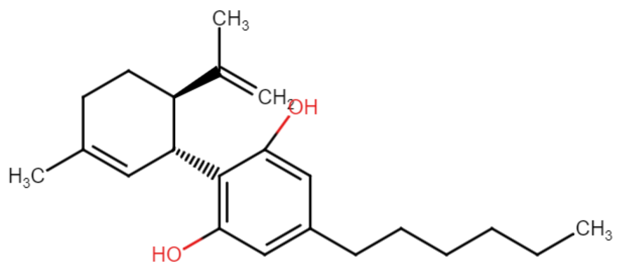

Cannabidihexol

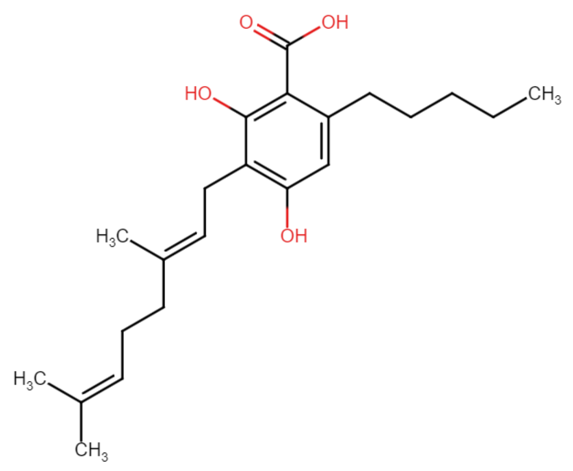

Cannabigerolic acid

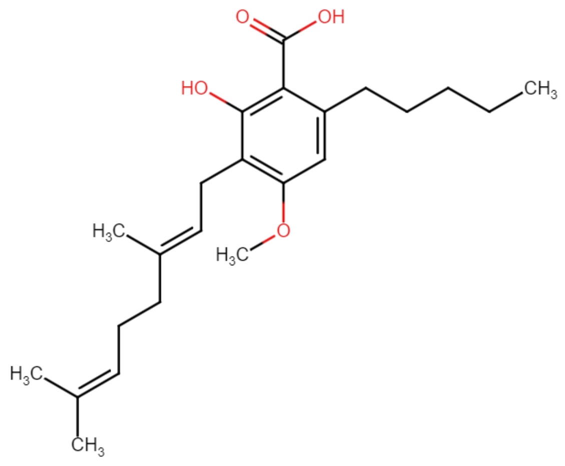

Monomethyl ether of cannabigerolic acid

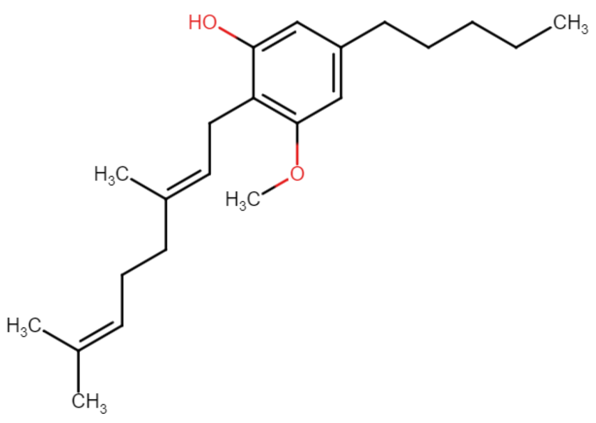

Monomethyl ether of (E)-cannabigerol

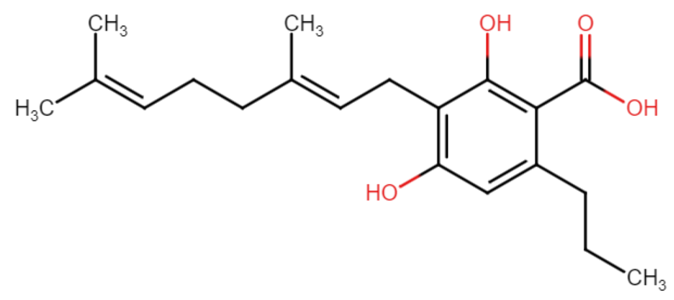

Cannabigerovarinic acid

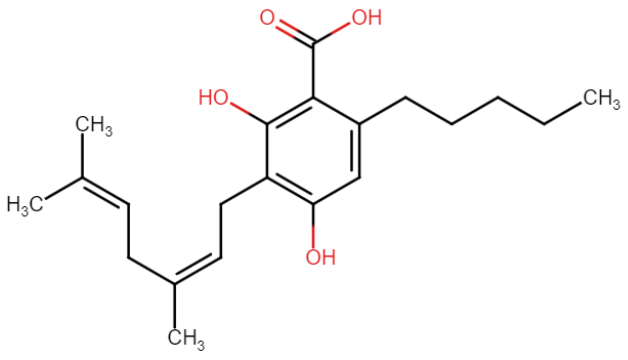

Cannabinerolic acid

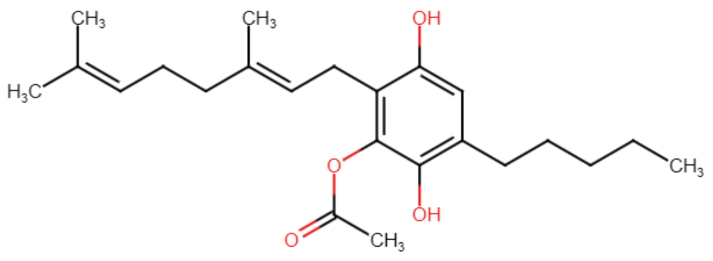

5-acetyl-4-hydroxy-cannabigerol

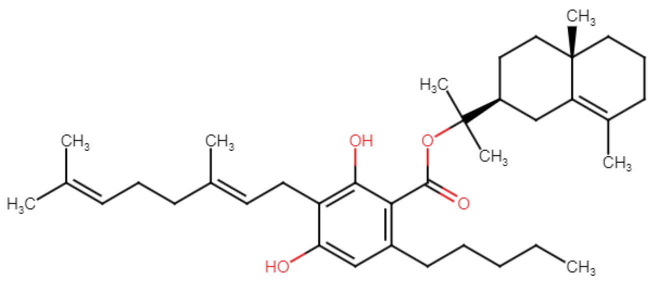

γ-eudesmyl-cannabigerolate

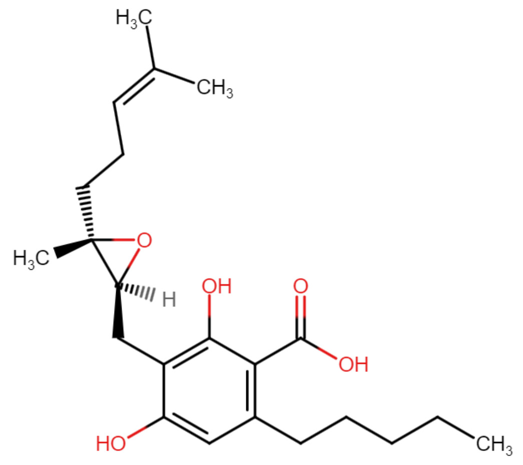

(±)-6,7-trans-epoxycannabigerolic acid

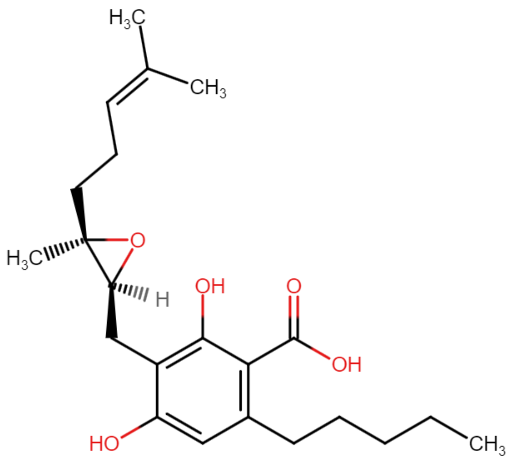

(±)-6,7-cis-epoxycannabigerolic acid

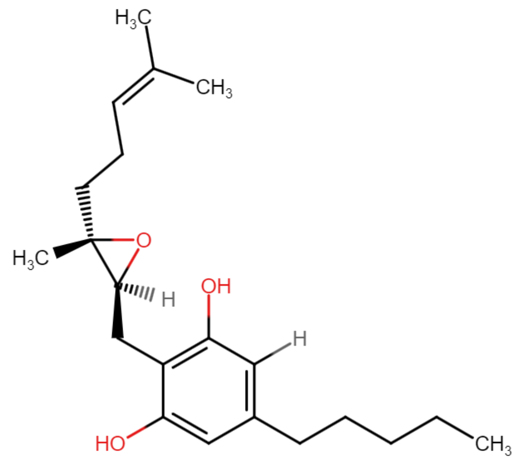

(±)-6,7-cis-epoxycannabigerol

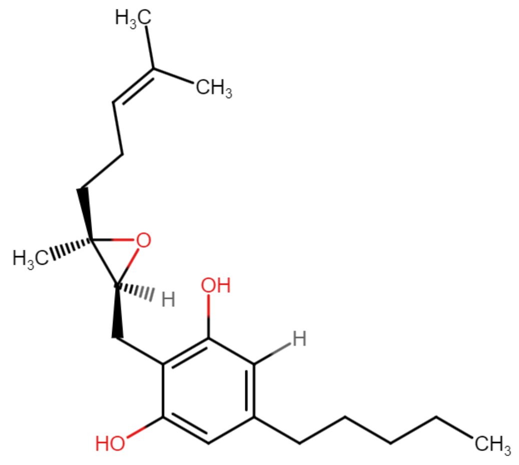

(±)-6,7-trans-epoxycannabigerol

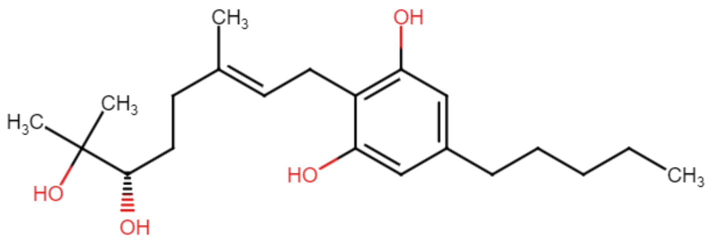

Camagerol

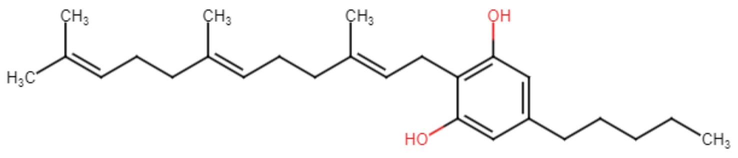

Sesquicannabigerol

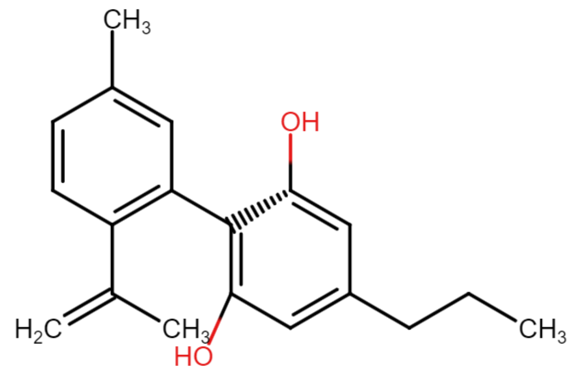

Cannabinodivirin

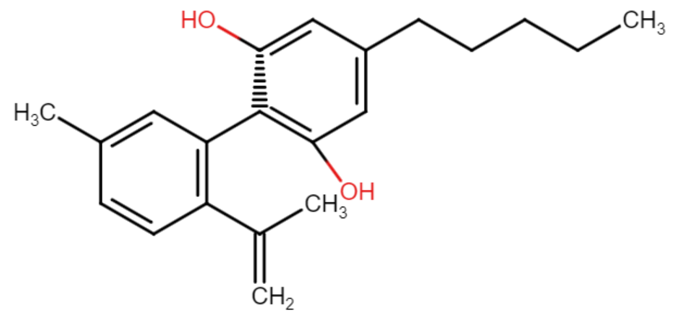

Cannabinodiol

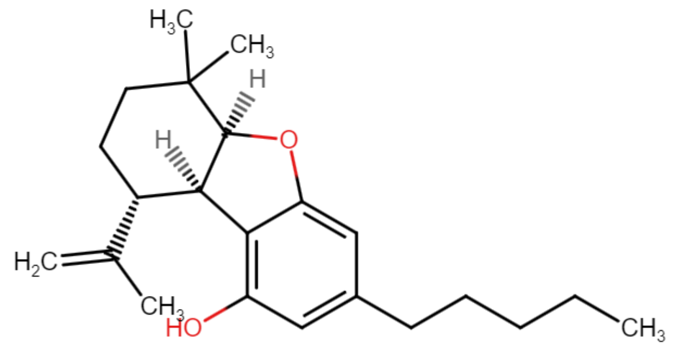

Cannabielsoin

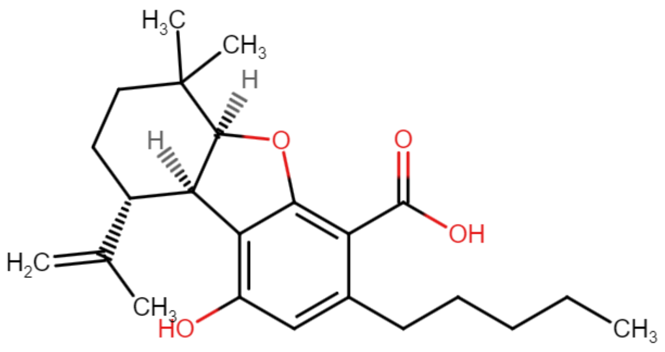

Cannabielsoin acid A

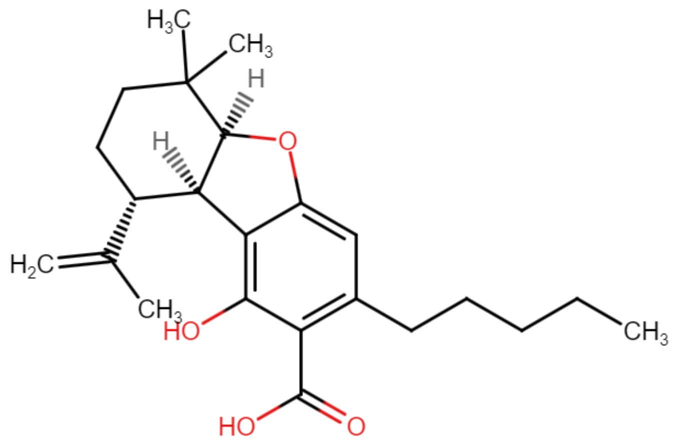

Cannabielsoin acid B

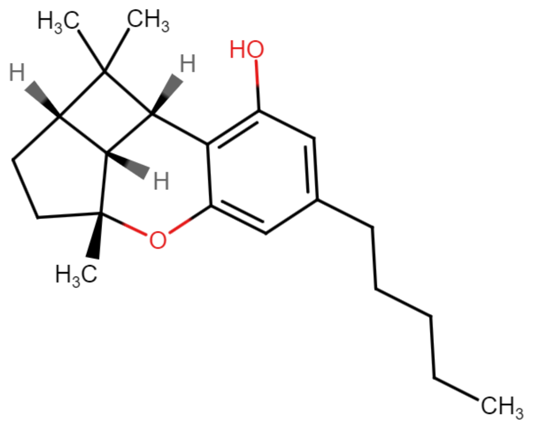

Cannabicyclol

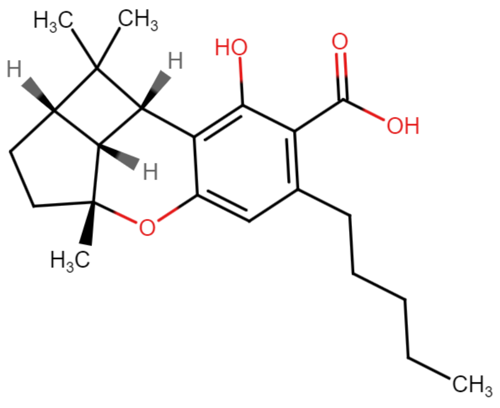

Cannabicyclolic acid

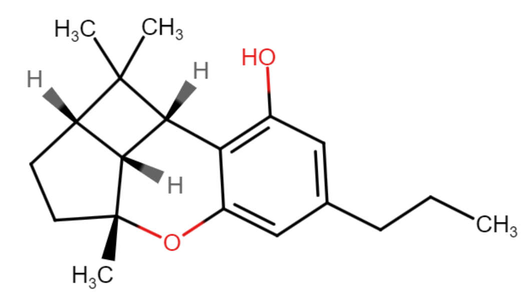

Cannabicyclovarin

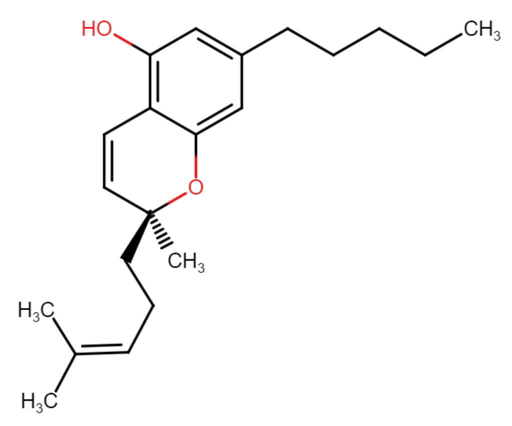

Cannabichromene

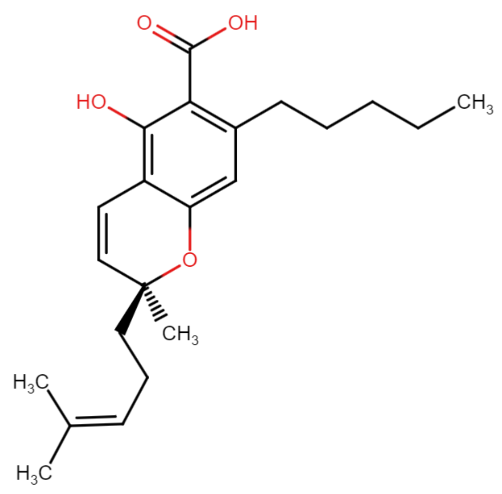

Cannabichromenic acid

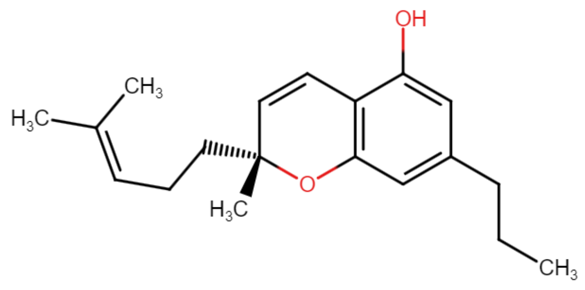

Cannabichromevarin

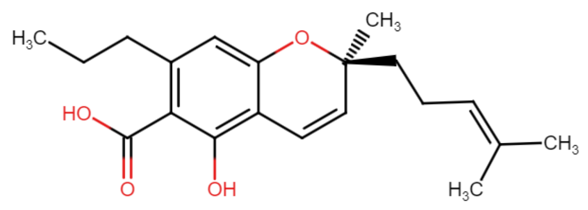

Cannabichromevarinic acid

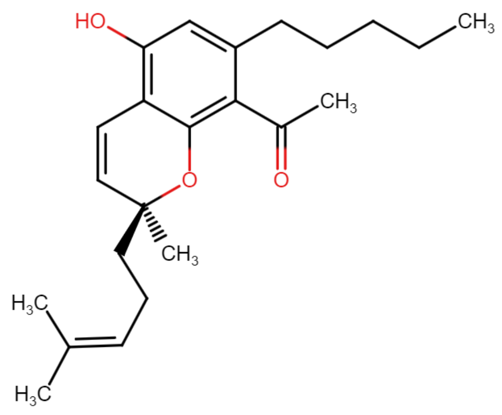

(±)-4-acetoxycannabichromene

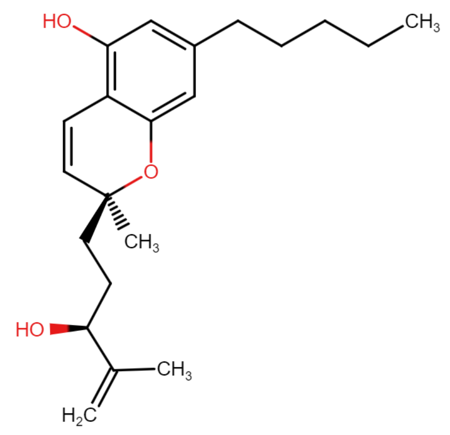

(±)-3''-hydroxy- $\Delta^{4''}$-cannabichromene

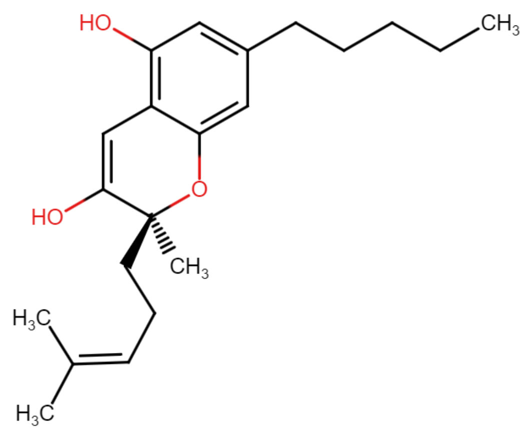

(-)-7-hydroxycannabichromene

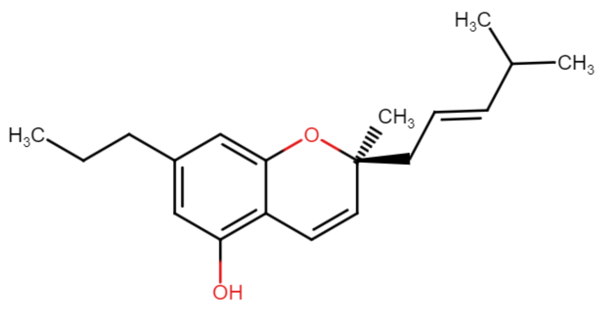

2-methyl-2-(4-methyl-2-pentyl)-7-propyl-2H-1-benzopyran-5-ol

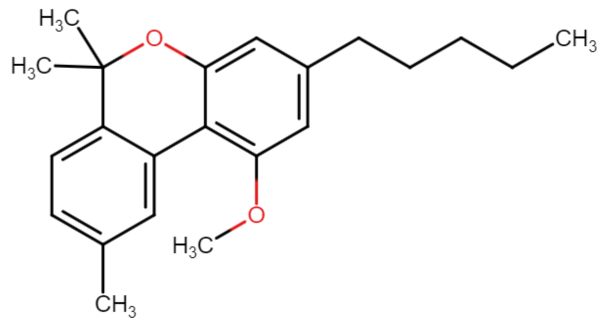

Cannabinol methyl ether

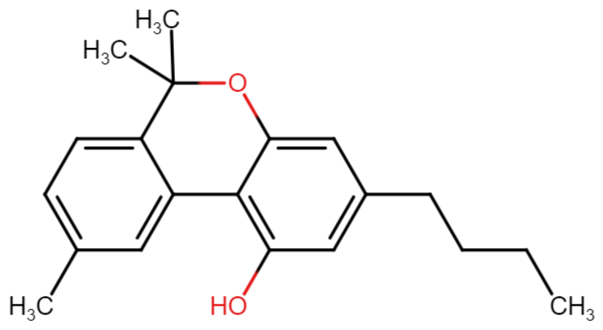

Cannabinol-C_4

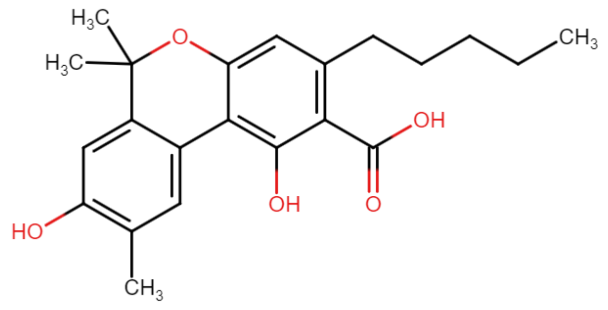

8-hydroxycannabinolic acid A

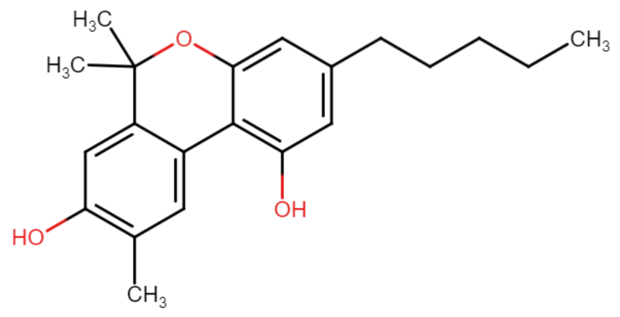

8-hydroxycannabinol

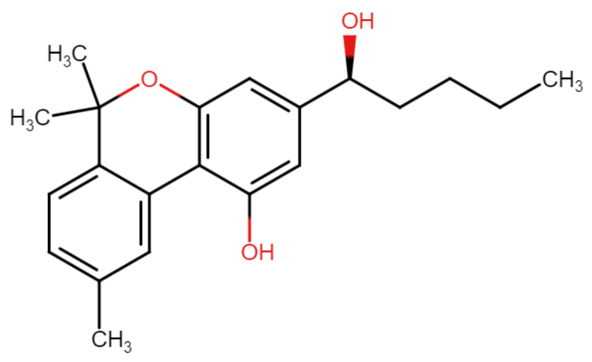

(10S)-hydroxycannabinol

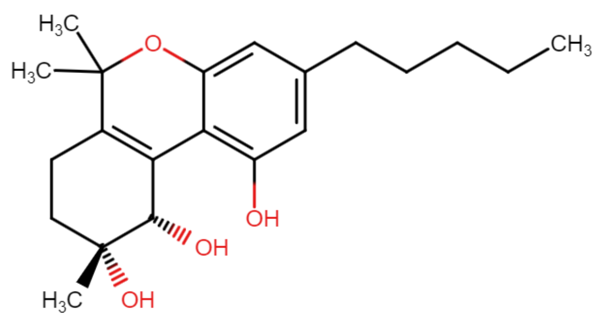

Cannabitriol

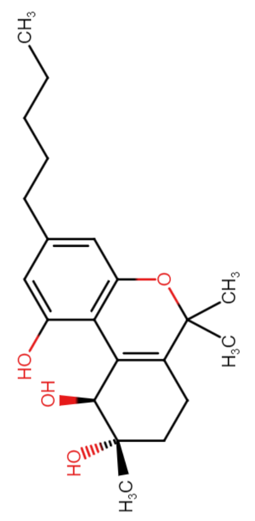

(+)-trans-cannabitriol-C_5

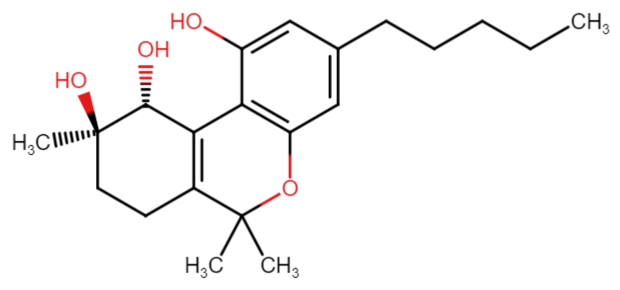

(-)-trans-cannabitriol-C_5

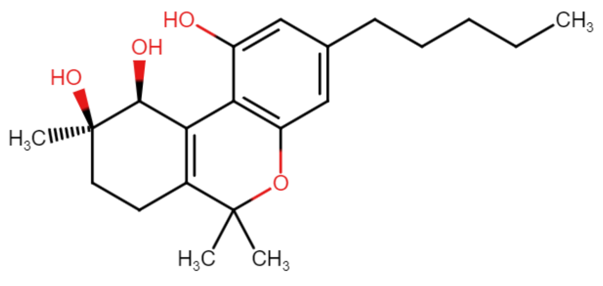

(+)-cis-cannabitriol-C_5

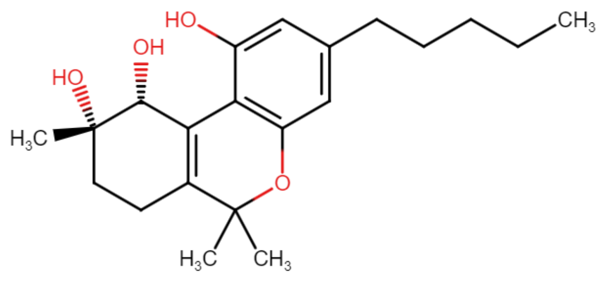

(-)-cis-cannabitriol-C_5

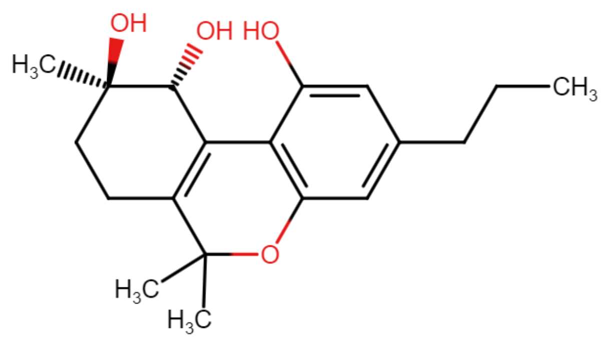

(+)-trans-cannabitriol-C_5

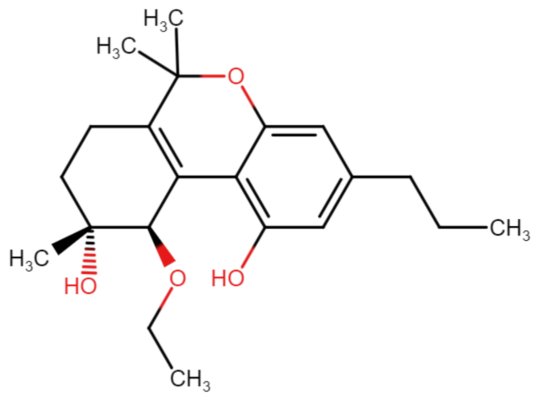

(-)-trans-cannabitriol-OEt-C_3

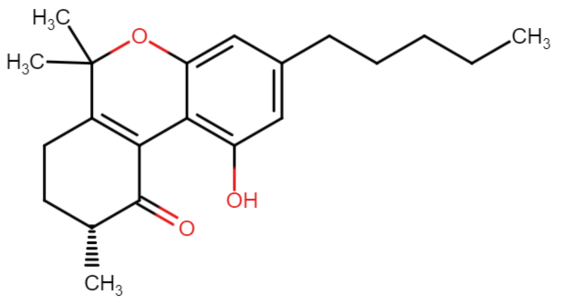

10-oxo- $\Delta^{6a(10a)}$-tetrahydrocannabinol

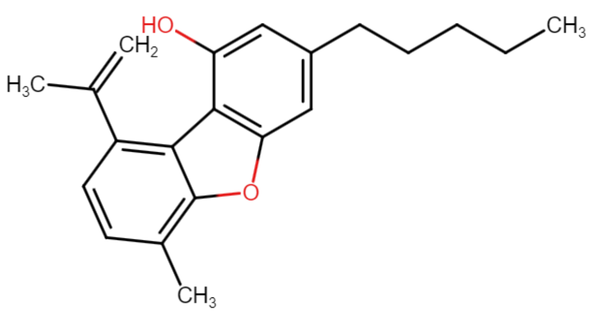

Dehydrocannabifuran

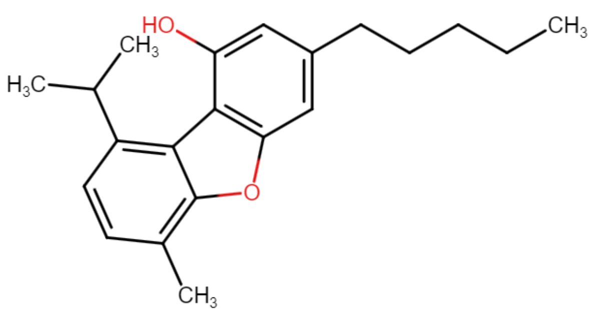

Cannabifuran

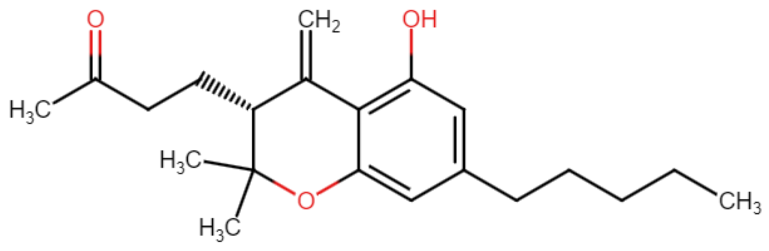

Cannabicoumaronome-C_5

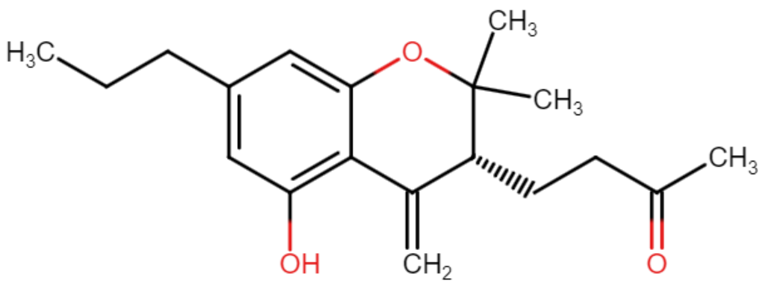

Cannabichromanone-C_3

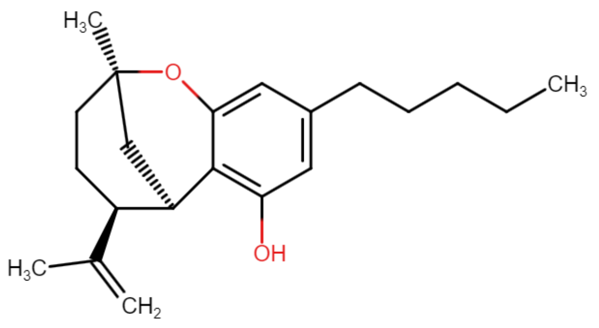

(-)-Δ^7-trans-(1R, 3R, 6R)-isotetrahydrocannabinol-C_5

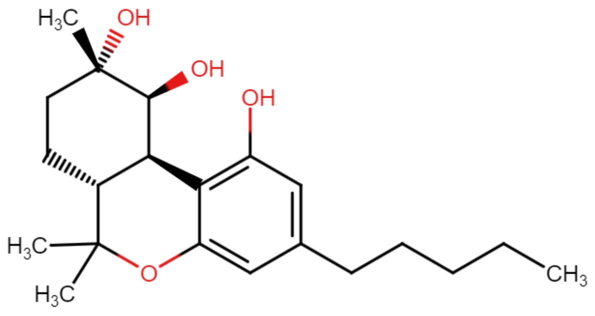

(-)-cannabiripsol-C_5

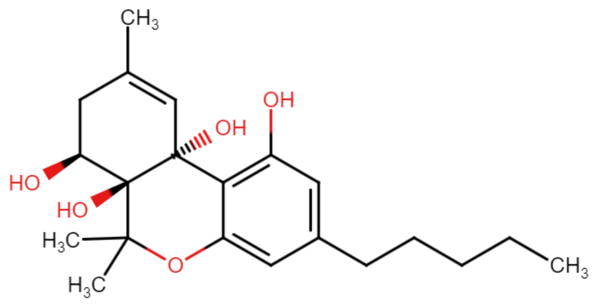

(-)-Cannabitetrol

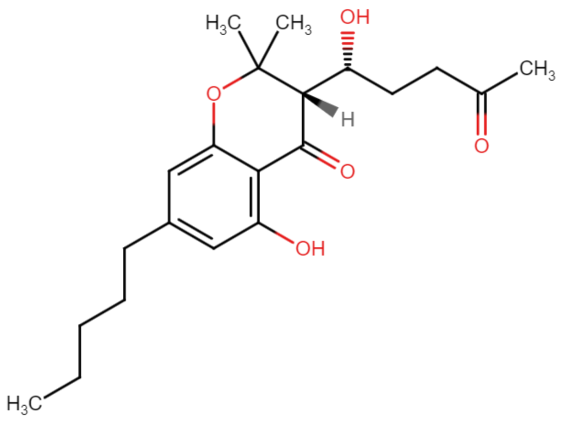

Cannabichromanone B

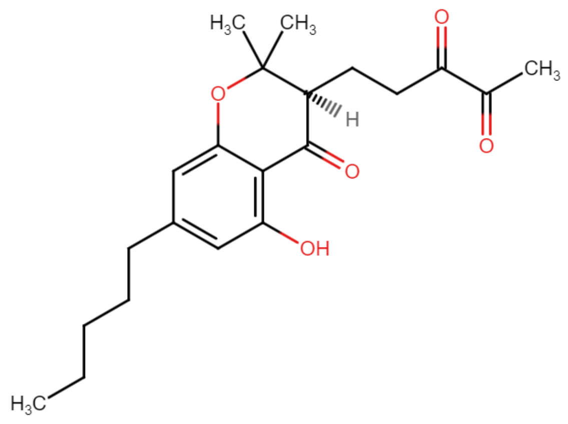

Cannabichromanone C

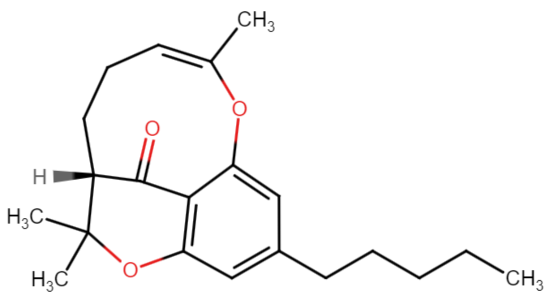

Cannabichromanone D

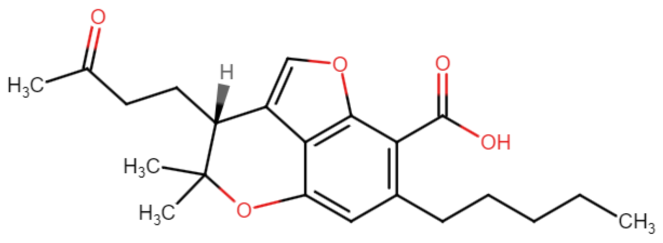

(-)-(7R)-cannabicoumaronic acid

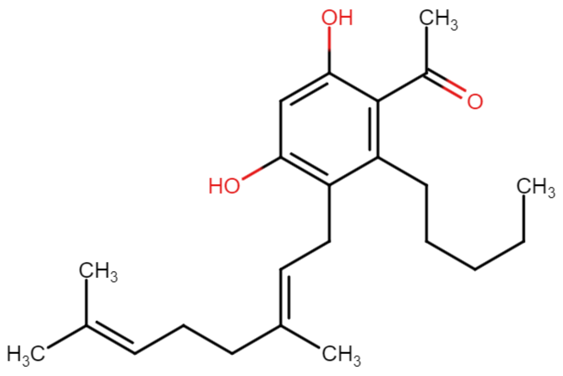

4-acetoxy-2-geranyl-5-hydroxy-3-n-pentylphenol

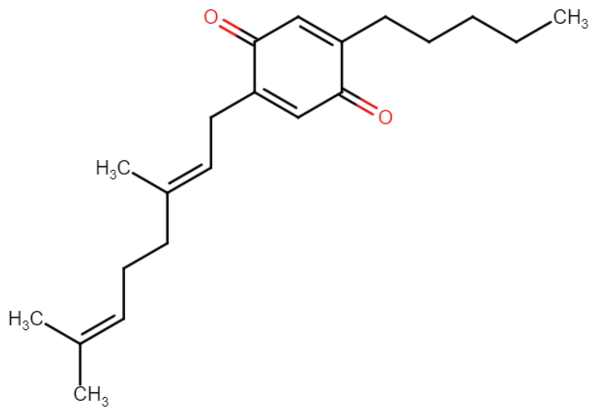

2-geranyl-5-hydroxy-3-n-pentyl-1,4-benzoquinone

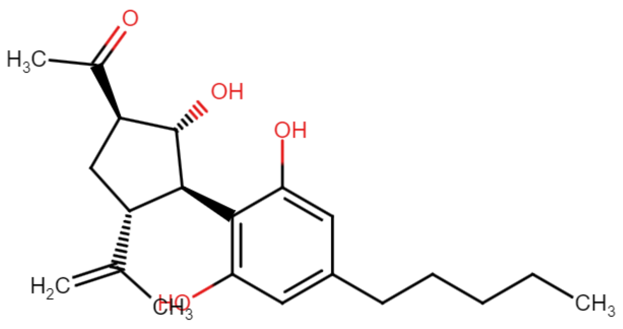

Cannabimovone

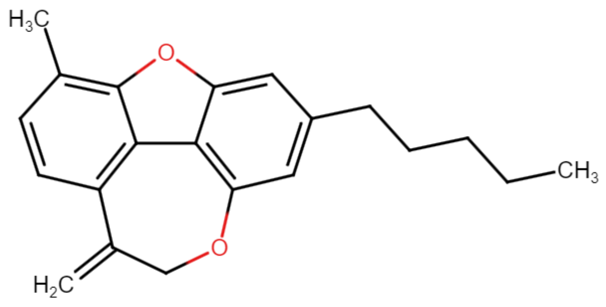

Cannabioxepane

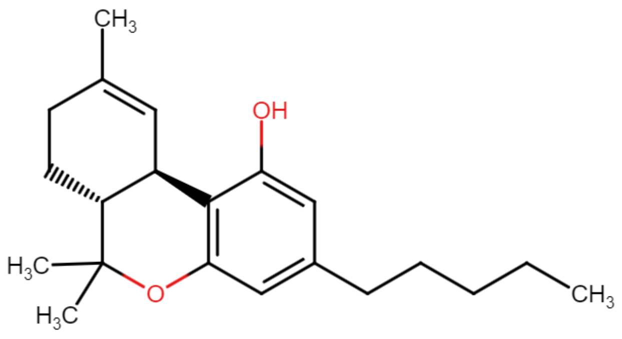

(-)-Δ^9-cis-(6aS, 10aR)-tetrahydrocannabinol

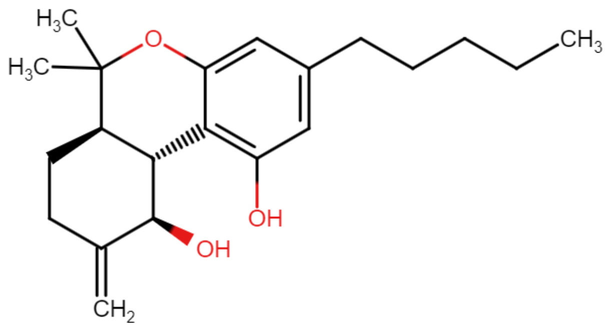

10α-hydroxy-$\Delta^{9,11}$-hexahydrocannabinol

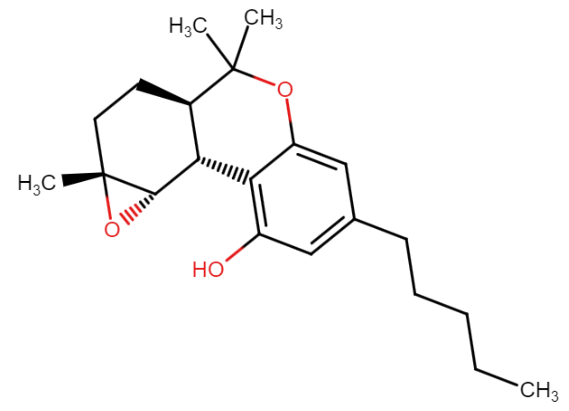

9β, 10β-epoxyhexahydrocannabinol

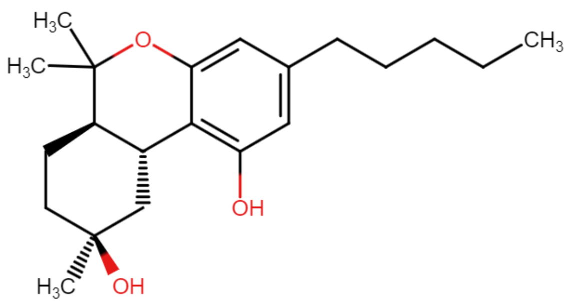

9α-hydroxyhexahydrocannabinol

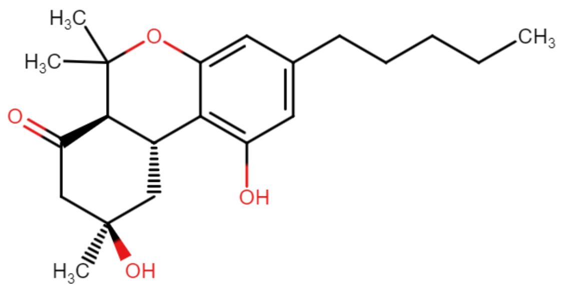

7-oxo-9α-hydroxyhexahydrocannabinol

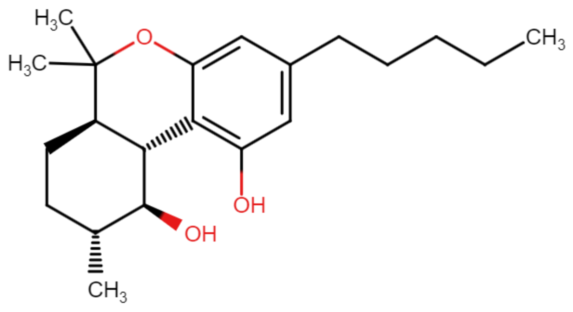

10α-hydroxyhexahydrocannabinol

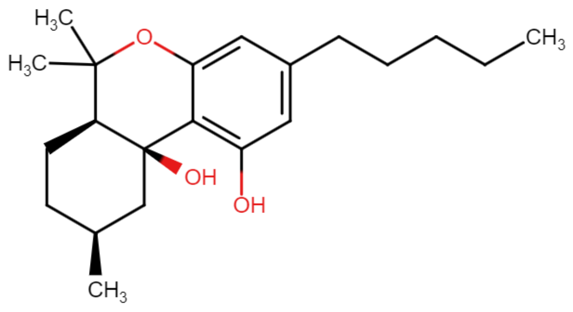

10αR-hydroxyhexahydrocannabinol

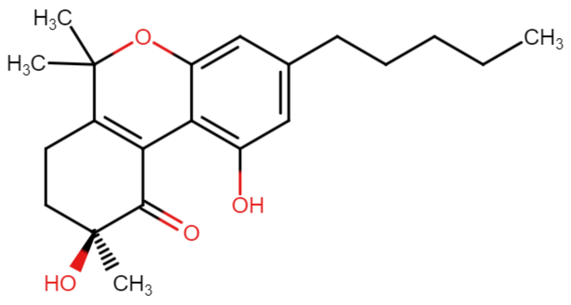

9α-hydroxy-10-oxo- $\Delta^{6a,10a}$-tetrahydrocannabinol

Supplementary Figure S3

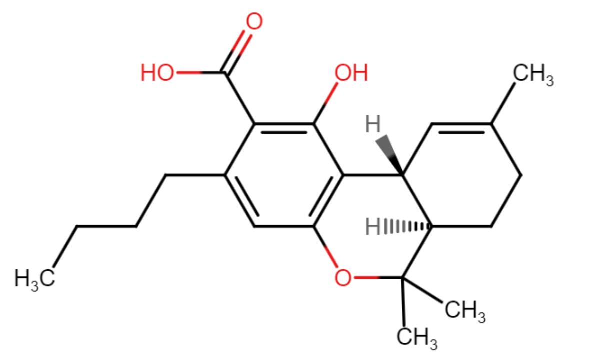

(-)-Δ^{9}-trans- tetrahydrocannabinolic acid A-C_4

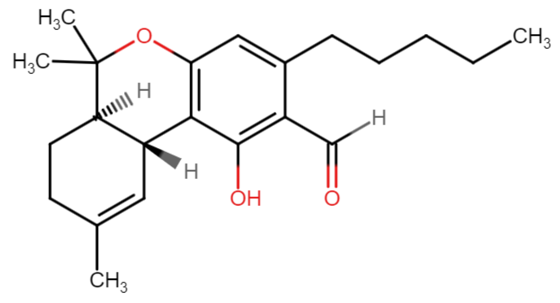

(-)-Δ^{9}-trans- tetrahydrocannabinal

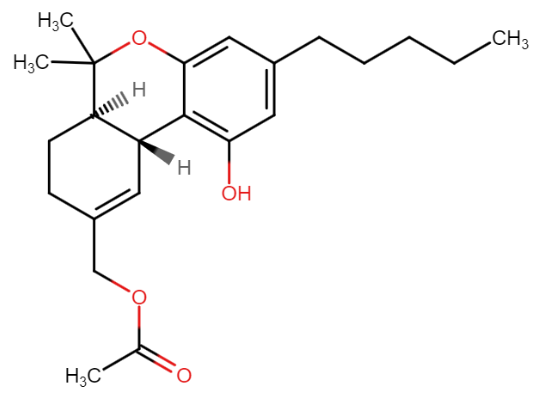

11-acetoxy-(-)-Δ^{9}-trans-tetrahydrocannabinolic acid A

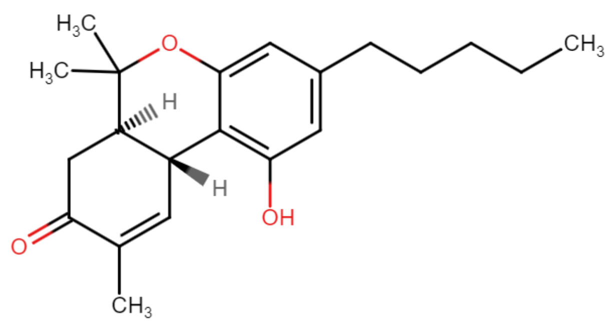

8-oxo-(-)-Δ^{9}-trans-tetrahydrocannabinol

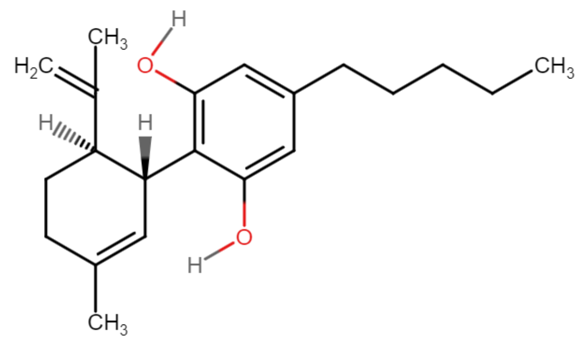

Cannabidiol

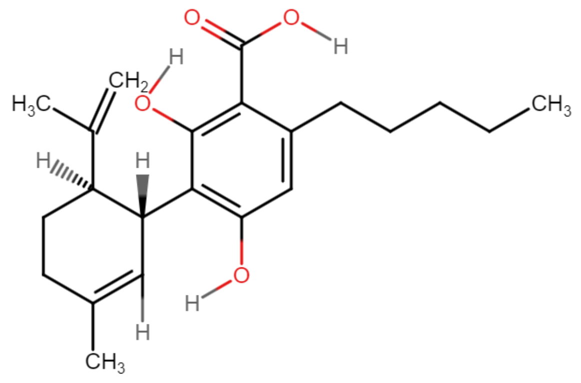

Cannabidiolic acid

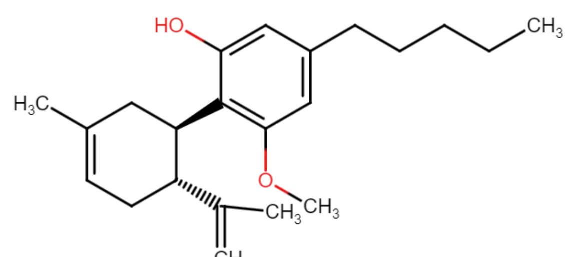

Cannabidiol monomethylether

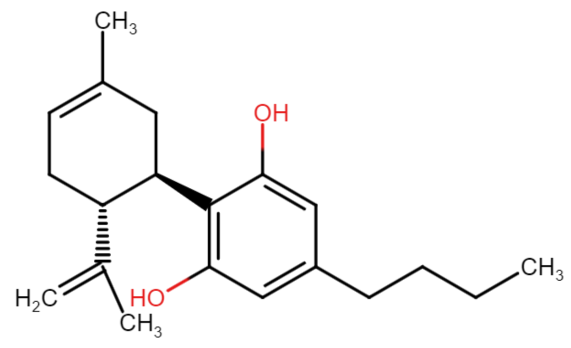

Cannabidiol-C_4

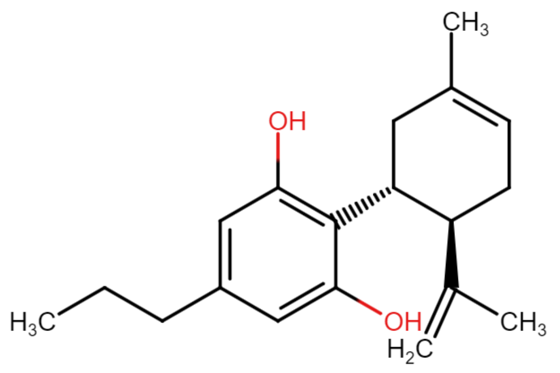

(-)-cannabidivarin

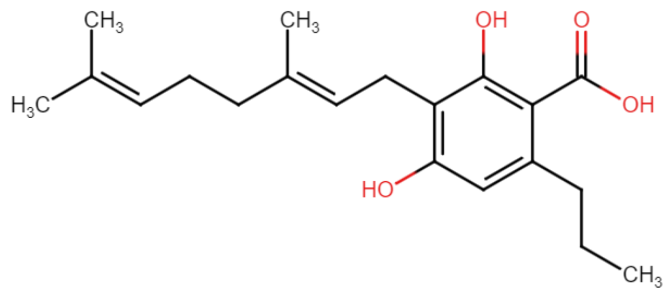

Cannabigerovarinic acid

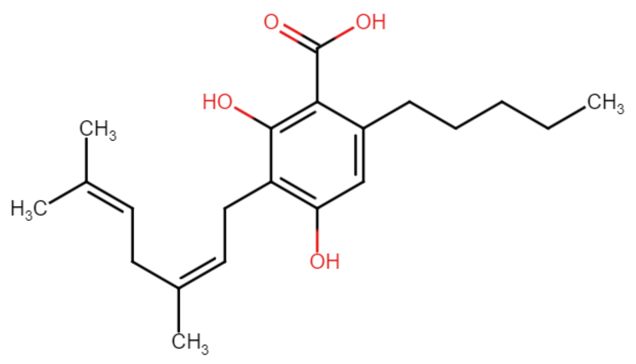

Cannabinerolic acid

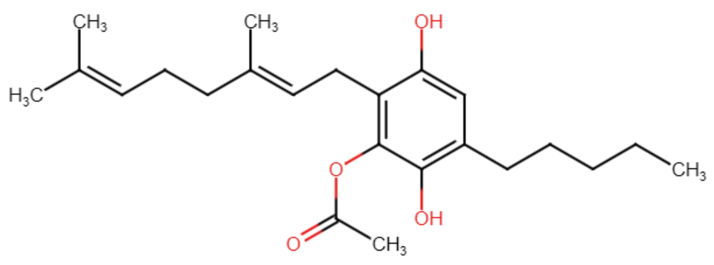

5-acetyl-4-hydroxy-cannabigerol

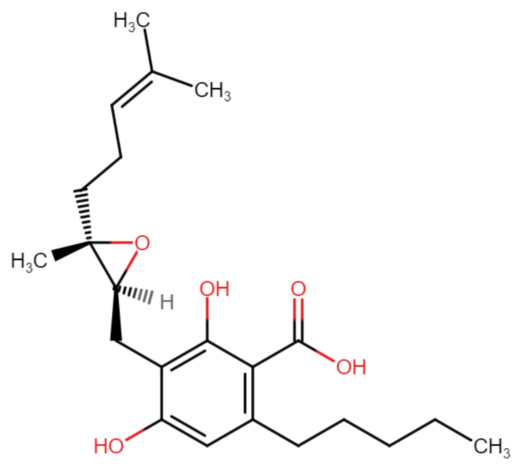

(±)-6,7-trans-epoxycannabigerolic acid

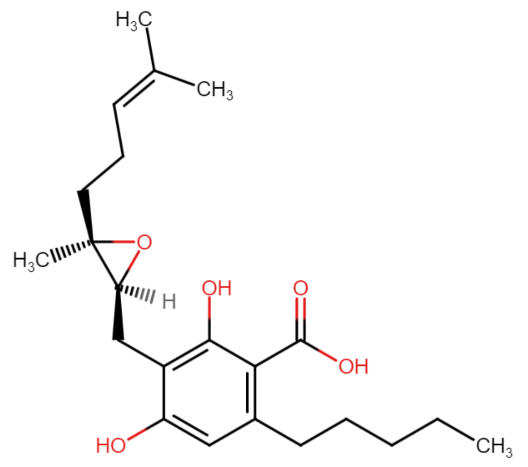

(±)-6,7-cis-epoxycannabigerolic acid

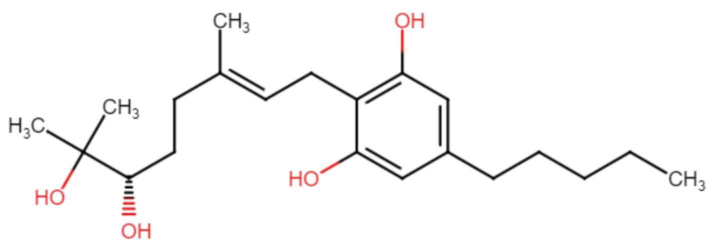

Camagerol

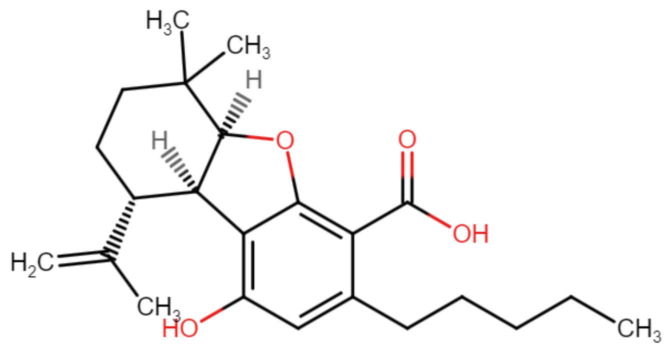

Cannabielsoin acid A

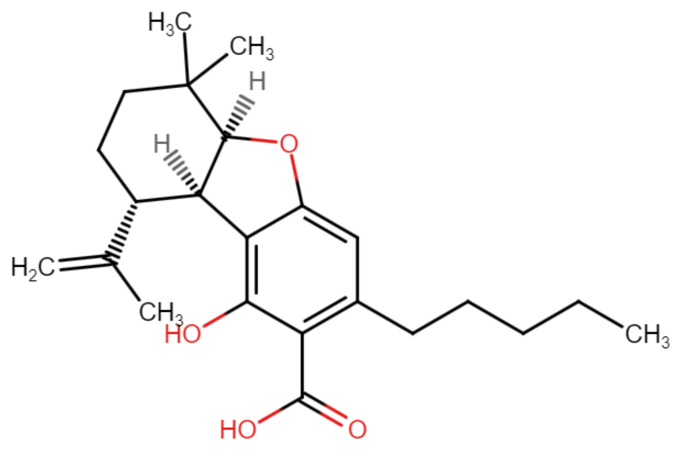

Cannabielsoin acid B

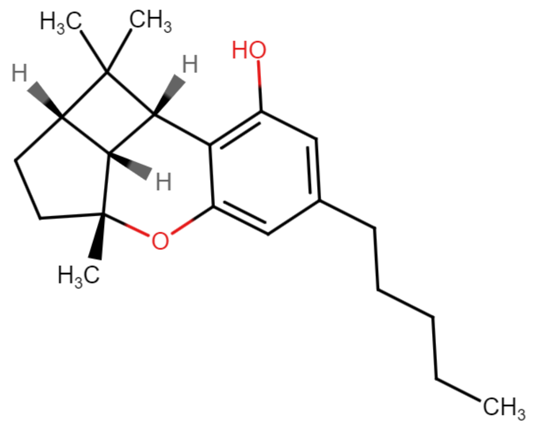

Cannabicyclol

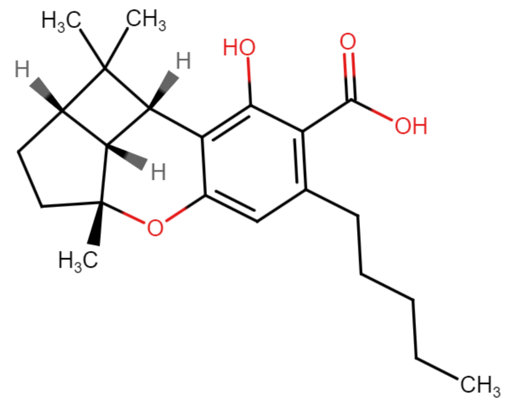

Cannabicyclolic acid

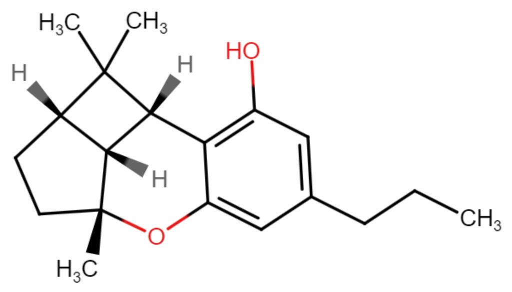

Cannabicyclovarin

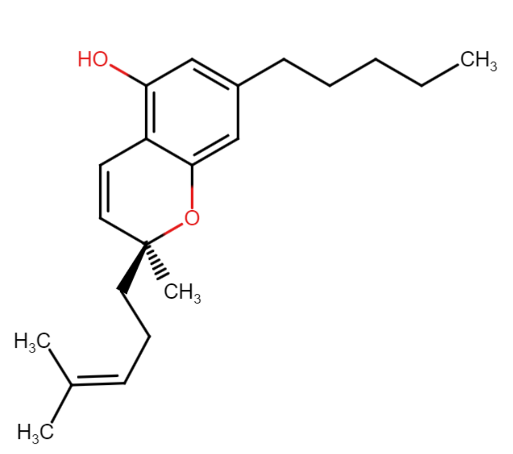

Cannabichromene

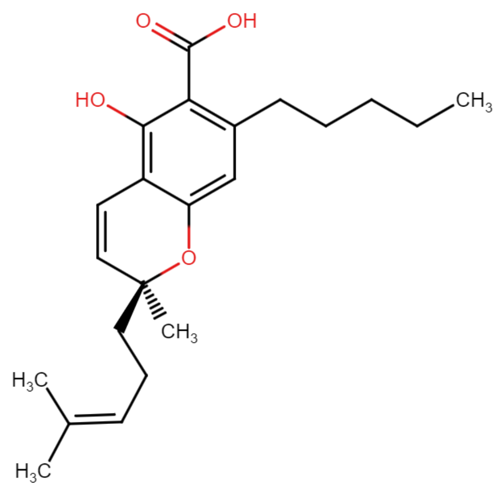

Cannabichromenic acid

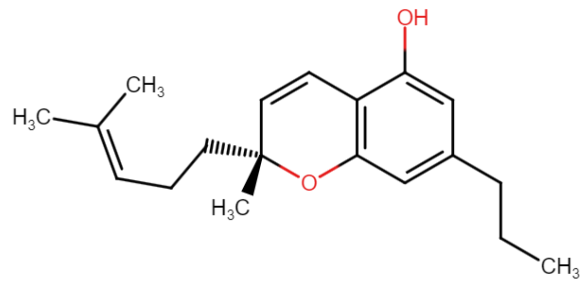

Cannabichromevarin

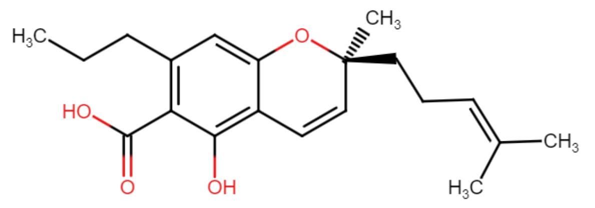

Cannabichromevarinic acid

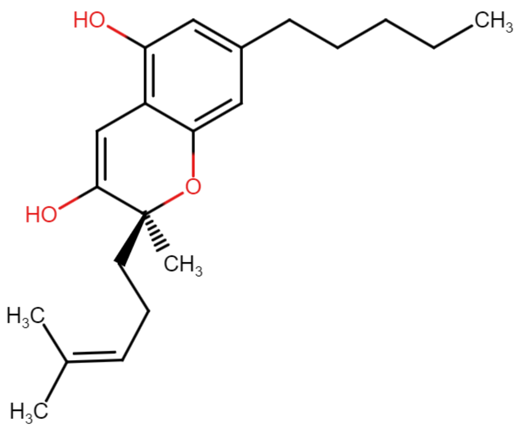

(-)-7-hydroxycannabichromene

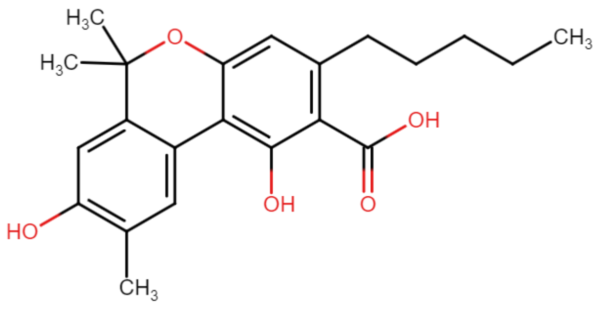

8-hydroxycannabinolic acid A

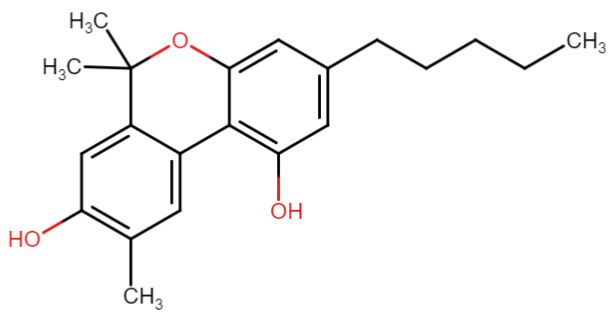

8-hydroxycannabinol

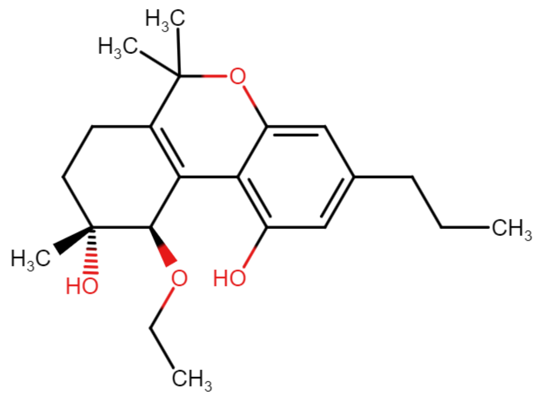

(-)-trans-cannabitriol-OEt-C_3

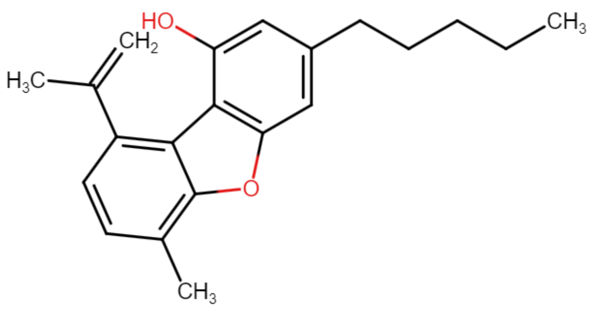

Dehydrocannabifuran

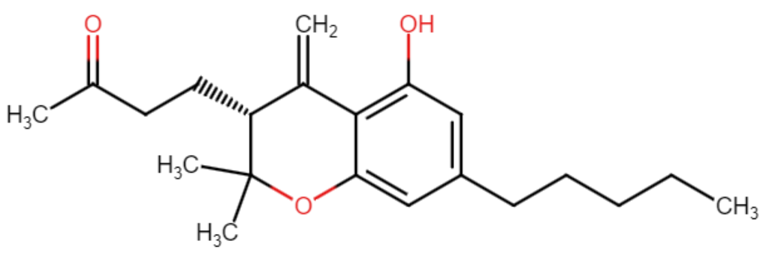

Cannabicoumaronome-C_5

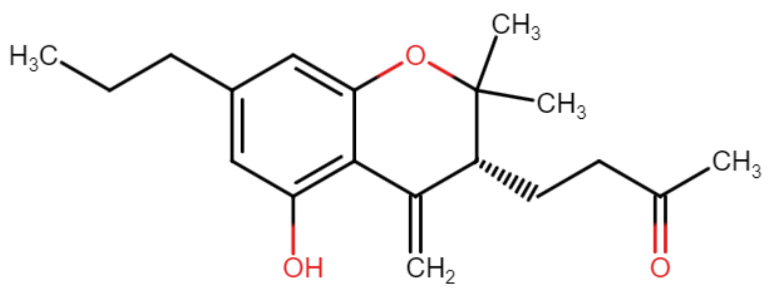

Cannabichromanone-C_3

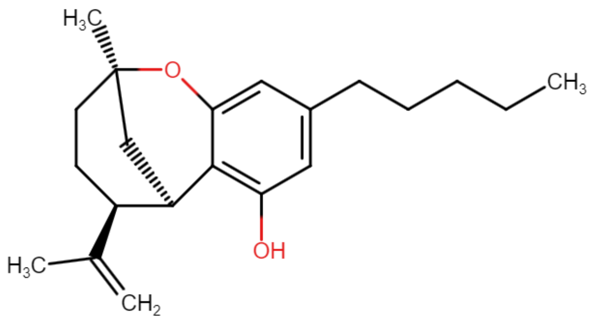

(-)-Δ^7-trans-(1R, 3R, 6R)-isotetrahydrocannabinol-C_5

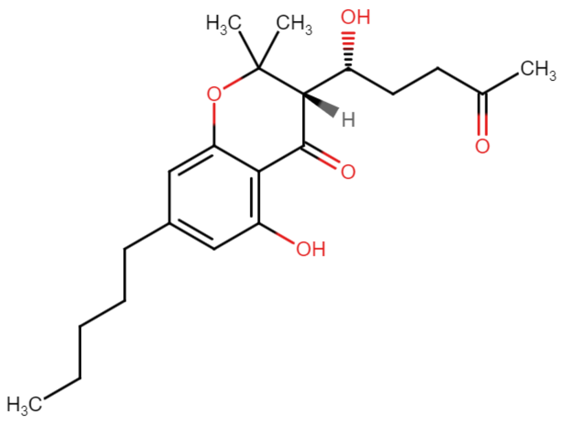

Cannabichromanone B

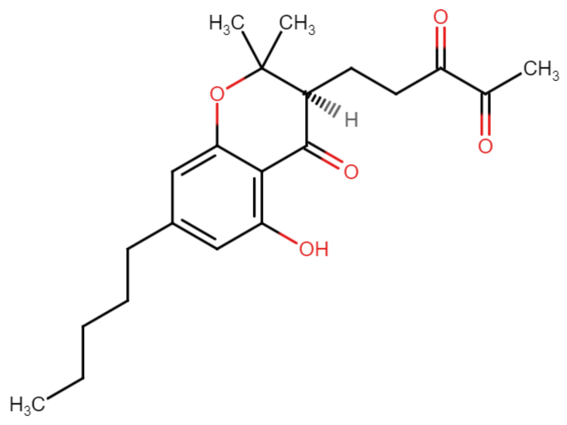

Cannabichromanone C

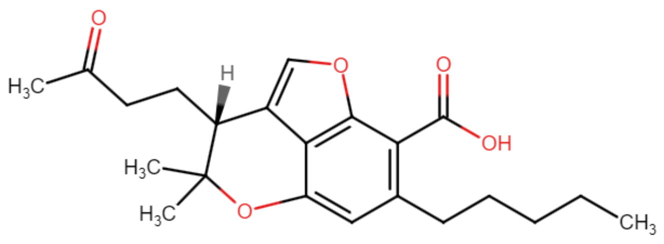

(-)-(7R)-cannabicoumaronic acid

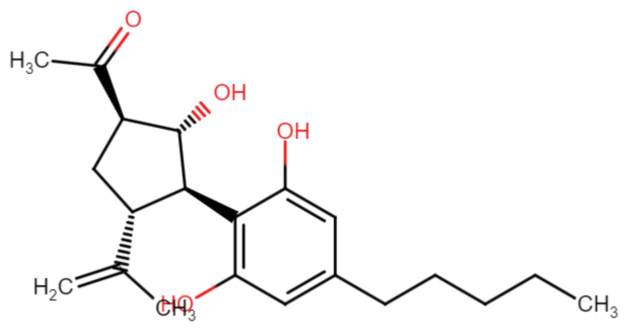

Cannabimovone

Supplementary Figure S4

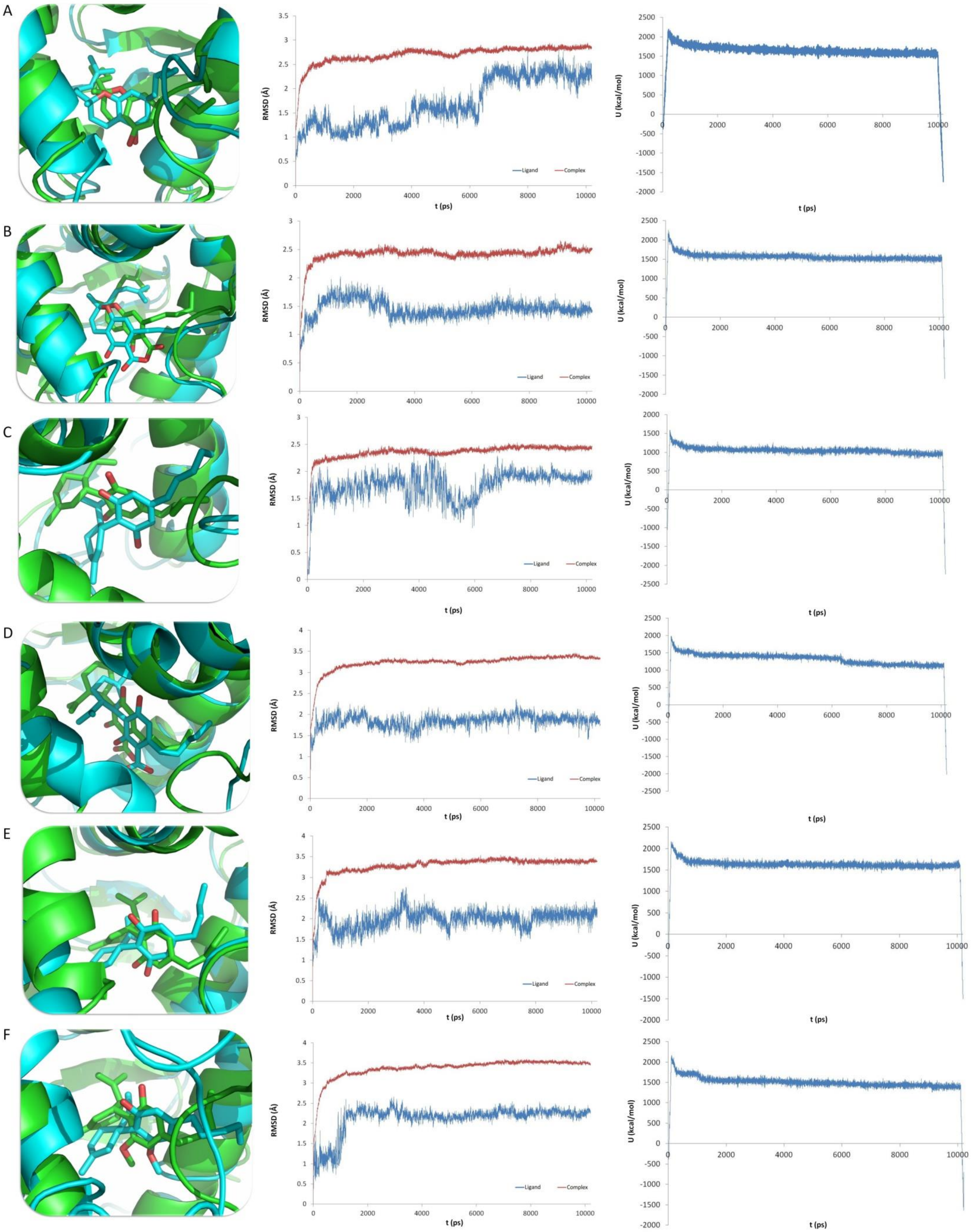

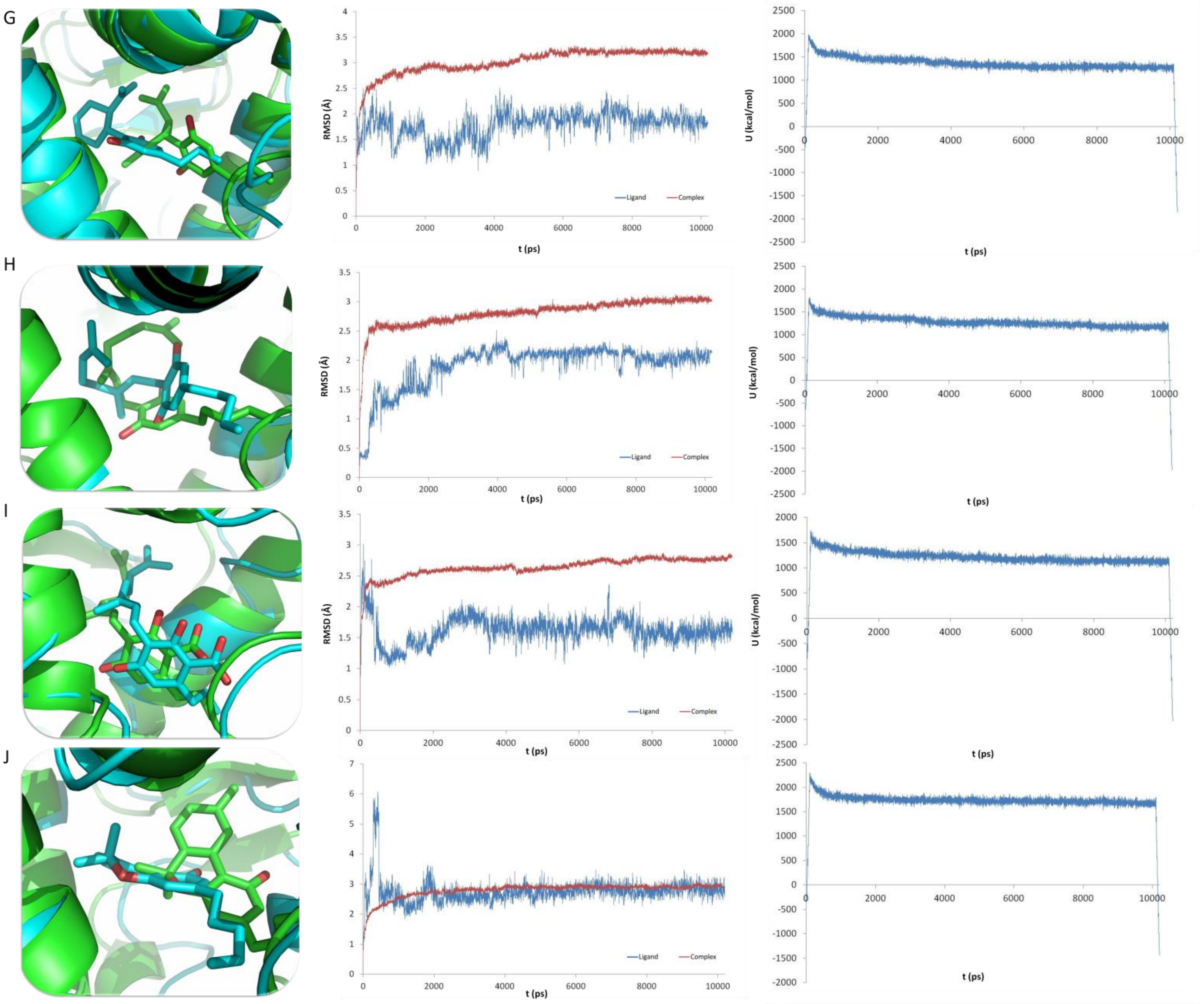
G
H
I
J
RMSD (Å)
t (ps)
U (kcal/mol)
Ligand
Complex

Supplementary Figure S5

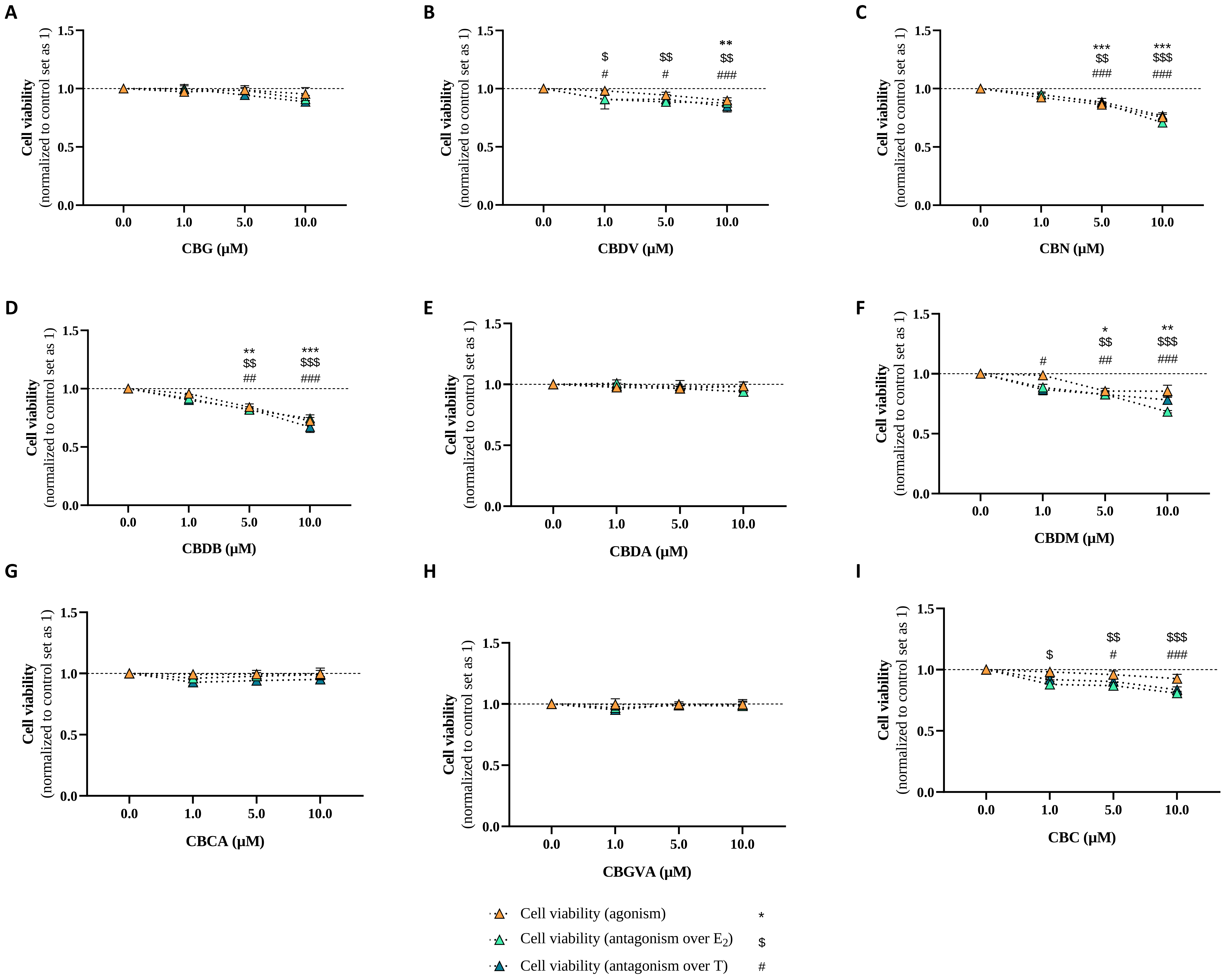

Supplementary Figure S6

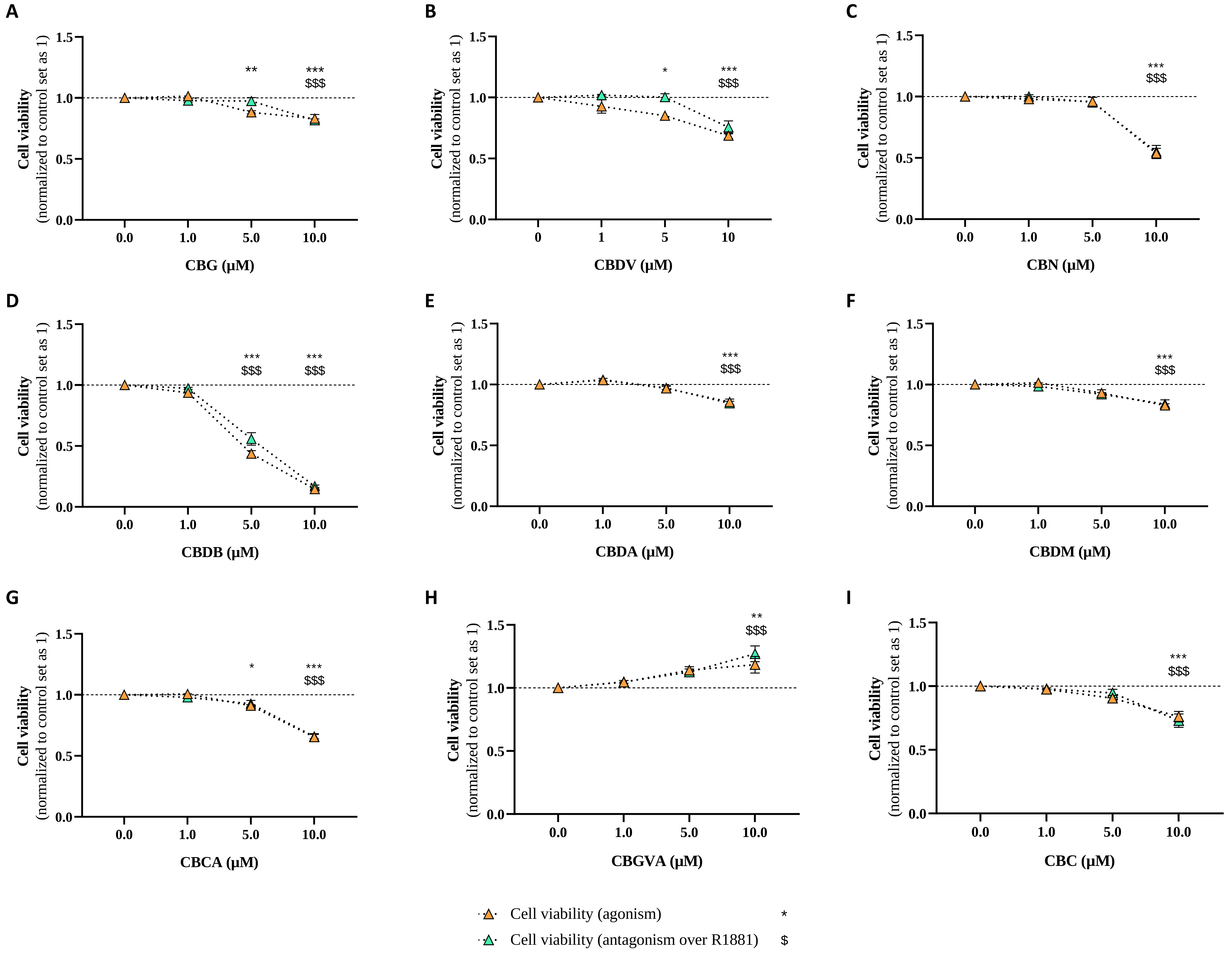

Supplement: Supplementary file 1 [file pharmaceuticals-17-01245-s001.zip › pharmaceuticals-3136354-supplementary.pdf]
